# Correlated Somatosensory Input in Parvalbumin/Pyramidal Cells in Mouse Motor Cortex

**DOI:** 10.1523/ENEURO.0488-22.2023

**Published:** 2023-05-05

**Authors:** Roman U. Goz, Bryan M. Hooks

**Affiliations:** Department of Neurobiology, University of Pittsburgh School of Medicine, Pittsburgh, PA 15213

**Keywords:** circuit mapping, long-range projections, motor cortex, optogenetic, parvalbumin interneurons, subnetworks

## Abstract

In mammalian cortex, feedforward excitatory connections recruit feedforward inhibition. This is often carried by parvalbumin (PV+) interneurons, which may densely connect to local pyramidal (Pyr) neurons. Whether this inhibition affects all local excitatory cells indiscriminately or is targeted to specific subnetworks is unknown. Here, we test how feedforward inhibition is recruited by using two-channel circuit mapping to excite cortical and thalamic inputs to PV+ interneurons and Pyr neurons to mouse primary vibrissal motor cortex (M1). Single Pyr and PV+ neurons receive input from both cortex and thalamus. Connected pairs of PV+ interneurons and excitatory Pyr neurons receive correlated cortical and thalamic inputs. While PV+ interneurons are more likely to form local connections to Pyr neurons, Pyr neurons are much more likely to form reciprocal connections with PV+ interneurons that inhibit them. This suggests that Pyr and PV ensembles may be organized based on their local and long-range connections, an organization that supports the idea of local subnetworks for signal transduction and processing. Excitatory inputs to M1 can thus target inhibitory networks in a specific pattern which permits recruitment of feedforward inhibition to specific subnetworks within the cortical column.

## Significance Statement

Incoming sensory information to motor cortex (M1) excites neurons to plan and control movements. This input also recruits feedforward inhibition. Whether inhibition indiscriminately suppresses cortical excitation or forms specific subnetworks is unclear. Specific differences in connectivity in circuits promoting different movements might assist in motor control. We show that input amplitudes to connected pairs of pyramidal (Pyr) excitatory neurons and parvalbumin (PV+) inhibitory interneurons is more strongly correlated than nonconnected pairs, suggesting the integration of interneurons into specific cortical subnetworks. Despite sparse connections between these cells, pyramidal neurons are vastly more likely (3×) to excite PV+ cells connected to them. Thus, inhibition integrates into specific circuits in motor cortex, suggesting that separate, specific circuits exist for recruitment of feedforward inhibition.

## Introduction

Feedforward excitation recruits feedforward inhibition in motor cortex (M1), but whether these inputs silence specific networks faithfully or connect promiscuously is unknown. M1 networks include distinct classes of excitatory pyramidal (Pyr) neurons, organized in layers (K.D. Harris and Shepherd, 2015; Matho et al., 2021) as well as inhibitory interneurons. Parvalbumin+ (PV+) interneurons are the largest group of inhibitory interneurons (∼40% of cortical inhibitory cells). PV+ cells are mostly fast-spiking basket cells, targeting the soma and proximal dendrites of excitatory cells, and chandelier cells, although other subtypes exist (Kawaguchi and Kubota, 1997; Kawaguchi and Kondo, 2002; S. Lee et al., 2010; Xu et al., 2010; Rudy et al., 2011; Pfeffer et al., 2013; Gouwens et al., 2020; Scala et al., 2021; Zhang et al., 2021; for review, see Tremblay et al., 2016).

These excitatory and inhibitory cells receive input from a range of cortical and thalamic sources (Hooks et al., 2013). Somatosensory cortex (S1) projects to topographically defined areas of the corresponding motor cortex (M1; Kaneko et al., 1994; Izraeli and Porter, 1995; Hooks et al., 2013; Zingg et al., 2014; J.A. Harris et al., 2019; Aruljothi et al., 2020). Vibrissal S1 (vS1) most strongly targets L2/3 and L5A neurons in vibrissal M1 (vM1; Mao et al., 2011). Thalamic projections from posterior thalamus (PO), a higher order somatomotor thalamic nucleus, arborize broadly across the tangential surface of cortex. In M1, these axons are laminarly restricted to layers 1 and the border of L2/3 and L5A (Izraeli and Porter, 1995; Morgenstern et al., 2016; J.A. Harris et al., 2019; for review, see Castro-Alamancos and Connors, 1997), although relative terminal size and density in these layers varies with cortical region (Audette et al., 2018; Casas-Torremocha et al., 2019). Functionally, PO inputs strongly excite L2/3 and L5A Pyr neurons (Hooks et al., 2013, 2015) as well as interneurons (Okoro et al., 2022).

Long-range projections can be cell type-specific (Hu and Agmon, 2016; Williams and Holtmaat, 2019). Both vS1 and PO target Pyr excitatory and inhibitory neurons, with layer-specific complementary activation of PV+ and somatostatin (SOM+) inhibitory neurons in M1 (Okoro et al., 2022). The organizational principles of cortical neurons studied to date in visual (Alonso and Martinez, 1998; Dantzker and Callaway, 2000; Alonso et al., 2001; Gonchar and Burkhalter, 2003; Song et al., 2005; Yoshimura and Callaway, 2005; Yoshimura et al., 2005; Brown and Hestrin, 2009; Bock et al., 2011; Kätzel et al., 2011; Ko et al., 2011, 2013, 2014; Glickfeld et al., 2013; Cossell et al., 2015; Wertz et al., 2015; W.A. Lee et al., 2016; Morgenstern et al., 2016; Palagina et al., 2019), somatosensory (Gibson et al., 1999; Shepherd and Svoboda, 2005; Shepherd et al., 2005; Kampa et al., 2006; Brown and Hestrin, 2009; Kätzel et al., 2011; Perin et al., 2011; Kim et al., 2016; Hayashi et al., 2018; Naka et al., 2019), auditory (Levy and Reyes, 2012; Li et al., 2014; Ji et al., 2016), frontal (Morishima and Kawaguchi, 2006; Morishima et al., 2011, 2017; Otsuka and Kawaguchi, 2008, 2009; Brown and Hestrin, 2009; Komiyama et al., 2010; Kätzel et al., 2011; Kiritani et al., 2012; Hira et al., 2013; Kells et al., 2019), and prefrontal (Wang et al., 2006; A.T. Lee et al., 2014) cortices suggest existence of subnetworks, a small number of neurons that have higher than random probability of connecting to each other compared with the surrounding cells (Mountcastle, 1997; Buxhoeveden and Casanova, 2002; Vegué et al., 2017). Subnetworks also share common excitatory inputs or long-range targets (Yoshimura and Callaway, 2005; Yoshimura et al., 2005; Wang et al., 2006; Brown and Hestrin, 2009; Perin et al., 2013). The development of specificity in subnetworks is enhanced by sensory experience in visual cortex (Ko et al., 2013, 2014). This organization may contribute to information propagation and neuronal computation (Nigam et al., 2016; Rost et al., 2018; Faber et al., 2019; Peron et al., 2020).

How inhibitory interneurons integrate in subnetworks may differ from Pyr neurons. Interneurons may connect nonspecifically to Pyr cells in nearby local, intralaminar circuits where they pool inputs from excitatory cells with different response properties (Fino and Yuste, 2011; Packer and Yuste, 2011; for review, see Mountcastle, 2003; Sohya et al., 2007; Fino et al., 2013).Thus, inhibitory interneurons have broader tuning curves for stimuli orientation and spatial frequency in visual cortex (Sohya et al., 2007; Niell and Stryker, 2008; Bock et al., 2011; Hofer et al., 2011), although inhibitory interneurons show selectivity in some species (Hubel and Wiesel, 1962, 1963; Ohki et al., 2005; but see Hirsch et al., 2003; Cardin et al., 2007; Ma et al., 2010; Runyan et al., 2010; Moore and Wehr, 2013; Ringach et al., 2016; Wilson et al., 2017). How to reconcile specific, connected subnetworks of excitatory cells with the nonspecific targeting of Pyr cells by interneurons? One possibility is that connectivity is dense, with high probability of connection, but synapse strength is weighted higher within subnetworks but weaker to outside networks (Znamenskiy et al., 2018). Another nonmutually exclusive possibility is that long-range inputs and outputs are organized into subnetworks during brain development through Hebbian plasticity (Tezuka et al., 2022; for review, see Katz and Shatz, 1996).

Here, we examined the functional organization of long-range thalamic (PO) and cortical (vS1) inputs to PV+ and Pyr cells in M1. Here, we refer to these sensory inputs as recruiting feedforward inhibition in M1, although thalamic input to cortex may also be referred to as feedback in other contexts. Using two-channel optogenetic stimulation with paired whole-cell patch-clamp recording, we show that thalamic and somatosensory inputs were more correlated in connected pairs compared with nonconnected pairs. Thus, recruitment of feedforward inhibition by thalamic and somatosensory inputs to motor cortex in mice is subnetwork specific and may depend on functional connections between excitatory and inhibitory cells. Specific differences in connectivity in circuits promoting different movements might assist in motor control.

## Materials and Methods

### Animals

Animal protocols were approved by the Institutional Animal Care and Use Committee at University of Pittsburgh. Experimental procedures were similar to previous studies (Okoro et al., 2022). Mice of either sex were used at postnatal day (P) ages P28–P123 (average, P46; median, P43; mode, P37; *N* = 123 mice, *n* = 133 slices, 1–10 cells per slice). PV+-Cre (The Jackson Laboratory, JAX 008069; Hippenmeyer et al., 2005; Scholl et al., 2015) or SOM-Cre (The Jackson Laboratory, JAX 013044; Taniguchi et al., 2011) mice were crossed to a lsl-tdTomato reporter line, Ai14 (The Jackson Laboratory, JAX 007914; Madisen et al., 2010) to label specific interneuron populations.

### Adeno-associated virus vectors

AAV2/1.CAG-hChR2-mCherry(H134R).WPRE, titer 1.4E13 (Addgene 100054; Mao et al., 2011) was injected into posterior thalamus (PO). AAV2/1.hSyn.ReaChR.mcit.WPRE.SV40, titer 2.52E13 (Addgene 50954; Lin et al., 2013) was injected into primary vibrissal somatosensory cortex (vS1).

### Stereotactic injections

Animals were anesthetized using isoflurane and placed in a custom stereotactic apparatus. Mice at P14–P40 were injected with AAV expressing excitatory opsins. Injections were made with glass pipettes (Drummond) using a custom-made injector (Narashige). The injection apparatus was a positive displacement pump allowing slow injection of nanoliter volumes. Injection coordinates (Table 1; Extended Data Fig. 1-2) on the anterior/posterior (A-P) axis are reported relative to bregma (positive values anterior to bregma); medial/lateral (M-L) axis coordinates are reported relative to the midline; and dorsal/ventral (D-V) axis coordinates are reported as depth from pia. Injections were made at two depths in cortex. For posterior thalamic injections, we used two adjacent sites, covering the elongated shape (in the A-P axis) of the PO nucleus. The more anterior set of those thalamic anterior injections was done in different mice (*n* = 15) for 11 connected and 12 nonconnected PV+ and Pyr pairs. Injections in both sites resulted in similar axon patterns in M1 (Hooks et al., 2013; for review, see Castro-Alamancos and Connors, 1997) and were pooled. As in our previous studies, we examined the injection site in thalamus during sectioning to confirm injection targeting to PO. We also confirmed the axonal projection pattern in cortex arborized in layer (L)1 and the L2/3–5A border, as is typical of PO injections (Petreanu et al., 2009; Hooks et al., 2013).

**Table 1:** Injection coordinates

Target	A-P	M-L	D-V	Volume
Posterior thalamus (PO)	−1.3	1.2	2.5/2.7/2.9	50–100 nl (3×)
	−0.8	1.3	2.6/2.75/3.0	50–100 nl (3×)
Primary somatosensory cortex (vS1)	−0.6	3.0	0.5/0.8	50–100 nl (2×)

Anterior/posterior (A-P) axis coordinates are reported relative to bregma (positive values anterior to bregma). Medial/lateral (M-L) axis coordinates are reported relative to the midline. Dorsal/ventral (D-V) axis coordinates are reported as depth from pia. Injections were made at two depths in cortex. Distances in millimeters (Extended Data Fig. 1-2), (Paxinos and Franklin, 2004; Hooks et al., 2013).

### Brain slice preparation

Brain slices were prepared >14 d after viral injection in young adult mice. Mice were anesthetized with isoflurane and the brain was rapidly removed and placed in cooled oxygenated (95% oxygen and 5% carbon dioxide) choline-based cutting solution (in mm: 110 choline chloride, 3.1 sodium pyruvate, 11.6 sodium ascorbate, 25 NaHCO_3_, 25 D-glucose, 7 MgCl_2_, 2.5 KCl, 1.25 NaH_2_PO_4_, and 0.5 CaCl_2_). Off-coronal sections (300 μm) of M1 were cut using a vibratome (VT1200S, Leica), rotated slightly from coronal to maintain apical dendrites of Pyr neurons intact in the slice plane. Additional sections were cut to confirm injection location. Slices were incubated at 37°C in oxygenated artificial CSF (ACSF; in mm: 127 NaCl, 25 NaHCO_3_, 25 D-glucose, 2.5 KCl, 2 CaCl_2_, 1 MgCl_2_, and 1.25 NaH_2_PO_4_) for >30 min and maintained at room temperature (22°C) thereafter. vM1 slices containing the brightest vS1 and PO axonal arborization were used for the patch-clamp electrophysiology (Extended Data Fig. 1-2).

### Electrophysiology and photostimulation

Whole cell recordings were performed at 22°C in oxygenated ACSF with borosilicate pipettes (3–6 MΩ; Warner Instruments) containing potassium gluconate-based internal solution (in mm: 128 potassium gluconate, 4 MgCl_2_, 10 HEPES, 1 EGTA, 4 Na_2_ATP, 0.4 Na_2_GTP, 10 sodium phosphocreatine, three sodium L-ascorbate; pH 7.27; 287 mOsm). Data were acquired at 10 kHz using an Axopatch 700B (Molecular Devices) and Ephus software (www.ephus.org; Suter et al., 2010) on a custom-built laser scanning photostimulation microscope with inversion recovery differential interference (Shepherd et al., 2003) using a Retiga 2000R camera (QICAM; QImaging). Slices were visualized with 4×, 0.16 numerical aperture, UPlanSApo; Olympus power objective. Individual neurons were visualized with a 60×, 1 numerical aperture Olympus Fluor LUMPlanFL water-immersion objective. Series resistance errors were minimized with bridge balance in current-clamp mode. Current-clamp recording was performed to confirm stable conditions. To measure excitability, 500-ms current steps were applied starting from −150 to 700 pA in 50-pA steps. Connections between pairs of cells were tested in current-clamp with a train of five 3-nA pulses of 0.5-ms duration repeated 40 times (20 for 5 Hz, 20 for 40 Hz) while the other cell was held in voltage-clamp mode. Each sweep length was 2 s with a 5-s delay between the sweeps. While testing connections from PV+ interneurons to Pyr cells, the Pyr cell was held at −50 mV (0 mV in some cases, *N* = 5 Pyr cells) to detect IPSCs, while the reciprocal connection was tested with PV+ interneurons held at −70 mV to detect EPSCs.

Photostimulation was done as previously described (Hooks et al., 2015) using 590- and 470-nm LEDs (OptoLED, Cairn). Photostimuli in single-channel experiments were ∼2 mW/mm^2^. Photon flux was matched for 590-nm and 470-nm stimuli in the same experiment. Light <585 nm from the 590-nm LED was blocked using a bandpass filter (D607/45, Chroma). Voltage-clamp experiments with LED photostimulation were performed at −70 mV for Channelrhodopsin-induced EPSCs and at 0 mV for Channelrhodopsin-induced IPSCs recruited through feedforward inhibition. Under these conditions, 590-nm LED pulses of 50, 100, 250, and 500 ms were followed by 50-ms pulses of 470-nm LED. Depolarized ReaChR-expressing and ChR2-expressing axons, respectively, triggered the local release of glutamate. Sweeps were repeated four times with a 20-s gap.

The four different delay protocols (50–500 ms) were used as a control for the effects of activation with two different wavelengths (590 and 470 nm) on the glutamate release caused by activation of red-shifted ReaChR channels first and the blue shifted ChR2 second. If the first stimulus (590 nm) does not affect the features of the second response, then we are confident that our paradigm is independently stimulating the two pathways. In some experiments, biocytin was added to the intracellular solution (3 mg/ml biocytin or neurobiotin). The experimental recording sequence started with series resistance test of five pulses in voltage-clamp, followed by current-clamp to test action potential (AP) firing and confirm the passive and active electrophysiological properties. This was followed by the connectivity test between the pairs and finally, optogenetic stimulation at different holding potentials in voltage-clamp. Cells with stable access were used for quantification of passive electrophysiological properties.

### Histologic preparations and image analysis

Some of the slices were processed for biocytin recovery. Samples were postfixed overnight in 4% PFA. Sections were processed as free floating and stained with Hoechst reagent. Blocking was done in TBS containing 10% normal goat serum or normal donkey serum (MilliporeSigma) and 0.5% Triton X-100 (MilliporeSigma) for 2 h at room temperature. Tissue was washed three times in TBS with 2% normal goat serum and 0.4% Triton X-100 (washing solution), followed by incubation with streptavidin conjugated Alexa-647 (1:200) overnight to 24 h at 4°C in the washing solution. After overnight incubation the tissue was stained with nuclear staining Hoecsht (10 mg/ml in water, 1:3000 dilution; Invitrogen) for 10 min and washed four times for 10 min. Tissue was mounted on Fisherbrand ColorFrost Plus microscope slides submerged in Fluoromount G (ThermoFisher).

Images were acquired with a Nikon A1R confocal microscope with 20× or 60× oil-immersion objectives. All images were processed in the ImageJ-Fiji package. Image processing for publication was done in Fiji and Corel Draw Graphics Suite X8 (Corel) or Adobe Illustrator.

### Experimental design and statistical analysis

Data analysis was performed with custom routines written in MATLAB. Electrophysiology data were low pass filtered (1 kHz) with an 8-pole low-pass Bessel filter. EPSCs were detected with a threshold of >2× SD from baseline. All data measurements were kept in Microsoft Excel (Microsoft) and in Origin (OriginLab). Statistical analysis of the data was done in SPSS v.24-v.28 (IBM). For large samples, one-way ANOVA with Tukey’s *post hoc* correction was used. When the samples had nonhomogeneous variance (significant Levene’s test for equality of variance), Welch’s test with Games–Howell *post hoc* correction was used. For small samples from different observations, independent-samples two-tailed Student’s *t* test was used, and depending on Levene’s test significance, the *t* statistics for equal or unequal variance are reported. For measurements coming from the same neurons before and after treatment, paired-samples two-tailed Student’s *t* test was used. For non-normally distributed data, the nonparametric Wilcoxon signed-rank or Kolmogorov–Smirnov tests were used. All data are shown as arithmetic average ± SEM or ±95% confidence intervals, unless otherwise specified.

### Code accessibility

Data analysis was performed with custom routines written in MATLAB. The acquired data as well as the data acquisition and analysis software (M-files in MATLAB format) are available on request.

## Results

**Figure 1.**
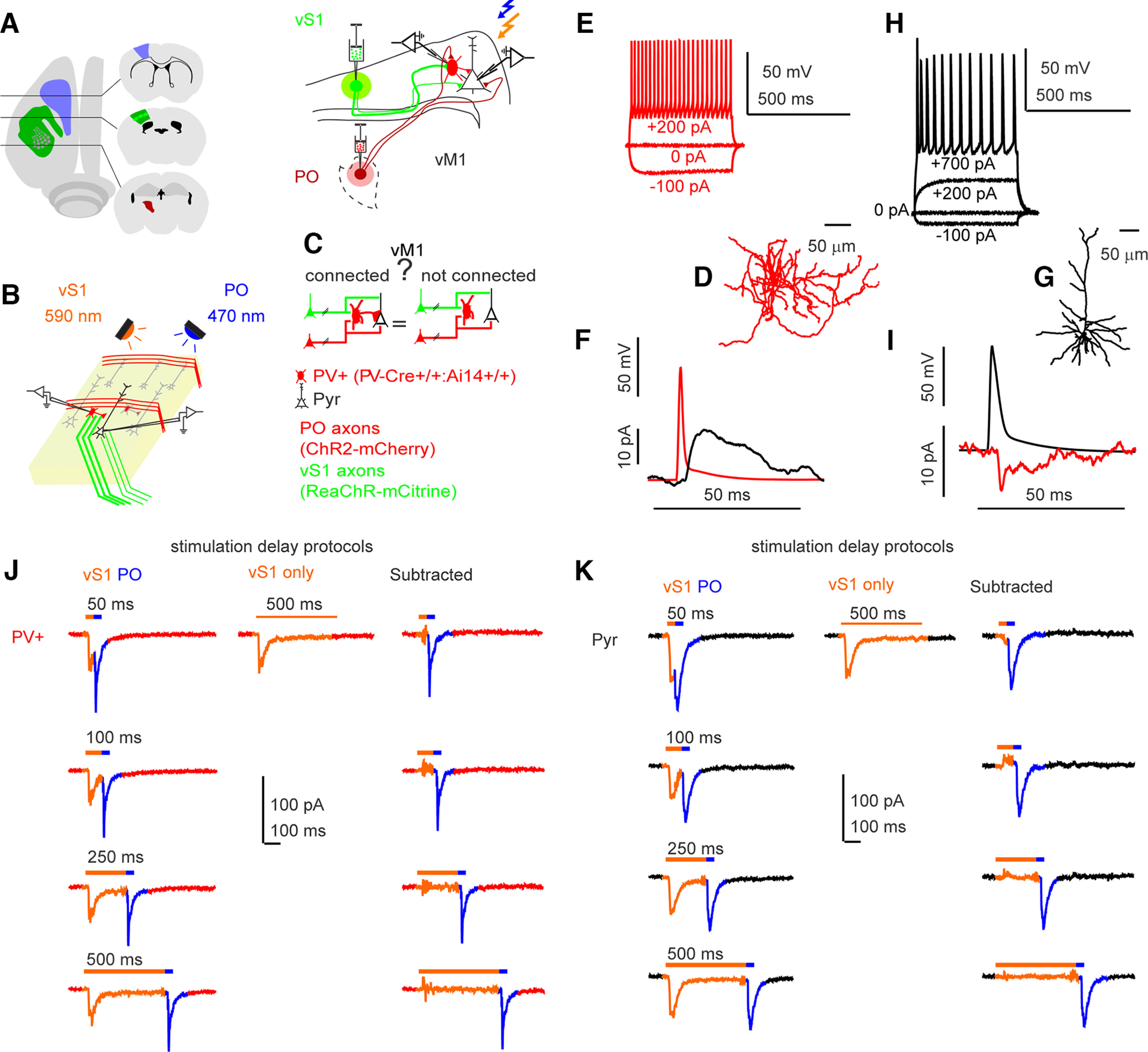
Long-range thalamic and cortical projections in mouse brain slices. ***A***, Targeted regions (left panel). Illustration of the slice preparation (right panel). Vibrissal M1 (vM1) receiving long-range projection inputs from vS1 (green lines, ReaChR-mcitrine expressing), and from posterior thalamus (red lines, ChR2-mcherry expressing). Paired whole-cell patch-clamp recording targeted to a pyramidal excitatory neuron (Pyr, empty triangular shaped) and parvalbumin positive inhibitory interneuron (PV+, oval shaped, red) receiving inputs from vS1 (stimulated with orange LED, 590 nm), and from posterior thalamus (stimulated with blue LED, 470 nm). ***B***, Illustration of stimulation paradigm in brain slices. ReaChR expressing axons (vS1, green) were stimulated first with 590 nm, orange LED (50–500 ms) immediately followed by stimulation of ChR2 containing axons (PO) with 470-nm blue LED 50 ms with equal light intensity (∼2 mW/mm^2^). Example traces are shown in ***J–K*** containing axons (PO) with 470-nm blue LED 50 ms with equal light intensity (∼2 mW/mm^2^). Example traces are shown in ***J–K***. ***C***, Illustration of the scientific inquiry question and color coding. ***D***, Example reconstruction of a recorded biocytin filled PV+ cell. Scale bar above. ***E***, Example current-clamp traces showing responses of a PV+ inhibitory fast-spiking cell recorded in those experiments with current steps in between and the scale bar to the right. For electrophysiological properties, please see Extended Data Figure 1-1. Extended Data Table 1-1. ***F***, Example traces of a connectivity test between a PV+ cell in current-clamp mode with 3 nA 0.5-ms current step to elicit single AP and the voltage-clamp IPSC response of the Pyr cell held at 0 mV. ***G***, Example reconstructed biocytin filled Pyr cell. Scale bar above. ***H***, Example current-clamp recording of Pyr cell with current steps as labeled. Scale bar to the right. ***I***, Example of connectivity test, with the same protocol as in ***E***, Pyr in current-clamp, EPSC in PV+ cell held at −70 mV. ***J***, ***K***, Examples of two connected PV+ and Pyr cells in voltage-clamp showing responses to LED stimulation. First column 590-nm and 470-nm LED stimulation (colored bars indicate time of LED on/off). Middle traces are 500-ms 590-nm alone. Third column is a subtraction of middle traces from the first column to reveal 470-nm response.

Subnetworks by definition contain cells connected to each other more frequently than to cells in the outside networks. We hypothesized that long-range synaptic inputs may be different to cells in one subnetwork compared with a different subnetwork. To understand how long-range projections to primary vibrissal motor cortex (vM1) excite specific networks of interneurons, we recorded from connected and nonconnected pairs of parvalbumin positive inhibitory interneurons (PV+) and Pyr excitatory neurons (Pyr) to explore differences in their circuit connectivity. We used the two-channel Channelrhodopsin-assisted circuit mapping (2CRACM) approach developed by our lab (Petreanu et al., 2007, 2009; Hooks et al., 2015). We injected viral vectors containing Channelrhodopsin-2 (ChR2-mCherry) into posterior thalamus (PO) and the red-shifted Channelrhodopsin variant ReaChR (ReaChR-mCitrine) into vS1. We activated these opsins by sequential 590-nm and 470-nm stimulation (Fig. 1*A*,*B*,*J*,*K*). The intracranial injections were done with a custom-made positive displacement system in PV+-Cre^+/+^;lsl-tdTomato (ai14)^+/+^ mice. To allow for opsin expression, recording started two weeks after injections. Pairs of adjacent (<120 μm) PV+ and Pyr neurons were recorded in whole-cell current-clamp and voltage-clamp configuration. Passive membrane properties and series resistance were measured at −70 mV. Current injections of 500 ms in 50-pA steps characterized active membrane properties including AP firing (Fig. 1*E*,*H*; Extended Data Fig. 1-1; Extended Data Table 1-1). Connectivity was tested in each direction (PV+ ↔ Pyr), holding one neuron in current-clamp and applying 3 nA, 0.5-ms current steps while the other neuron was held in voltage-clamp (Fig. 1*F*,*I*). Some of the slices were processed for biocytin recovery at the end of the experiments, which allowed confirmation of cell type and laminar position (Fig. 1*D*,*G*). Input was quantified in voltage-clamp using the 2CRACM approach, ReaChR-mCitrine was stimulated with 590-nm LED light (50- to 500-ms pulses), followed by stimulation of ChR2-mCherry with 470-nm blue LED light (50-ms pulses immediately following), with additional 500-ms 590-nm only LED stimulation to have ReaChR trace only for subtraction (Fig. 1*J*,*K*).

10.1523/ENEURO.0488-22.2023.f1-1 Extended Data Figure 1-1 Passive and active membrane properties in PV+ and Pyr neurons. ***A***, Example current-clamp traces from PV+ (red) with current steps shown in between, resting membrane potential (RMP) to the left measured before current step application, SAG is the negative deflection below resting state to the negative current steps in percent; action potential (AP) height is measured from RMP; the rheobase is the minimal voltage required to make the neuron fire AP (300 pA); number of APs at rheobase is also recorded; overshoot is measured at the end of 500 ms negative current steps. ***B***, Example current-clamp traces from Pyr (rheobase 300 pA). ***C***, Example APs from PV+ and Pyr showing 10–90% rise time, voltage threshold (Vthr), fast afterhyperpolarization (fAHP); 100–50% decay time; and AP half-height width. ***D***, The APs from ***C*** are converted to phase-space plot to show the 50 V/s voltage threshold, 0.1 dv/dt height voltage threshold; max rise slope; max decay slope. ***E***, Input-output curve showing number of APs (average ± SEM) fired by the cells in response to the 500-ms current steps. ***F–U***, Membrane properties. Download Figure 1-1, TIF file.

**Figure 2.**
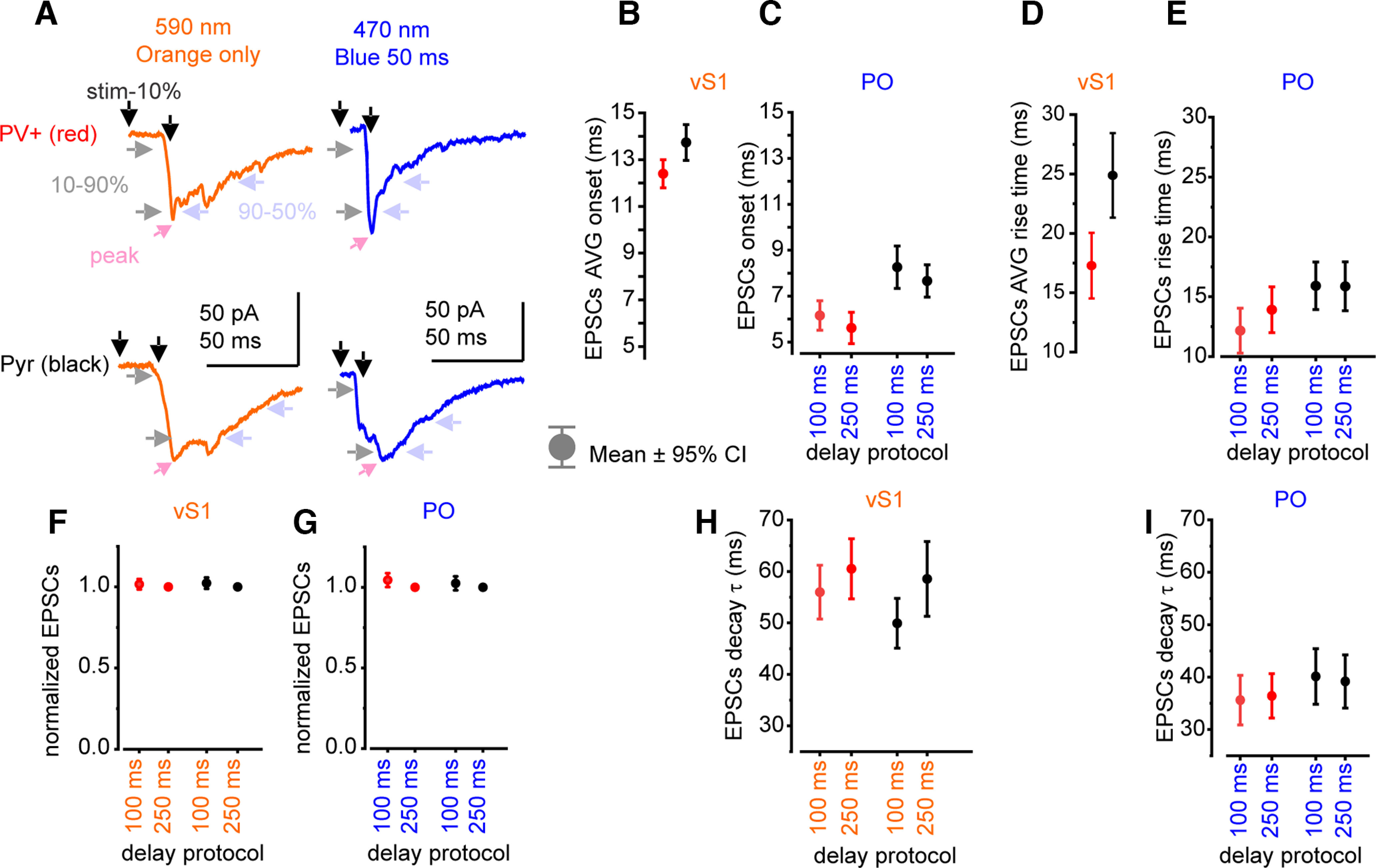
2CRACM EPSCs kinetics. ***A***, Example response traces of PV+ (red) and Pyr (black) to 590 nm (vS1, orange) LED stimulation, following by 470 nm (PO, blue) LED stimulation. Description of kinetics that were measured and compared, as indicated by arrows in the panel. Onset was measured from start of the stimulation to 10% of the EPSC peak, the rise time was measured from 10% to 90% of the EPSC peak; and the decay time was measured from 90% to 50% of the EPSC peak. Peaks are shown by pink arrowheads. Because vS1 responses onset before PO responses, onset and rise kinetics are averaged across all delay protocols. For the uniformity purposes only, 100-ms and 250-ms delay protocols kinetics are shown to constrain to the data analyzed and presented in other figures. ***B***, vS1 EPSC onset averaged across all delay protocols (5–500 ms; PV+ *n* = 123–125; Pyr *n* = 114–115). ***C***, PO EPSC onset (PV+ *n* = 84–108; Pyr *n* = 85–109). ***D***, vS1 EPSC averaged rise time (PV+ *n* = 123–125; Pyr *n* = 114–115). ***E***, PO EPSC rise time (PV+ *n* = 84–108; Pyr *n* = 85–109). ***F***, vS1 EPSC normalized to its own peak at 250-ms delay protocol response (PV+ *n* = 123–125; Pyr *n* = 114–115). ***G***, PO EPSC normalized to its own peak at 250-ms delay protocol response (PV+ *n* = 89–110; Pyr *n* = 118). ***H***, vS1 EPSC decay time (PV+ *n* = 59–110; Pyr *n* = 47–102). ***I***, PO EPSC decay time (PV+ *n* = 76–98; Pyr *n* = 81–100). Means are shown with 95% confidence intervals.

10.1523/ENEURO.0488-22.2023.f1-2 Extended Data Figure 1-2 Injections sites and axonal projections. ***A***, Targeted regions (left panel). ***B***, Example of an off-coronal 300-μm-thick brain slice of vM1 with two cells in patch-clamp. The location of the cells relative to pia and white matter. Approximate layer boundaries indicated. Second column vS1 injection site and axonal projection fluorescence in green (ReaChR-mcitrine). Third column PO injection site (ChR2-mCherry) and axonal projections fluorescence in red in L1 and L2/3 and L5A border (top, vM1 PO projections image is taken from another slice and stretched over the current slice, expressing ReaChR-mcitrine pseudo-colored in red). Fourth column is an overlay of all the previous columns and the shape of thalamic nuclei from mouse brain atlas (Paxinos), also shown in ***D***. ***C***, Illustration of vM1 slice with PO thalamic (red) and vS1 (green) axonal projections. ***D***, Illustration of the thalamic nuclei shapes taken from Paxinos mouse brain atlas coordinates bregma −1.34 mm, −2.3 mm, −2.46 mm. Scale bars are 500 μm. Download Figure 1-2, TIF file.

10.1523/ENEURO.0488-22.2023.tab1-1 Extended Data Table 1-1 Intrinsic Cell Properties of PV+ and Pyr Neurons in M1. Download Table 1-1, DOCX file.

**Figure 3.**
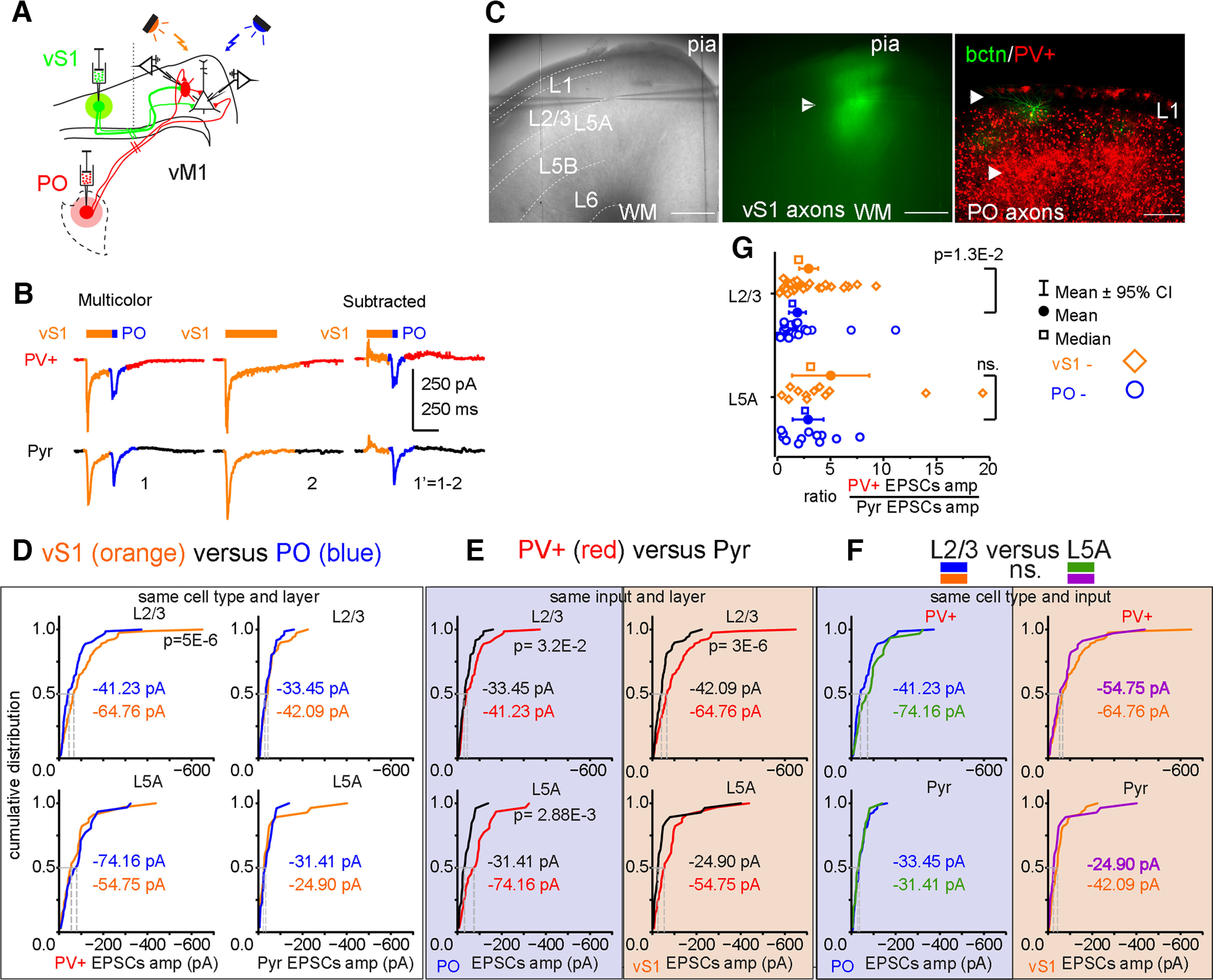
vS1 input is larger than PO input to vM1 layer 2/3 PV+ interneurons. ***A***, Illustration of the vM1 slice preparation receiving long-range projections inputs from vS1 (green lines, ReaChR-mcitrine) and from PO (red lines, ChR2-mcherry) during paired recording. ***B***, Example voltage-clamp traces in cells held at −70 mV from a pair of PV+ inhibitory interneuron and Pyr excitatory neuron showing responses to 590-nm LED stimulation of vS1 axons in vM1 immediately followed by 470-nm LED stimulation of PO axons. Middle panel is the response to 590-nm LED stimulation alone and the last panel is a subtraction of the middle traces from the first traces to isolate 470 nm-induced EPSCs. ***C***, Example of an off-coronal 300-μm brain slice of vM1 with two cells in patch-clamp. Left panel shows the location of the cells relative to pia and white matter. Approximate layer boundaries indicated. Middle panel shows vS1 axonal fluorescence in green (arrowhead). Right panel is a confocal image showing PO axonal fluorescence in red (arrowheads) in L1 and the lower L2/3 and L5A together with PV+ interneurons. A biocytin filled example pair (green) of cells is shown. Scale bars are 500 μm. ***D***, Cumulative distribution of EPSC amplitudes comparing vS1 and PO inputs to PV+ (left) and Pyr (right) neurons in layers 2/3 (top) and 5A (bottom). EPSCs in L2/3 PV+ neurons were significantly larger from vS1 than PO (Wilcoxon signed-rank test = 2131, *p* = 5E-6, effect size η^2^ = Z^2^/(*n*−1), η^2^ = 0.296). Differences between vS1 and PO EPSCs in L2/3 Pyr neurons were significant (Wilcoxon signed-rank test = 620, *p* = 1.63E-4, effect size, η^2^ = 0.203; however, the effect size Hedges’ *g* and confidence interval show no statistical significance, Extended Data Table 1-2; Hedges’ *g* = 0.367, *p* = 0.1 [95.0%CI −0.0344, 0.728]). PV+ and Pyr EPSCs amplitudes in L5A between VS1 and PO were significantly different (PV+: Wilcoxon signed-rank test = 1, *p* = 1E-6, η^2^ = 0.78, however the effect size Hedges’ *g* and confidence interval show no statistical significance, Extended Data Table 1-2; Hedges’ *g* = 0.177, *p* = 0.59, [95.0%CI −0.482, 0.888]; Pyr: Wilcoxon signed-rank test = 28, *p* = 1.8E-4, η^2^ = 0.56, Hedges’ *g* = 0.389, *p* = 0.228, [95.0%CI −0.149, 0.823]). ***E***, Cumulative distribution of EPSC amplitudes comparing input to Pyr and PV+ neurons from PO (left) and vS1 (right) to neurons in layers 2/3 [top, Mann–Whitney (M–W) *U* = 4242 and 3087 (PO), *p* = 3E-6, η^2^ = 0.143 and 3.2E-2 (PO), η^2^ = 0.032] and 5A (bottom, M–W *U* = 663 and 589 (PO), *p* = 8.17E-3, η^2^ = 0.115, and 2.88E-3 (PO), η^2^ = 0.159; however, the effect size for vS1 L5A, Hedges’ *g* and confidence interval, show no statistical significance, Extended Data Table 1-2; Hedges’ *g* = 0.31, *p* = 0.22, [95.0%CI −0.338, 0.767]). ***F***, Cumulative distribution of EPSC amplitudes comparing L2/3 and L5A inputs to PV+ (top) and Pyr (bottom) from PO (left) and vS1 (right) was not significantly (ns) different (PO L2/3 to L5A PV+: M–W *U* = 863, *p* = 6.9E-2, η^2^ = 0.032; L2/3 to L5A Pyr: M–W *U* = 949, *p* = 8.3E-1, η^2^ = 0.0005; VS1 L2/3 to L5A PV+: M–W *U* = 1531, *p* = 4.1E-1, η^2^ = 0.006; L2/3 to L5A Pyr: M–W *U* = 1329, *p* = 1.1E-1, η^2^ = 0.024). ***G***, Ratio of vS1 and PO EPSCs amplitudes in PV+ interneurons divided by the EPSCs amplitudes in Pyr neurons (from ***D–F***) shows that vS1 preferentially targets PV+ neurons compared with PO confirming the results of previous study with subcellular CRACM (Okoro et al., 2022). Only pairs with both inputs included. L2/3 vS1 and PO pairs, *n* = 30; L5A, *n* = 12. Wilcoxon signed-rank test (L2/3 ratio of VS1 vs PO Wilcoxon signed-rank test = 112, *p* = 1.3E-2, η^2^ = 0.212, L5A ratio of VS1 vs PO Wilcoxon signed-rank test = 22, *p* = 1.82E-1, η^2^ = 0.162) was used since the synaptic responses are not normally distributed Kolmogorov–Smirnov and Shapiro–Wilk tests for normality [L2/3 PO inputs (PV+/Pyr) K-S(12) = 0.312, *p* = 2.02E-3, S-W(12) = 0.7, *p* = 8.35E-4; L5A vS1 inputs (PV+/Pyr), K-S(12) = 0.34, *p* = 4.15E-4, S-W(12) = 0.718, *p* = 1.26E-3]. Means are shown by circles and the medians by squares. Whiskers represent 95% confidence intervals.

10.1523/ENEURO.0488-22.2023.tab1-2 Extended Data Table 1-2 Estimation statistics. The website that was used to calculate Hedges’ *g* and confidence interval with 5000 bootstrap samples, the confidence interval is bias corrected. https://www.estimationstats.com/#/; Ho et al., 2019). η^2^ Calculated for nonparametric tests, η^2^ = Z^2^/(n−1) and captures % of variance of one variable reflected by the test results comparing it to another variable (0.296 -> 29.6%). While Hedges’ *g* estimates by how many standard deviations the two variables differ (1, means 1 SD). For Figure 3*D*,*G*, paired test was used on the website. For connected versus not connected Fisher’s exact test, the probability of not connected was subtracted from the probability of connected to calculate the effect size. Download Table 1-2, DOCX file.

The Channelrhodopsin-induced EPSCs kinetic properties are shown in Figure 2. These include the EPSC onset delay, the rise time, normalized amplitude, and decay time course. EPSC onset times may be slightly slower for vS1 inputs compared with PO inputs, because of the slower kinetics of ReaChR (Lin et al., 2013). However, these do not vary significantly across delay times, suggesting that activating vS1 inputs first does not affect responses from PO afferents.

### Somatosensory cortical excitation is stronger than thalamic input to PV+ neurons in layer 2/3 of vM1

To compare the difference in recruitment of feedforward inhibition mediated by PV+ cells and excitation of Pyr cells, we recorded opsin-mediated EPSCs in pairs of PV+ and Pyr cells in the whole-cell voltage-clamp configuration. Cortical laminae were defined based on visible boundaries formed by differential cell densities in the brightfield image of the slice (Weiler et al., 2008; Hooks et al., 2011) and reported as the normalized distance of the cells between pia and white matter. L5A is the pale band in the brightfield image above the more heavily myelinated L5B (Yu et al., 2008). L2/3 cells were within 8–38% and L5A cells were within 20–58% of the slice thickness, depending on the curvature and anterior-posterior position of the slice (Fig. 3*C*). Our prior data, using subcellular Channelrhodopsin-assisted circuit mapping (sCRACM), had shown that both vS1 input and PO input similarly excited vM1 L5A Pyr neurons most strongly and L2/3 Pyr neurons with ∼70–90% of this strength (Mao et al., 2011; Hooks et al., 2013). But for PV+ neurons, this pattern shifted and vS1 excited L2/3 PV+ neurons more strongly than L5A, while PO excited L5A PV+ neurons more strongly than L2/3 (Okoro et al., 2022). Based on this difference in connection strength, EPSCs amplitudes in PV+ cells divided by the amplitudes of EPSCs in Pyr cells should result in larger ratios from vS1 stimulation compared with PO stimulation in L2/3 in our paired recordings. Consistent results confirm that the wide-field LED stimulation in the 2CRACM approach produces similar results to those predicted from earlier circuit mapping approaches (which differs by the use of tetrodotoxin (TTX) to prevent action potentials in the slice and ensure monosynaptic responses; Petreanu et al., 2009). We grouped recordings by laminar position, focusing on pairs within L2/3 and L5A, where long-range input from the pathways studied is strongest.

EPSC amplitudes showed that vS1 inputs to PV+ cells are stronger compared with PO inputs in vM1 L2/3 (Wilcoxon signed-rank *p* = 5E-6, effect size η^2^ = 0.296, [95.0%CI 0.351, 0.85]; Fig. 3*D*), while comparisons in L5A showed similar synaptic strength (Fig. 3*D*,*G*). Comparisons of input strength to PV+ and Pyr neurons in the same layer generally revealed stronger amplitude EPSCs to PV+ neurons (Independent-Samples Mann–Whitney test, vS1 inputs to L2/3 PV+ compared with Pyr, total *N* = 161, *p* = 3E-6, η^2^ = 0.143; PO inputs to L2/3 PV+ compared with Pyr, total *N* = 143, *p* = 3.2E-2, η^2^ = 0.032; to L5A PV+ compared with Pyr, total *N* = 57, *p* = 2.88E-3, η^2^ = 0.159.) As predicted, the PV+ to Pyr input ratio (Fig. 3*G*) was greater for vS1 inputs than for PO inputs (Wilcoxon signed-rank, *p* = 1.3E-2, η^2^ = 0.212).

### Connectivity between PV+ and Pyr neuron pairs

**Figure 4.**
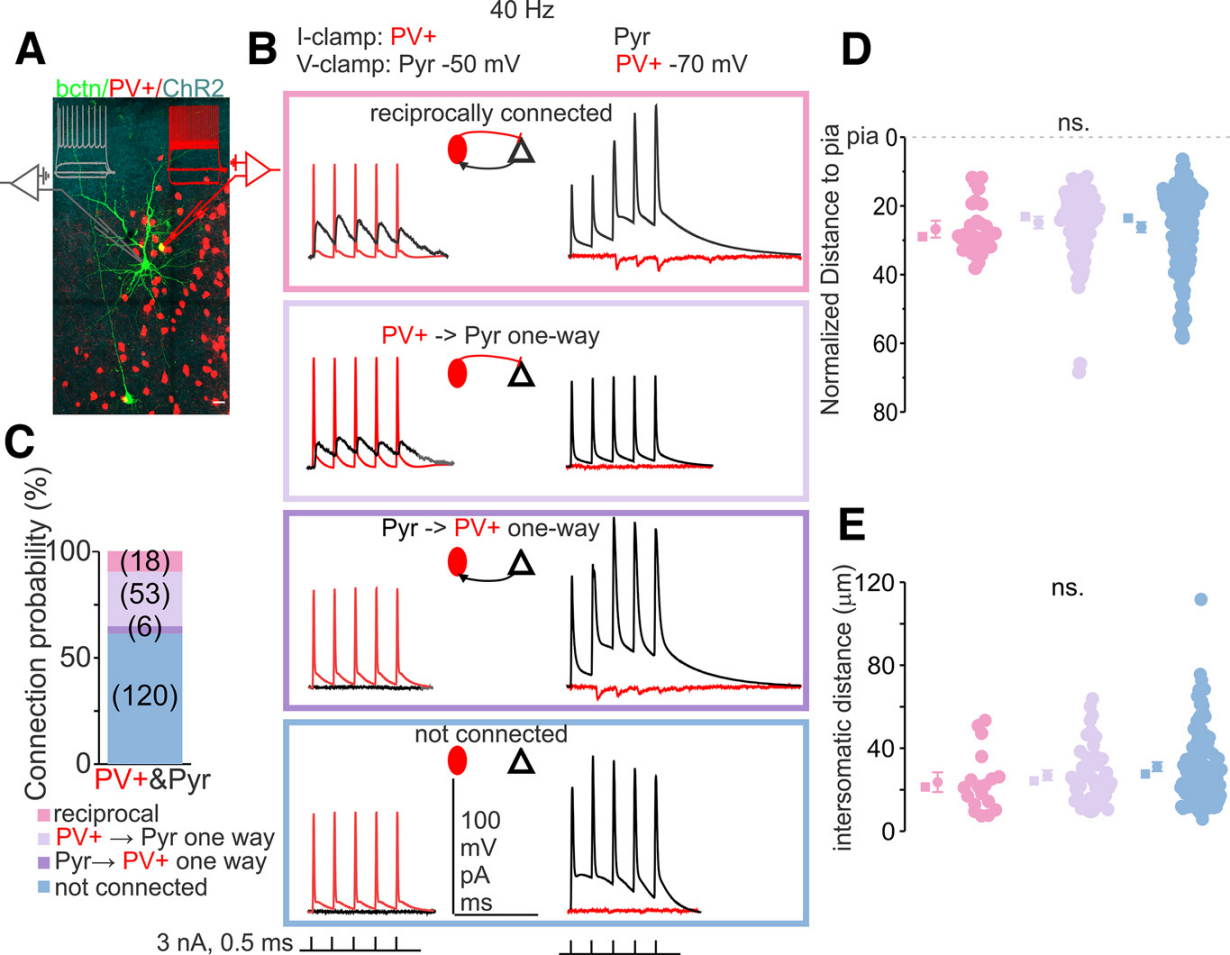
Characterization of connectivity between PV+ and Pyr neurons. ***A***, Example of biocytin filled 3 cells (green) and current-clamp traces show characteristic PV+ (red) and Pyr neurons subthreshold responses and suprathreshold AP firing in response to depolarizing current steps (PV+ red, Pyr gray) with ChR2 positive axons (cyan). One PV+ filled with biocytin is shown in yellow, because of overlap of tdtomato labeling. ***B***, Example of connection test traces for reciprocally connected (top), connected in one-way from PV+ to Pyr (second row), connected in one-way from Pyr to PV+ (3rd row), and nonconnected (fourth row). ***C***, Percentage of connected pairs (Fisher’s exact test 20.81, *p* = 1.6E-5; Extended Data Table 4-1). ***D***, Normalized distance to pia, the distance from pia to white matter is 100%, means (circles) and medians (squares) are to the left with whiskers showing ±95% confidence intervals. Because of nonhomogenous variance, Levene’s test *p* < 0.001, Welch test used for comparison of the means did not show any significant difference, *F*_(2,104.52)_ = 1.05, *p* = 0.36 with Games–Howell *post hoc* ns., between the reciprocally connected pairs (pink), unidirectionally connected pairs (powder blue) and nonconnected pairs (infant blue). ***E***, Euclidian distance between all the cell pairs were recorded within 120 μm of each other and show no difference, although the tendency of connected pairs having a smaller Euclidian intersomatic distance is showing (average Euclidian distance between reciprocally connected pairs = 23.65 μm, median = 21.29 μm, *n* = 18; between unidirectionally connected pairs avg = 26.96 μm, median = 24.22 μm, *n* = 58; between nonconnected pairs avg = 31.11 μm, median = 27.6 μm, *n* = 119, one-way ANOVA F = 2.52, *p* = 0.08 ns.; Extended Data Table 4-1). Scale bar for ***A*** is 50 μm.

Paired recordings allowed identification of Pyr and PV+ neuron pairs that were connected or unconnected, providing comparison between neurons in the same local network. Connectivity was tested bidirectionally between 197 PV+ and Pyr pairs (Fig. 4*A–C*). Of these, ∼39% were connected (*N* = 77/197), either unidirectionally or bidirectionally.

10.1523/ENEURO.0488-22.2023.tab4-1 Extended Data Table 4-1 Comparison of PV+ and Pyr neuron pair recording data across the literature. Data for all pairs. The data are dominated by intralaminar connections, since PV+ to Pyr, or Pyr to PV+ interlaminar connections are scarcer. Paired or multipaired: refers to whole-cell patch clamp of two or more neurons. Sharp refers to sharp electrode intracellular recording. 2P refers to two-photon stimulation and recording. ChR refers to optogenetic expression of light-sensitive actuator molecules (Channelrhodopsin variants), through viral vector or genetic crossing. Rubi-glut refers to Rubi-glutamate uncaging. Intersomatic distance is reported as a measure of Euclidian distance. Reported a horizontal offset if Euclidian distance was not calculated. In area column, S: somatosensory, V: visual, M: motor. Where no distinction between the interneuron types is made, both types are included in the percentage calculation. GC: granule cells. CThN: corticothalamic neurons. CCN: corticocortical neurons. Upper layer 6a is the top 40% of the layer height, lower layer 6a is the bottom 40% of the layer height. FS: fast-spiking interneurons, considered to be a part of PV+ cells; LTS- low-threshold spiking interneurons are considered to be a part of SOM+ cells. RS: regular-spiking neurons, mostly correspond to Pyr cells. Cg1/2: prefrontal cingulate cortex area 1/2 (dACC, dorsal anterior cingulate cortex). Depressing synapses are presumed to be from PV+ interneurons. Download Table 4-1, DOCX file.

This percentage falls within the broad range of previous reports on connectivity between those types of cells (Extended Data Table 4-1; Beierlein and Connors, 2002; Thomson and Morris, 2002; Beierlein et al., 2003; Holmgren et al., 2003; Gabernet et al., 2005; Yoshimura and Callaway, 2005; Kapfer et al., 2007; Gibson et al., 2008; Hofer et al., 2011; House et al., 2011; Packer and Yuste, 2011; Avermann et al., 2012; Jiang et al., 2015; Pala and Petersen, 2015; Guan et al., 2017; Espinoza et al., 2018; Gainey et al., 2018; Jouhanneau et al., 2018; Frandolig et al., 2019; Campagnola et al., 2022; Hage et al., 2022; for review, see Ali et al., 1999; Gupta et al., 2000; Thomson and Lamy, 2007). PV+ connectivity to Pyr neurons was present in 71 pairs (53 unidirectional and 18 bidirectional). Pyr connectivity to PV+ neurons was less frequent, occurring in only 24 pairs (6 unidirectional and 18 bidirectional). The relatively high connection probability from PV+ to Pyr neurons with reciprocal connectivity is striking (75%, 18 of 24, Fisher’s exact test 20.81, *p* = 1.6E-5 Odds Ratio = 7.059 two-sided tail *p* = 4.08E-05 [95.0%CI 2.65,18.81]; Yoshimura and Callaway, 2005). All the pairs were recorded within 120 μm of Euclidian distance apart (Fig. 4*E*). No significant difference was found within this distance between reciprocally connected, unidirectionally connected and nonconnected pairs (average Euclidian distance between bidirectionally connected pairs = 23.65 μm, median = 21.29 μm; unidirectionally connected pairs avg = 26.96 μm, median = 24.22 μm; nonconnected pairs avg = 31.11 μm, median = 27.6 μm; one-way ANOVA *F*_(2,192)_ = 2.52, *p* = 0.08, Tukey’s *post hoc* not significant; Fig. 4*E*). Additionally, since connection probability between recorded pairs decreased with increasing distance (Packer and Yuste, 2011; Hage et al., 2022), we compared Euclidian distance with one-tailed independent samples Student’s *t* test, because of smaller sample size for bidirectionally connected pairs (*n* = 18) and known predicted difference of larger distance between nonconnected pairs (*n* = 119). The difference between bidirectionally connected pairs to nonconnected pairs produced a statistically significant result (*t*_(135)_ = 1.74, *p* = 0.04). The recorded pairs did not differ in their distance to pia (because of nonhomogenous variance, Levene’s test *p* < 0.001, Welch test was used for comparison of the means did not show any significant difference, *F*_(2,104.52)_ = 1.045, *p* = 0.36 with Games–Howell *post hoc* not significant).

### Somatosensory cortical and thalamic excitation in connected versus nonconnected PV+ and Pyr neurons

**Figure 5.**
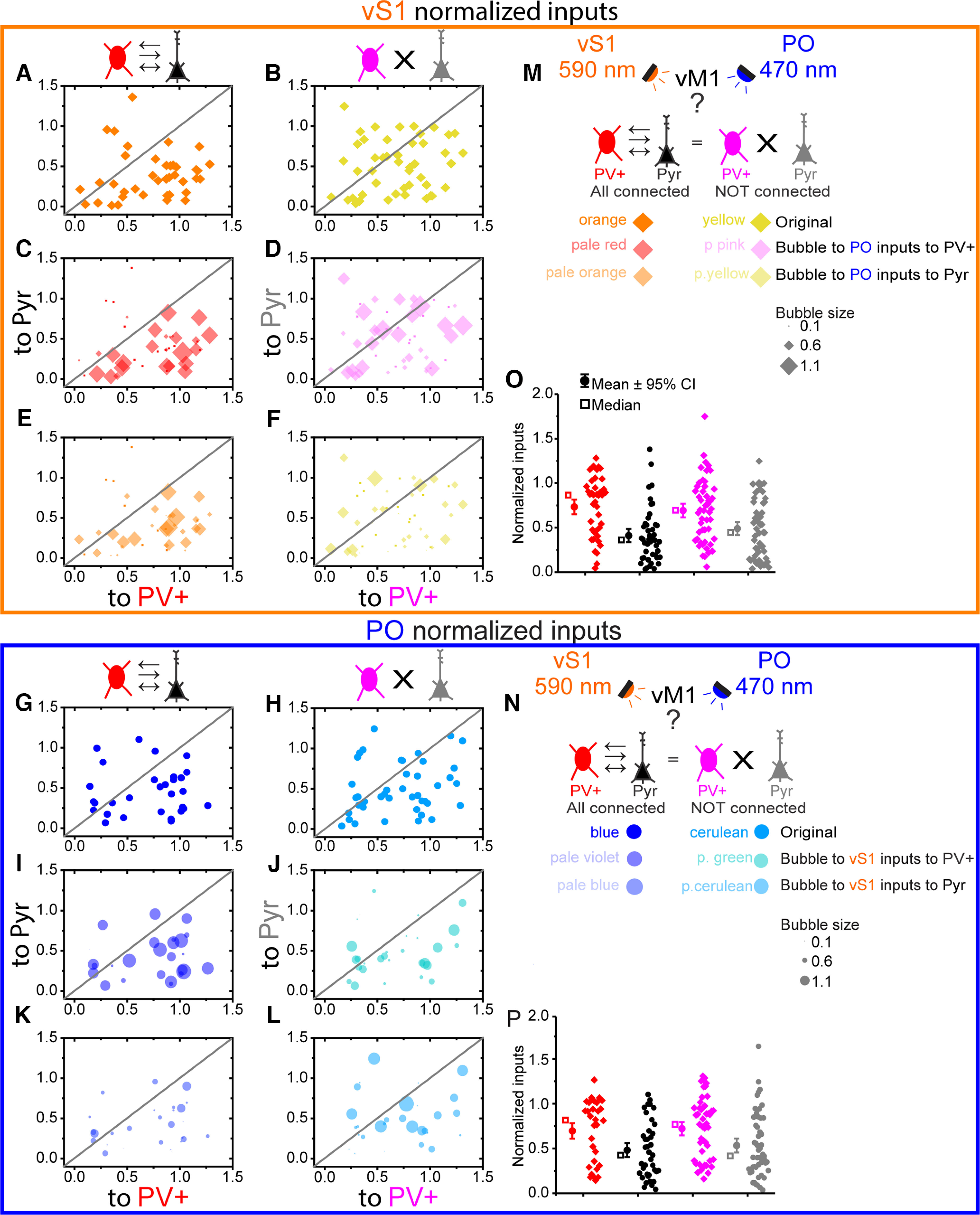
Normalized vS1 and PO inputs to connected and not pairs of PV+ and Pyr neurons show different trends. All inputs are from the 100-ms delay protocol normalized to the maximum slice peak at 250-ms delay protocol. ***A***, ***C***, ***E***, vS1 (orange) inputs for connected PV+ (red) and Pyr (black) pairs. ***A***, Original figure. ***C*** is ***A*** bubble plotted to PO (blue) inputs to PV+ neurons in the same pairs. ***E*** is ***A*** bubble plotted to PO inputs to Pyr in the same pairs. ***B***, ***D***, ***F***, vS1 inputs for nonconnected PV+ (magenta) and Pyr (gray) pairs. ***B***, Original figure. ***D*** is ***B*** bubble plotted to PO inputs to PV+ neurons in the same pairs. ***F*** is ***B*** bubble plotted to PO inputs to Pyr in the same pairs. ***G***, ***I***, ***K***, PO inputs for connected PV+ and Pyr pairs. ***G***, Original figure. ***I*** is ***G*** bubble plotted to vS1 inputs to PV+ neurons in the same pairs. ***K*** is ***G*** bubble plotted to vS1 inputs to Pyr neurons in the same pairs. ***H***, ***J***, ***L***, PO inputs for nonconnected PV+ and Pyr pairs. ***H***, Original figure. ***J*** is ***H*** bubble plotted to vS1 inputs to PV+ neurons in the same pairs. ***L*** is ***H*** bubble plotted to vS1 inputs to Pyr neurons in the same pairs. Bubble plots scale is from 0.1 to 1.6 with Δ of 0.5. ***M***, Schematics of scientific inquiry question, methods to study it, bubble plots color coding and size for vS1 inputs. ***N***, Schematics of scientific inquiry question, methods to study it, bubble plots color coding and size for PO inputs. ***O*** is ***A*** and ***B*** shown as a box plots. ***P*** is ***G*** and ***H*** shown as a box plots. Means are shown by circles, medians by squares and whiskers represent 95% confidence intervals.

To test whether excitation from somatosensory cortex and thalamus is organized differently in connected versus nonconnected pairs of PV+ and Pyr cells, we compared whole-cell voltage-clamp responses to vS1 and PO excitation in simultaneously recorded pairs of neurons. The similar distribution of ChR2-induced EPSC amplitude with ReaChR-induced EPSC amplitude (Fig. 3*D*) following the same amount of viral volume injected into PO and vS1 suggests both pathways excite vM1 with roughly similar strength. To compare the responses across different slices and animals and account for the difference in ChR2 and ReaChR expression levels between individual animals, opsin-mediated EPSCs amplitudes were normalized to the maximum response in the slice during the 250-ms delay sweep (Fig. 5). Only the slices with at least two cells were included. To visualize both somatosensory cortical and thalamic inputs in the same pairs in the same graphs, we used scatter bubble plots, that allows to show the third dimension by controlling the size of the data points. Thus, plots comparing vS1 input to PV+ and Pyr neurons could also show the strength of PO input with the size of the marker. Visual inspection shows a difference in normalized EPSCs amplitudes between connected and nonconnected pairs of PV+ and Pyr cells, which is emphasized when the thalamic inputs to PV+ or Pyr cells are chosen as a third dimension/variable (controlling the size of each data point in the pair receiving input from somatosensory cortex; Fig. 5). Specifically, PV+ neurons receive stronger vS1 inputs than nearby Pyr neurons (Fig. 5*A*,*B*), resulting in most points falling below the unity line (gray). Furthermore, the scatter of these points is reduced in connected versus nonconnected pairs (Fig. 5*A–F*), resulting in fewer points scattered above the line, suggesting less variance. This trend also seems to hold for PO inputs to PV+ and Pyr neurons (Fig. 5*G–L*), although less pronounced. The normalized input strength is comparable for PV+ and Pyr in both groups, yet the distribution is shifted (skewness for the VS1 normalized inputs in nonconnected PV+ = 0.454, in nonconnected Pyr = 0.329; in connected PV+ skewness = −0.353, in connected Pyr = 1.284; kurtosis in nonconnected PV+ = 0.089, in nonconnected Pyr = −1.097, in connected PV+ kurtosis = −0.916, in connected Pyr kurtosis = 1.798; skewness for the PO normalized inputs in nonconnected PV+ = −0.013, in nonconnected Pyr = 0.890, in connected PV+ skewness = −0.370, in connected Pyr = 0.474; kurtosis for the PO normalized inputs in nonconnected PV+ = −1.182, in nonconnected Pyr = 0.501, in connected PV+ kurtosis = −1.377, in connected Pyr = −1.012; Fig. 5*O*,*P*).

Normalized EPSCs showed that vS1 preferentially targets PV+ compared with Pyr neurons regardless of whether Pyr-PV+ pairs were connected, but connected pairs showed a bigger difference between the Pyr EPSCs (connected Pyr normalized mean = 0.41; nonconnected Pyr normalized mean = 0.49) and PV+ EPSCs (connected PV+ normalized mean = 0.73; nonconnected PV+ normalized mean = 0.69), mean difference (connected Δ = 0.32; nonconnected Δ = 0.2) with larger variance in nonconnected pairs (connected PV+ STDEV^2^ = 0.11, connected Pyr STDEV^2^ = 0.09; nonconnected PV+ STDEV^2^ = 0.13, nonconnected Pyr STDEV^2^ = 0.11; Fig. 5*O*). The PO inputs were larger in the same pairs with PV+ preference in connected cells, while large PO inputs were more broadly distributed in nonconnected cells (Fig. 5*C*,*D*).

**Figure 6.**
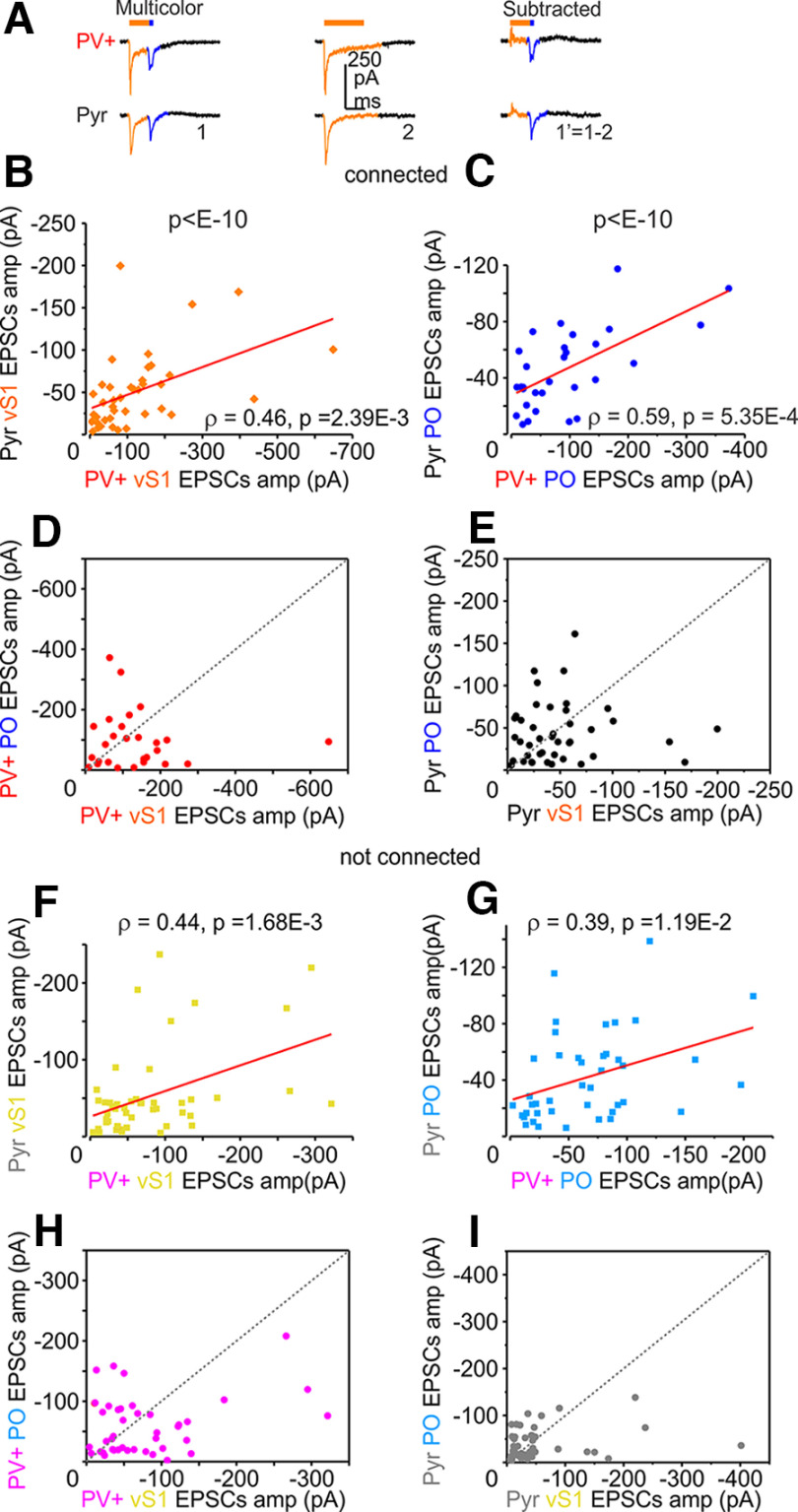
Increased correlation of the long-range inputs to connected pairs. ***A***, Example of voltage-clamp traces from PV+ (red) and Pyr pair with 590-nm stimulation of vS1 axons and 470-nm stimulation of PO (blue) axons middle traces are 590-nm stimulation alone and the right traces show the result of subtraction of middle traces from the first traces. Connected pairs are ***B–E***. ***B***, Scatter plot of vS1 in connected pairs showing a higher correlation compared with nonconnected pairs in ***F***, also see Extended Data Figures 6-2 and 6-3 for layer-specific information. The data in ***B*** and ***F*** were resampled using 1000 samples bootstrap and produced Spearman’s ρ correlation coefficients, which were squared and compared with nonparametric independent samples Mann–Whitney test, *U* = 208,790.0, *p* < 1E-10 (Extended Data Fig. 6-1). The estimation of confidence interval for the Spearman’s ρ correlation coefficient was also done with 10,000 Bootstrap resampling, [95.0%CI 0.324, 0.760; Ho et al. (2019)]. ***C***, Scatter plot of PO EPSCs in connected pairs showing a higher correlation compared with nonconnected pairs in ***G***. The data in ***C*** and ***G*** were resampled using 1000 samples bootstrap and produced Spearman’s ρ correlation coefficients, which were squared and compared with nonparametric independent samples Mann–Whitney test, *U* = 279,207.0, *p* < 1E-10 (Extended Data Fig. 6-1). The estimation of confidence interval for the Spearman’s ρ correlation coefficient was also done with 10,000 bootstrap resampling, [95.0%CI 0.163, 0.759]. ***D***, Scatter plot of PO and vS1 EPSCs in the PV+ interneurons from the connected pairs. ***E***, Scatter plot of PO and vS1 EPSCs in the Pyr neurons from connected pairs. Nonconnected pairs are ***F–I***. ***F***, Scatter plot of vS1 EPSCs from nonconnected pairs (yellow), 10,000 bootstrap resampling, [95.0%CI 0.097, 0.623]. ***G***, Scatter plot of PO (teal) EPSCs in nonconnected pairs, 10,000 bootstrap resampling, [95.0%CI 0.165, 0.658]. ***H***, Scatter plot of PO and vS1 EPSCs in the PV+ (magenta) interneurons from the nonconnected pairs. ***I***, Scatter plot of PO and vS1 EPSCs in the Pyr neurons from the nonconnected pairs. Correlations are estimated by the Spearman’s ρ correlation coefficient.

We then sought to test whether the connected cell pairs had correlated inputs. We speculated that interconnected subnetworks of neurons performing similar computations might get correlated input, such as both PV+ and Pyr neurons receiving strong or weak input. The alternative is that long-range input strength would be random with respect to whether pairs were connected. We plotted the EPSC amplitudes for connected and nonconnected pairs (Fig. 6*B*,*C*,*F*,*G*). We then fit these with a linear regression, finding that EPSCs from both vS1 and PO inputs before normalization showed higher correlation in connected (vS1 inputs in connected pairs Spearman ρ = 0.46, *p* = 2.39E-3, confidence interval of [95.0%CI 0.324, 0.760]; PO inputs in connected pairs, ρ = 0.59, *p* = 5.35E-4, [95.0%CI 0.163, 0.759]) compared with nonconnected pairs (vS1 inputs in nonconnected pairs, ρ = 0.44, *p* = 1.68E-3, [95.0%CI 0.097, 0.623]; PO inputs in nonconnected pairs, ρ = 0.39, *p* = 1.19E-2, [95.0%CI 0.165, 0.658]; Fig. 6; Extended Data Figs. 6-1, 6-2, 6-3, the data were resampled 1000 times bootstrapped and produced correlation coefficients that were compared between those groups, *p* < E-10) suggesting that co-targeting of long-range projections is dependent on whether they contact connected or nonconnected pairs.

10.1523/ENEURO.0488-22.2023.f6-1 Extended Data Figure 6-1 Statistical comparison of bootstrapped vS1 and PO inputs correlation coefficients. ***A***, Violin plots of the resampled vS1 data Spearman’s ρ^2^ correlation coefficients compared between connected and not connected pairs. The estimation of confidence interval for the bootstrapped Spearman’s ρ correlation coefficients was done with 10,000 bootstrap resampling, for VS1 inputs to connected pairs [95.0%CI 0.114, 0.711]; for VS1 inputs to not connected pairs [95.0%CI 0.072, 0.668]; the effect size = 0.242 (η^2^), is calculated based on Mann–Whitney *U* test comparison of connected versus not connected pairs. ***B***, Violin plots of the resampled PO data Spearman’s ρ^2^ correlation coefficients compared between connected and not connected pairs. The estimation of confidence interval for the bootstrapped Spearman’s ρ correlation coefficients was done with 10,000 bootstrap resampling, for PO inputs to connected pairs [95.0%CI 0.135, 0.664]; for PO inputs to not connected pairs [95.0%CI 0.070, 0.658]; the effect size = 0.136 (η^2^), is calculated based on Mann–Whitney *U* test comparison of connected versus not connected pairs. Download Figure 6-1, TIF file.

10.1523/ENEURO.0488-22.2023.f6-2 Extended Data Figure 6-2 Long-range inputs have a layer-specific differential correlation for connected pairs. ***A***, Scatterplot of vS1 EPSCs in connected pairs in layer 2/3. The estimation of confidence interval for the Spearman’s ρ correlation coefficients was done with 10,000 bootstrap resampling, [95.0%CI 0.291, 0.777]. ***B***, Scatter plot of PO EPSCs in connected pairs in layer 2/3, [95.0%CI 0.064, 0.792]. ***C***, Scatter plot of vS1 EPSCs in connected pairs between layer 2/3 and 5A, [95.0%CI −1, 1]. ***D***, Scatter plot of PO EPSCs in connected pairs between layer 2/3 and 5A, [95.0%CI −1, 1]. Download Figure 6-2, TIF file.

10.1523/ENEURO.0488-22.2023.f6-3 Extended Data Figure 6-3 Long-range inputs have a layer-specific differential correlation for nonconnected pairs. ***A***, Scatter plot of vS1 EPSCs in nonconnected pairs in layer 2/3. The estimation of confidence interval for the Spearman’s ρ correlation coefficients was done with 10,000 bootstrap resampling, [95.0%CI 0.048, 0.720]. ***B***, Scatterplot of PO EPSCs in nonconnected pairs in layer 5A, [95.0%CI 0.139, 0.757]. ***C***, Scatterplot of vS1 EPSCs in nonconnected pairs in layer 5A, [95.0%CI −0.406, 0.759]. ***D***, Scatterplot of PO EPSCs in nonconnected pairs in layer 5A, [95.0%CI −0.244, 0.800]. Download Figure 6-3, TIF file.

### Excitation-to-inhibition ratio of vS1 and PO inputs

**Figure 7.**
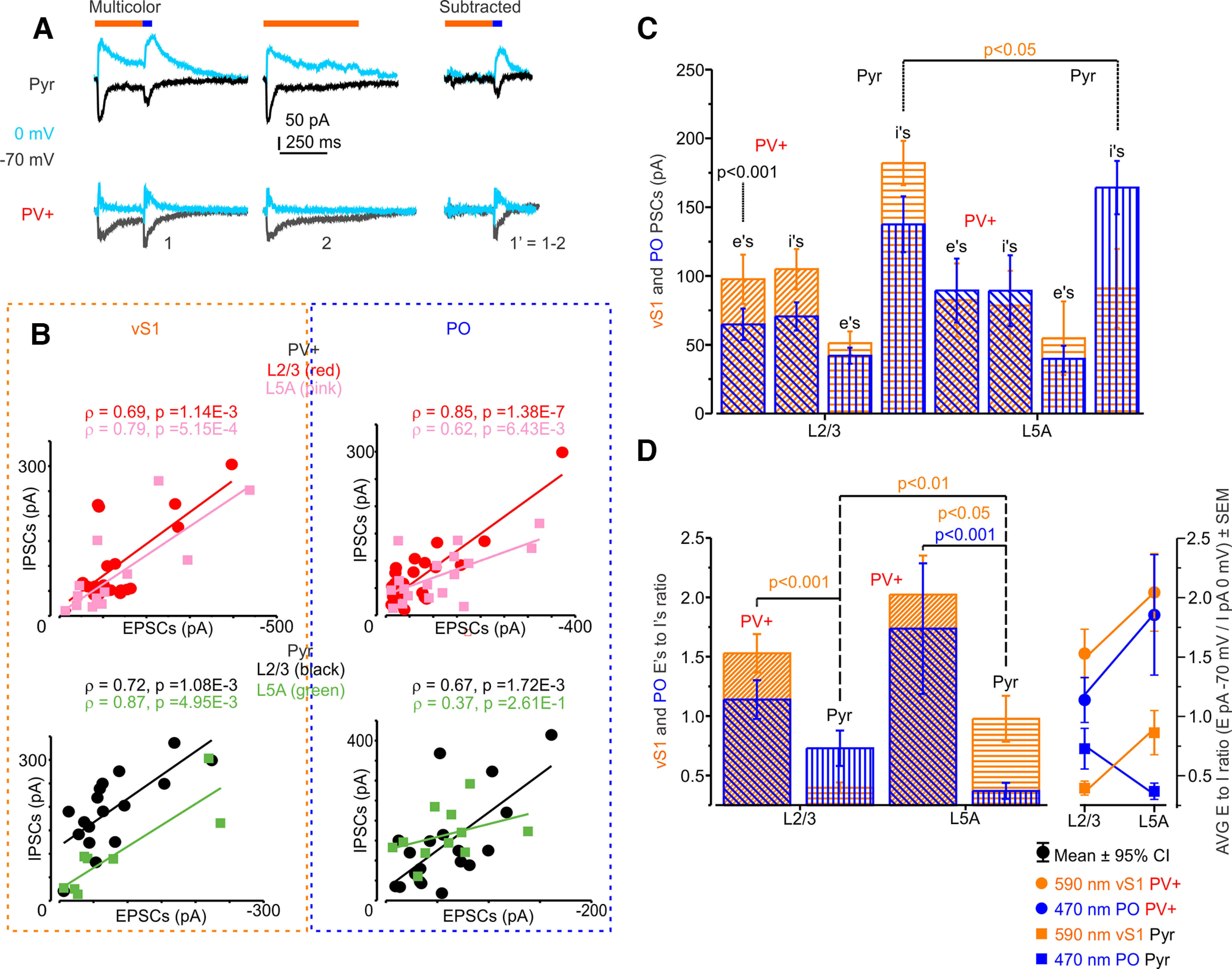
Long-range inputs excite more PV+ interneurons eliciting stronger feedforward inhibition in layer-specific manner. ***A***, Example traces showing a pair of PV+ and Pyr neurons recorded at −70 mV and at 0 mV (teal) holding potential. ***B***, Scatter plot of EPSCs and IPSCs for vS1 inputs (left panel), and PO (right panel) for PV+ (upper panel) and Pyr (lower panel). Cortical layers 2/3 data for PV+ (red, 10,000 bootstrap confidence interval for Spearman’s ρ for VS1 inputs [95.0%CI −0.143, 0.770]; for PO inputs [95.0%CI 0.155, 0.824]) and L5A (pink, for VS1 inputs [95.0%CI 0.176, 0.918]; for PO inputs [95.0%CI −0.134, 0.839]) for Pyr the L2/3 data are in black (for VS1 inputs [95.0%CI 0.359, 0.913]; for PO inputs [95.0%CI 0.109, 0.847]) and L5A is in green (for VS1 inputs [95.0%CI 0.556, 1]; for PO inputs [95.0%CI 0.166, 0.832]). ***C***, vS1 and PO EPSCs (e’s) at −70 mV holding potential multiplied by −1 for the convenience of presentation, and IPSCs (I’s) at 0 mV holding potential; EPSCs in L2/3 PV+ neurons were significantly larger from vS1 than PO (Wilcoxon signed-rank test = 2131, *p* = 5E-6, effect size η^2^ = Z^2^/(n−1), η^2^ = 0.296; Fig. 3*D*). vS1 Is were significantly larger in L2/3 than L5A Pyr [independent samples Mann–Whitney (M–W) *U* = 39, *p* = 3.08E-2, η^2^ = 0.180]. ***D***, Averaged layer-specific excitation-to-inhibition ratio where the amplitude of EPSCs at −70 mV is divided by the amplitude of IPSCs at 0 mV for each cell. E/I ratio for vS1 inputs was significantly larger in PV+ than in Pyr in L2/3 but not for PO inputs [M–W *U* = 52 and 186.5 (PO), *p* = 2.2E-5 and 9.37E-2 (PO), η^2^ = 0.409 and η^2^ = 0.062 (PO)]; vS1 inputs E/I ratio was significantly larger in L5A than L2/3 Pyr (M–W *U* = 182, *p* = 4.89E-3, η^2^ = 0.254); vS1 and PO inputs E/I ratio was significantly larger in L5A PV+ than Pyr [M–W *U* = 49 and 41.5 (PO), *p* = 1.83E-2 and 4.0E-4 (PO), η^2^ = 0.197 and η^2^ = 0.344 (PO)]. Correlation is estimated by the Spearman’s ρ correlation coefficient.

**Figure 8.**
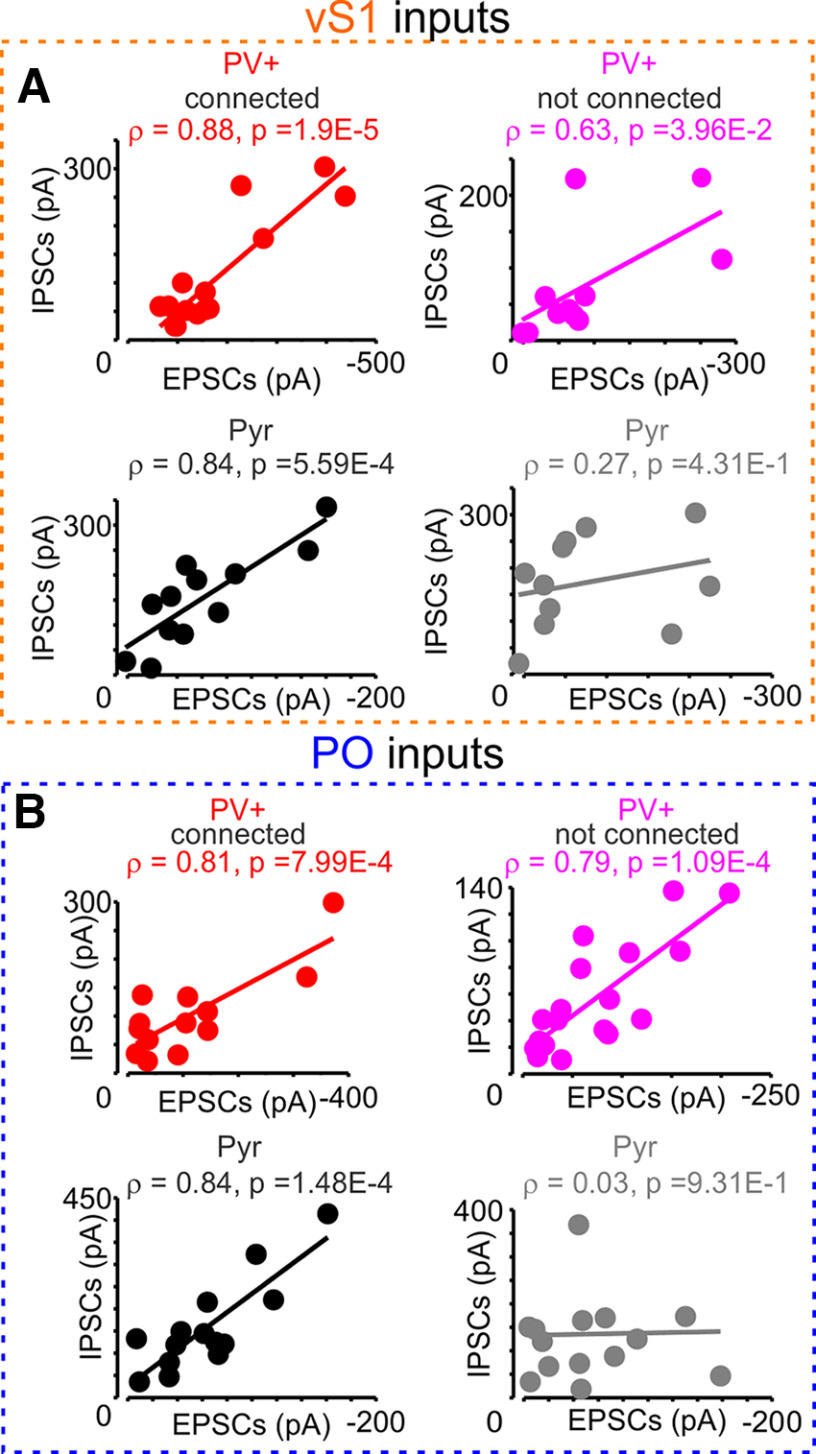
Correlation of long-range excitatory and feedforward inhibitory inputs is weaker in nonconnected Pyr cells. ***A***, Scatter plots of vS1 (orange), and (***B***) PO (blue) inputs EPSCs (*x*-axis) and IPSCs (*y*-axis) for connected (left panels) PV+ (red) and Pyr (black) and nonconnected (right panels, magenta for PV+ and gray for Pyr). The estimation of confidence interval for the Spearman’s ρ correlation coefficient was also done with 10,000 bootstrap resampling, for VS1 inputs to PV+ connected (***A***, upper left), [95.0%CI 0.033, 0.888]; for VS1 inputs to PV+ not connected (***A***, upper right), [95.0%CI 0.090, 0.962]; for VS1 inputs to Pyr connected (***A***, lower left), [95.0%CI 0.394, 0.978]; for VS1 inputs to Pyr not connected (***A***, lower right), [95.0%CI −0.377, 0.942]; for PO inputs to PV+ connected (***B***, upper left), [95.0%CI −0.062, 0.904]; for PO inputs to PV+ not connected (***B***, upper right), [95.0%CI 0.465, 0.917]; for PO inputs to Pyr connected (***B***, lower left), [95.0%CI 0.093, 0.947]; for PO inputs to Pyr not connected (***B***, lower right), [95.0%CI −0.405, 0.640].

We wanted to test how the recruitment of PV+ cells by long-range projections is correlated with the ReaChR and ChR2-induced IPSCs in both PV+ and Pyr cells. As before, we thought that interconnected subnetworks of neurons might get correlated excitatory and inhibitory input. Thus, we voltage clamped the pairs at 0 mV after acquiring the excitatory responses. For both PO and vS1 inputs, opsin-induced IPSCs correlation with EPSCs in L2/3 and L5A PV+ and Pyr cells was assessed with Spearman’s ρ correlation coefficient. Correlation of vS1-evoked IPSCs to EPSCs in L2/3 PV+ neurons was ρ = 0.69, *p* = 1.14e-3, [95.0%CI −0.143, 0.770] (Fig. 7*B*). In L5A PV+ neurons, it was ρ = 0.79, *p* = 5.15e-4, [95.0%CI 0.176, 0.918]. For L2/3 Pyr cells, vS1-evoked IPSCs to EPSCs correlation was ρ = 0.72, *p* = 1.08e-3, [95.0%CI 0.359, 0.913], and, in L5A Pyr cells, it was ρ = 0.87, *p* = 4.95e-3, [95.0%CI 0.556, 1]. The PO-evoked IPSCs to EPSCs correlation in L2/3 PV+ cells was ρ = 0.85, *p* = 1.38e-7, [95.0%CI 0.155, 0.824], and, in L5A PV+ cells, it was ρ = 0.62, *p* = 6.43e-3, [95.0%CI −0.134, 0.839]. PO-evoked IPSCs to EPSCs correlation in L2/3 Pyr cells was ρ = 0.67, *p* = 1.72e-3, [95.0%CI 0.109, 0.847], with Pyr cells having less correlation from PO inputs in L5A (ρ = 0.37, *p* = 2.61e-1, [95.0%CI 0.166, 0.832]). L2/3 Pyr cells also had an increased IPSCs amplitudes in L2/3 from vS1 inputs (Fig. 7*B*,*C*). There was also less correlation of IPSCs to EPSCs in nonconnected Pyr cells from both vS1 and PO inputs (nonconnected Pyr vS1 EPSCs to IPSCs ρ = 0.27, *p* = 4.31e-1, [95.0%CI −0.377, 0.942]; nonconnected Pyr PO EPSCs to IPSCs ρ = 0.03, *p* = 9.31e-1, [95.0%CI 0.465, 0.917]; Fig. 8*A*,*B*).

Comparison of excitatory to inhibitory response ratio within the same cells between L2/3 and L5A showed that for Pyr cells the vS1 excitatory drive was larger in L5A compared with L2/3 (E/I ratio, Mann–Whitney, *p* = 4.89E-3, η^2^ = 0.254; Fig. 7*C*,*D*) confirming the results from a previous study (Mao et al., 2011). For both L2/3 and L5A, the vS1 excitatory drive was larger for PV+ cells than for Pyr cells, while PO excitatory drive was significantly larger only in L5A PV+ cells compared with L5A Pyr cells (L2/3 vS1 inputs E/I ratio Mann–Whitney, *p* = 2.2E-5, η^2^ = 0.409; L5A vS1 inputs E/I ratio Mann–Whitney, *p* = 1.83E-2, η^2^ = 0.197; L5A PO inputs E/I ratio Mann–Whitney, *p* = 4.0E-4, η^2^ = 0.344; Fig. 7*C*,*D*). Consistent with larger vS1 inputs in L2/3 PV+ cells the vS1 inhibitory responses were larger in L2/3 Pyr cells compared with L5A Pyr cells (Mann–Whitney, *p* = 3.08E-2, η^2^ = 0.180; Fig. 7*B–D*). This suggests that long-range inputs excite more PV+ cells eliciting stronger feedforward inhibition in a layer-specific manner.

## Discussion

Here, we tested whether locally connected subnetworks of Pyr and PV+ neurons exist in mouse M1 and whether these local networks differed in input from two major sources of long-range excitation. The data show that connected Pyr and PV+ neuron pairs indeed share correlated long-range input. Furthermore, Pyr neuron connectivity is elevated to PV+ neurons that inhibit them. This data collectively suggests that inhibitory neurons in motor cortex are specifically connected in local subnetworks.

### Excitatory subnetworks in motor cortex

Excitatory subnetworks are a set of connected Pyr cells receiving shared interlaminar (excitatory), intralaminar (inhibitory and excitatory) or long-range inputs (Yoshimura et al., 2005; Morgenstern et al., 2016), or sharing a single inhibitory cell as a hub (Yoshimura and Callaway, 2005; Palagina et al., 2019). Local connectivity supports the existence of such excitatory subnetworks within small anatomic loci (Yoshimura et al., 2005; W.A. Lee et al., 2016; Vegué et al., 2017; Faber et al., 2019; Palagina et al., 2019). Further, subnetworks might share common functional properties, such as receptive fields in visual areas (Ohki et al., 2005, 2006; Ko et al., 2011).

Motor cortex is somatotopically organized. Thus, M1 might contain distinct subnetworks for different motor functions, such as different movements of a given limb. Thus, we tested the local and long-range connectivity of PV+ and Pyr neurons to assess the existence of specific subnetworks. Connected pairs represented within-network neurons, and unconnected pairs were chosen to represent different networks. Here, we show that connected pairs of PV+ and Pyr cells in M1 have stronger correlation of long-range somatosensory (vS1) and thalamic (PO) projections than nonconnected pairs (Fig. 6). Thus, subnetworks with strong vS1 input exist and more strongly excite connected PV+ and Pyr neurons.

### Integration of interneurons into local subnetworks

It is controversial the degree to which interneurons participate in such subnetworks. Imaging interneuron response properties in visual cortex suggests that these cells are more broadly tuned than excitatory neurons (Kerlin et al., 2010), while other studies suggest PV+ cells may be selective to orientation and direction (Runyan et al., 2010). Further, in direction-selective or orientation-selective inhibitory interneurons (52%) and their clusters, 75% of clustered Pyr cells shared direction tuning with their corresponding inhibitory neuron (Palagina et al., 2019). Relatively dense local connectivity of PV+ neurons has been proposed, with both nonspecific but frequent output to local Pyr neurons (Packer and Yuste, 2011; Fino et al., 2013) and pooled excitatory input from Pyr cells with different properties (Sohya et al., 2007).

How to reconcile a role for selective inhibitory connectivity with nonselective all-to-all inhibition remains to be answered. One possibility is that connections are common, but synaptic strength selectively varies within or across subnetworks (Znamenskiy et al., 2018). One statistical/structural explanation for subnetworks is the targeting by feedforward and feedback long-range projections, which may increase the information propagation and processing in cortical circuits by selecting clustered cells that have higher probability of connecting to each other (Nigam et al., 2016; Rost et al., 2018; Faber et al., 2019; Palagina et al., 2019; Peron et al., 2020). Thus, synaptic inputs from long-range projections may be correlated in connected clusters.

In our data, selectively connected PV+ interneurons share correlated input strength with in-network pyramidal neurons. Furthermore, the contribution of interneurons to subnetworks is not simply correlated connection strength, but also enhanced connection probability. While PV+ neurons make frequent local connections to Pyr neurons (*N* = 71/197 pairs, 36.0%), Pyr neurons make sparser connections to PV+ cells (*N* = 24/197, 12.2%). However, Pyr neurons are much more likely to excite PV+ cells that reciprocally connect to them, with connection rates as high as those for PV+ output (*N* = 18/53, 34.0%). Thus, connected pairs were reciprocally connected at much higher than random rate (Fig. 4). This arrangement is consistent with a given Pyr neuron activating its own feedback inhibition, providing a negative feedback mechanism to stabilize excitability. The overall connection probability is potentially underestimated, as some connections may be severed in the brain slices. But it is unlikely that slice preparation differentially severs connections between neurons that lack correlated excitatory input.

### Thalamocortical and corticocortical connection strength in M1

These data show that, consistent with earlier work (Hooks et al., 2015), single Pyr and PV+ neurons receive input from both cortical (vS1) and thalamic (PO) sources. In assessing strength of connections, vS1 inputs were stronger in L2/3 PV+ cells than PO inputs (Fig. 3). Furthermore, both classes of excitatory inputs were much stronger in PV+ cells than to Pyr cells (Figs. 3, 5). This is in line with previous studies showing increased thalamocortical inputs to PV+ compared with Pyr cells in somatosensory cortex (Gibson et al., 1999; Gabernet et al., 2005; Cruikshank et al., 2007, 2010). There may be differences in measured EPSC strength because of differences between the cell types. PV+ cells may be more electrotonically compact, making larger inputs easier to measure, and differences in intrinsic excitability may favor higher firing rates in PV+ neurons (Extended Data Fig. 1-1). These differences amplify the effectiveness of inputs in PV+ neurons. Thus, in general, the E/I ratio of PV+ cells is higher than the E/I ratio of Pyr cells (Fig. 7). Further, comparing input strength across layers (Fig. 7*D*), E/I ratio for vS1 input to Pyr cells increases from L2/3 to L5A implicating greater recruitment of feedforward inhibition in L2/3, presumed to originate from PV+ cells. In contrast, E/I ratio for thalamic input to Pyr cells goes down from L2/3 to L5A, implicating greater recruitment of feedforward inhibition in L5A (Okoro et al., 2022).

### Technical notes

This current work uses long optogenetic stimulations (50–500 ms), which may result in polysynaptic, recurrent activity in the vM1 circuits. Some opsin evoked EPSCs do show multiple peaks (Fig. 2). However, comparing sweeps with a range of stimulus durations (50, 100, 250, and 500 ms; Fig. 1*J*,*K*) suggests that longer duration stimuli do not activate increased polysynaptic responses, as the initial EPSC is of similar amplitude for different durations of 590-nm stimulation. There is a small possibility that the stimuli may activate the same small set of recurrent synapses in all durations protocols, in which case these recurrent synaptic inputs still would be considered to originate within the subnetwork by definition (clusters of connected cells), and at the same time it will need to produce input amplitudes bigger than the opsin evoked EPSCs to contribute increased variance to the analysis of current results. Further, previous work in our lab using subcellular CRACM, with application of TTX to block non-Channelrhodopsin evoked activity can produce similar multiple peak responses following short 1 ms, localized laser stimulation (Okoro et al., 2022). Comparison of opsin-evoked EPSCs onset latencies between sCRACM from our previous work and 2CRACM in current work also suggest minimal contribution of recurrent synaptic activity despite longer stimuli and EPSCs poly-peaks recordings (data not shown). It is also worth noting that a similar laminar pattern of input strength occurs following LED stimulation (this work) as is measured in TTX following laser stimulation (Okoro et al., 2022). Coupled with the low spontaneous AP firing rate in the cortex (for review, see Barth and Poulet, 2012), polysynaptic responses on wide-field LED stimulation are not expected to be major contributors to these results.

In conclusion, this work proposes a role for long-range projections as part of the neural circuit organization that differentiates the primary whisker motor cortex into subnetworks, to some degree as in visual and somatosensory cortices. Differences in cortical and thalamic input to different local subnetworks can result in local circuit elements specialized for processing different streams of information. However, these experiments are done *ex vivo* in mouse cortical slices, which may underestimate connectivity. Whether similar results can be obtained *in vivo* remains to be tested.

## Synthesis

Reviewing Editor: Niraj Desai, National Institute of Neurological Disorders and Stroke Decisions are customarily a result of the Reviewing Editor and the peer reviewers coming together and discussing their recommendations until a consensus is reached. When revisions are invited, a fact-based synthesis statement explaining their decision and outlining what is needed to prepare a revision will be listed below. The following reviewer(s) agreed to reveal their identity: NONE. Note: If this manuscript was transferred from JNeurosci and a decision was made to accept the manuscript without peer review, a brief statement to this effect will instead be what is listed below.

The manuscript is well-written, the experiments are thoughtfully designed, and the conclusions are interesting. Thank you for sumitting the manuscript to eNeuro.

One reviewer offered some cogent criticisms/suggestions (see below). Please respond to these individually.

REVIEW COMMENTS

The manuscript aims at investigating whether subnetworks made of excitatory and inhibitory neurons exist in the whisker region of mouse motor cortex (vM1), hypothesizing that they could serve as distinct functional modules involved in the control of movement. The authors quantify local connectivity between pairs of excitatory (Pyr) and PV-expressing FS neurons in L2/3 and L5a of vM1 and correlate it with the organization of long-range excitatory inputs originating in the whisker region of primary somatosensory cortex (vS1) and in the posterior region of the thalamus (PO). The authors find that there is a high rate of reciprocal connectivity between Pyr and PV neurons in vM1, and that connected pairs tend to receive more correlated excitatory inputs from vS1 and from PO than non-connected pairs.

The current work builds on previous work from the PI and his group validating the dual color optogenetic stimulation method.

It extends work from other groups on the existence of cortical excitatory subnetworks defined by their shared long-range inputs to subnetworks made of excitatory and inhibitory neurons. The manuscript is clearly written, with ample details to understand the authors reasoning, experiments, analyses, and interpretations. No additional experiments are required for publication, but a few points require clarification.

Major comments:

- Why use four different delays between the 590 nm and 470 nm LED stimuli throughout the manuscript? Although the discussion addresses polysynaptic connectivity related to long light stimuli, it is not clear why all four delays need to be carried out throughout all the experiments. It would be best to explain this upfront, either in the methods or in the results text related to Figure 1.

- Figure 4: does the dataset only contain pairs where the pre- and post-synaptic neurons are within the same layer or are across-layers pairs also included?

- Pala and Petersen 2015, the first paper to extensively characterize connectivity between Pyr and PV neurons in vivo seems to be missing from the thorough review of the literature summarized in Supp Table 2. That paper also investigated whether a relationship existed between connectivity rate and Euclidean distance between the cell somas, without finding a significant correlation within 120 um.

- Figure 5: some statistical analyses seem to be missing to support the claims of shifted distributions yet similar normalized input strengths.

- Figure 6: did some slices exhibit only connected or only non-connected pairs? If so, were the data coming from such slices included in the figure? Although unlikely it may be useful to clarify as it could affect the results.

- Figure 6: the correlation coefficient that is reported in the figure is the Pearson’s R yet bootstrapping to evaluate whether the R’s from the connected and non-connected groups are different is done on Spearman’s rho (figure legend and Supp Figure 7). Using R vs rho may not affect the results but considering that the data from this figure are central to the main finding and claim of the paper it would be better to be consistent or to justify the use of both R and rho.

Minor comments

- No mention of a plan to share the data, only of the acquisition and analysis software.

- Figure 2: comparisons between the PO and vS1-triggered EPSC properties could be made easier if the y-axes would span the same range of values (for instance B and C would go from 0 to 15 ms).

- Figure 6, panel A: are the example traces from a connected or a non-connected pair?

- Text from lines 376 to 387 seems misplaced. It would be better located starting at line 363, after the rest of the text describing Figure 5.

-Typos:

o Line 247: “ChannelRhodopsin”.

o Legend of Figure 3: images in panel C are referred to using “top right” and “lower”, it should probably be “middle” and “right”.

o Line 522: “is still remains”.

o Lines 720-723: Morgenstern reference is duplicated.

## Author Response

Synthesis of Reviews:

Synthesis Statement for Author (Required):

The manuscript is well-written, the experiments are thoughtfully designed, and the conclusions are interesting. Thank you for sumitting the manuscript to eNeuro.

One reviewer offered some cogent criticisms/suggestions (see below). Please respond to these individually.

REVIEW COMMENTS

The manuscript aims at investigating whether subnetworks made of excitatory and inhibitory neurons exist in the whisker region of mouse motor cortex (vM1), hypothesizing that they could serve as distinct functional modules involved in the control of movement. The authors quantify local connectivity between pairs of excitatory (Pyr) and PV-expressing FS neurons in L2/3 and L5a of vM1 and correlate it with the organization of long-range excitatory inputs originating in the whisker region of primary somatosensory cortex (vS1) and in the posterior region of the thalamus (PO). The authors find that there is a high rate of reciprocal connectivity between Pyr and PV neurons in vM1, and that connected pairs tend to receive more correlated excitatory inputs from vS1 and from PO than non-connected pairs. The current work builds on previous work from the PI and his group validating the dual color optogenetic stimulation method.

It extends work from other groups on the existence of cortical excitatory subnetworks defined by their shared long-range inputs to subnetworks made of excitatory and inhibitory neurons. The manuscript is clearly written, with ample details to understand the authors reasoning, experiments, analyses, and interpretations. No additional experiments are required for publication, but a few points require clarification.

Major comments:

- Why use four different delays between the 590 nm and 470 nm LED stimuli throughout the manuscript? Although the discussion addresses polysynaptic connectivity related to long light stimuli, it is not clear why all four delays need to be carried out throughout all the experiments. It would be best to explain this upfront, either in the methods or in the results text related to Figure 1.

Thank you for the opportunity to clarify the experimental design. We have added explanation in the methods section lines 197-206. These now read: “The four different delay protocols (50-500 ms) were used as a control for the effects of activation with 2 different wavelengths (590 nm and 470 nm) on the glutamate release caused by activation of red-shifted ReaChR channels first and the blue shifted ChR2 second. If the first stimulus (590 nm) does not affect the features of the second response, then we are confident that our paradigm is independently stimulating the two pathways.” Figure 2 has been changed to show only 100 ms and 250 ms delay protocols kinetics to conform to the rest of the analyzed and presented figures. Supplementary Figure 2 and 5 Supplementary Figure 3 have been removed and in text references has been corrected for all other supplementary figures (previous 4-7, current 2-5). All the supplementary material has been changed to the extended material with appropriate Figures and Tables names that they extend.

- Figure 4: does the dataset only contain pairs where the pre- and post-synaptic neurons are within the same layer or are across-layers pairs also included? Thank you for the question. In Figure 4, all pairs were included. Out of 197 pairs, we had 30 pairs that were estimated (based on the relative distance from pia and white matter) as interlaminar pairs, mostly involving L2/3 and L5A, with 14 being connected either reciprocally or unidirectionally and 16 not connected. That estimation includes reciprocally connected pairs (5: L2/3 to L5A - 3, L5A to L5B - 1, L5B to L2/3 - 1), unidirectionally connected pairs (PV to Pyr, 9: L5A to L2/3 - 4, L5B to L2/3 - 2, L2/3 to L5A - 3), and not connected pairs involving L2/3 and L5A using the same estimation of borders (16).

What this is asking about is the difference between inter vs intralaminar connectivity, because the connection probability may vary (potentially higher for intralaminar connections, and lower for interlaminar connections). In the literature, papers that did concentrate on connections probability used only intersomatic distance, including across laminar borders. Connections between Basket Cells (BC, PV+) and Pyramidal cells, for example, are estimated in Jiang et al., 2015 “Principles of connectivity among morphologically defined cell types in adult neocortex” in visual cortex. These show connectivity between L2/3BC and L5Pyr as well as L5BC to L2/3 Pyr. There are interlaminar connections between PV and Pyr cells in our dataset (∼14/30, roughly 46.7%), which is actually slightly higher than our overall dataset (∼77/197, roughly 39.1%). This certainly suggests that there is not a dramatic loss of connectivity in translaminar connections.

But because our translaminar sample is small and includes different combinations, this is not sufficient for a definitive answer that inter- and intralaminar connection probabilities differ.

-Pala and Petersen 2015, the first paper to extensively characterize connectivity between Pyr and PV neurons in vivo seems to be missing from the thorough review of the literature summarized in Supp Table 2 - changed to Table 4-1. That paper also investigated whether a relationship existed between connectivity rate and Euclidean distance between the cell somas, without finding a significant correlation within 120 um.

Thank you for reminding of this. Pala and Petersen (2015) does excellent work examining the connectivity between different cell types in vivo. I added the data of Pyr to PV+ connectivity in vivo derived from that paper to the Table 2. The citation is added in the appropriate places (Line 318 in the Results as well as in Supplementary Table 2 - changed to Table 4-1). Pala et al. 2015 (in-vivo 2P guided optogenetics in presynaptic Pyr to post Mouse S1, L2/3 P28-56 <125 μm 6 PV+ & SOM) Pyr→ PV+ 45 23 51 PV+ →Pyr Not tested PV+ ↔Pyr Not tested

-Figure 5: some statistical analyses seem to be missing to support the claims of shifted distributions yet similar normalized input strengths.

We have added the descriptive statistics (skewness and kurtosis) to the text lines 366-374: ”The normalized input strength is comparable for PV+ and Pyr in both groups, yet the distribution is shifted (Fig. 5O-P, skewness for the VS1 normalized inputs in non-connected PV+ = 0.454, in non-connected Pyr = 0.329; in connected PV+ skewness = -0.353, in connected Pyr = 1.284; kurtosis in non-connected PV+ = 0.089, in non-connected Pyr = -1.097, in connected PV+ kurtosis = -0.916, in connected Pyr kurtosis = 1.798; skewness for the PO normalized inputs in non-connected PV+ = -0.013, in non-connected Pyr = 0.890, in connected PV+ skewness = -0.370, in connected Pyr = 0.474; kurtosis for the PO normalized inputs in non-connected PV+ = -1.182, in non-connected Pyr = 0.501, in connected PV+ kurtosis = -1.377, in connected Pyr = -1.012).”

We are not aware of statistical tests designed to test the differences in normalized data. Normalized data have close estimates of centrality (Mean/Average) and dispersion (Standard Deviation/Standard Error). Hence, we only estimate the difference in shape of the normalized data distribution and report skewness and kurtosis.

-Figure 6: did some slices exhibit only connected or only non-connected pairs? If so, were the data coming from such slices included in the figure? Although unlikely it may be useful to clarify as it could affect the results.

For that figure, we had 36 slices that did not have any connected pairs, 7 slices that all had only connected pairs, and 49 slices that contained at least one of a kind (connected or not connected). Here is the figure describing correlations based on this division of data: 7 8

All the supplementary figures and tables has been changed to extended Figures and Tables

For example, Supp. Fig. 1 changed to Figure 1-1, Supp. Table 1 changed to Table 1-1.

- Figure 6: the correlation coefficient that is reported in the figure is the Pearson’s R yet bootstrapping to evaluate whether the R’s from the connected and non-connected groups are different is done on Spearman’s rho (figure legend and Supp Figure 7). Using R vs rho may not affect the results but considering that the data from this figure are central to the main finding and claim of the paper it would be better to be consistent or to justify the use of both R and rho. Thank you for pointing this out. Pearson R2 was replaced by Spearman ρ (Fig 6B,C and F,G, Fig. 7B, Fig. 8, Supplementary Fig. 4 and 5, in the text and in the legends of the Figures), which is more appropriate for the not normally distributed data such as EPSCs amplitudes for which the non-parametric tests are designed. This is revised in Lines 393-395 and 408-419 (and in the figure legends).

Minor comments

-No mention of a plan to share the data, only of the acquisition and analysis software. “The acquired data as well as the data acquisition and analysis software (M-files in Matlab format) is available upon request.” Lines 238-240.

-Figure 2: comparisons between the PO and vS1-triggered EPSC properties could be made easier if the y-axes would span the same range of values (for instance B and C would go from 0 to 15 ms). We updated the property scales for the EPSCs onset (4.5-15 for VS1 and PO) and included PV+ and Pyr cells that are not only in pairs (ONSET, VS1 inputs PV+ 50-500 ms delay protocols, n=123-125, Pyr, n=1114-115 compared to old numbers PV+ n=99, Pyr n=99; PO inputs : PV+ 50-500 ms delay protocols n=84-108, Pyr 50-500 ms delay 85-109, compared to old numbers for PV+: n=52-66, Pyr, n=59-83; RISE TIME, VS1 inputs PV+ 50-500 ms delay protocols, n=123-125, Pyr, n=114-115 compared to old numbers PV+, n=103, Pyr, n=103; DECAY TAU, VS1 inputs PV+ 50-500 ms delay protocols, n=59-110, compared to old numbers n=37-82, Pyr 50-500 ms delay protocols, n=47-102 compared to old numbers 42-86; PO inputs PV+ 50-500 ms delay protocols, n=76-98 compared to old numbers n=52-69, Pyr 50-500 ms delay protocols n=81-100, compared to old numbers n=67-85). vS1 EPSC normalized to its own peak at 250 ms delay protocol response (PV+ n=123-125; Pyr n=114-115) compared to old numbers PV+ n=101-104, Pyr n=111. PO EPSC normalized to its own peak at 250 ms delay protocol response (PV+ n=89-110; Pyr n=118) compared to old numbers PV+ n=71-87; Pyr n=87-104.

This has been corrected the legend as well.

-Figure 6, panel A: are the example traces from a connected or a non-connected pair? 9

The example traces are from non-connected pair (personal reference rg0482).

- Text from lines 376 to 387 seems misplaced. It would be better located starting at line 363, after the rest of the text describing Figure 5. Thank you. After rereading the text, it does looks more appropriate organized in that order. The text had been moved to the previous description of Figure 5. Typos:

-Line 247: “ChannelRhodopsin”.

Corrected to Channelrhodopsin, originally was devised to describe the ReaChR (Red-shifted Channel Rhodopsin)

-Legend of Figure 3: images in panel C are referred to using “top right” and “lower”, it should probably be “middle” and “right”. Thank you. This is corrected to Middle and Right.

-Line 522: “is still remains”. Thank you. This is corrected to “Whether similar results can be obtained in-vivo remains to be tested.”

-Lines 720-723: Morgenstern reference is duplicated. Thank you. We removed the duplication and corrected the text to 2016 from 2016a and 2016b. 10

In addition to the changes suggested by the Reviewers, we made the following changes to incorporate estimation statistics according to the Journal’s guidelines. Please note the list includes the places in the text and figure legends where the statistics were updated. Figure 3 (p-values to scientific format on the D-G panels) and its legend corrected, added η2 , effect size: Old D. Cumulative distribution of EPSC amplitudes comparing vS1 and PO inputs to PV+ (left) and Pyr (right) neurons in layers 2/3 (top) and 5A (bottom). EPSCs in L2/3 PV+ neurons were significantly larger from vS1 than PO (Mann-Whitney U=3596, p=1.97E-2).

Current: Should be Wilcoxon signed-rank test since those are measured in the same cells. Cumulative distribution of EPSC amplitudes comparing vS1 and PO inputs to PV+ (left) and Pyr (right) neurons in layers 2/3 (top) and 5A (bottom). EPSCs in L2/3 PV+ neurons were significantly larger from vS1 than PO (Wilcoxon signed rank test =2131, p= 5E-6, effect size η2 = Z2 /(n-1), η2 = 0.296). Old Differences between vS1 and PO EPSCs in L2/3 Pyr neurons were not significant (Mann-Whitney U=3009, p=0.44).

Current: Should be Wilcoxon signed-rank test since those are measured in the same cells. 11

Differences between vS1 and PO EPSCs in L2/3 Pyr neurons were significant (Wilcoxon signed rank test =620, p= 1.63E-4, effect size η2 = Z2 /(n-1), η2 = 0.203, however the effect size Hedges’ g and Confidence Interval show no statistical significance, extended

Statistics Table, Hedges’ g = 0.367, p=0.1 [95.0%CI -0.0344, 0.728]). Old PV+ and Pyr EPSCs amplitudes in L5A were not significantly different (PV+: Mann-Whitney U=468, p=0.44, η2 = 0.02; Pyr: Mann-Whitney U=334, p=0.6).

Current: Should be Wilcoxon signed-rank test since those are measured in the same cells. PV+ and Pyr EPSCs amplitudes in L5A between VS1 and PO were significantly different (PV+: Wilcoxon signed rank test =1, p=1E-6, η2 = 0.78, however the effect size Hedges’ g and Confidence Interval show no statistical significance, extended Statistics Table, Hedges’ g = 0.177, p=0.59, [95.0%CI -0.482, 0.888]; Pyr: Wilcoxon signed rank test =28, p= 1.8E-4, η2 = 0.56, Hedges’ g = 0.389, p=0.228, [95.0%CI -0.149, 0.823]). Old E. Cumulative distribution of EPSC amplitudes comparing input to Pyr and PV+ neurons from PO (left) and vS1 (right) to neurons in layers 2/3 (top, M-W U=2151 and 2025 (PO), p=2.34E-4 and 3.2E-2 (PO)) and 5A (bottom, M-W U=289 and 217 (PO), p=8.17E-3 and 2.88E-3 (PO)). 12

Current: Cumulative distribution of EPSC amplitudes comparing input to Pyr and PV+ neurons from PO (left) and vS1 (right) to neurons in layers 2/3 (top, M-W U=4242 and 3087 (PO), p=3E-6, η2 = 0.143 and 3.2E-2 (PO) , η2 = 0.032) and 5A (bottom, M-W U=663 and 589 (PO), p=8.17E-3, η2 = 0.115, and 2.88E-3 (PO), η2 = 0.159, however the effect size for VS1 L5A, Hedges’ g and Confidence Interval, show no statistical significance, extended Statistics Table, Hedges’ g = 0.31, p=0.22, [95.0%CI -0.338, 0.767]). Old F. Cumulative distribution of EPSC amplitudes comparing L2/3 and L5A inputs to PV+ (top) and Pyr (bottom) from PO (left) and vS1 (right) was not significantly different (PO L2/3 to L5A PV+: M-W U=1369, p=0.069; L2/3 to L5A Pyr: M-W U=897, p=0.83; vS1 L2/3 to L5A PV+: M-W U=1257, p=0.41; L2/3 to L5A Pyr: M-W U=883, p=0.11).

Current: Cumulative distribution of EPSC amplitudes comparing L2/3 and L5A inputs to PV+ (top) and Pyr (bottom) from PO (left) and vS1 (right) was not significantly different (PO L2/3 to L5A PV+: M-W U=863, p=6.9E-2, η2 = 0.032; L2/3 to L5A Pyr: M-W U=949, p=8.3E-1, η2 = 0.0005; VS1 L2/3 to L5A PV+: M-W U=1531, p=4.1E-1, η2 = 0.006; L2/3 to L5A Pyr: M-W U=1329, p=1.1E-1, η2 = 0.024). Old G. Ratio of vS1 and PO EPSCs amplitudes in PV+ interneurons divided by the EPSCs amplitudes in Pyr neurons (from D-F) shows that vS1 preferentially targets PV+ neurons 13 compared to PO confirming the results of previous study with subcellular CRACM (Okoro et al., 2022). Only pairs with both inputs included. L2/3 vS1 and PO pairs, n=30; L5A, n=12. Wilcoxon signed-rank test (Wlcxn srt =2.48, p=1.3E-2) was used since the synaptic responses are not normally distributed Kolmogorov-Smirnov and Shapiro-Wilk tests for normality (L2/3 PO inputs (PV+/Pyr) K-S(12)=0.312, p=2.02E-3, S-W(12)=0.7, p=8.35E-4; L5A vS1 inputs (PV+/Pyr), K-S(12)=0.34, p=4.15E-4, S-W(12)=0.718, p=1.26E-3. Means are shown by circles and the medians by squares. Vibrissals represent 95% confidence intervals.

Current: Ratio of vS1 and PO EPSCs amplitudes in PV+ interneurons divided by the EPSCs amplitudes in Pyr neurons (from D-F) shows that vS1 preferentially targets PV+ neurons compared to PO confirming the results of previous study with subcellular CRACM (Okoro et al., 2022). Only pairs with both inputs included. L2/3 vS1 and PO pairs, n=30; L5A, n=12. Wilcoxon signed-rank test (L2/3 ratio of VS1 vs. PO Wlcxn srt =112, p=1.3E-2, η2 = 0.212, L5A ratio of VS1 vs. PO Wlcxn srt =22, p=1.82E-1, η2 = 0.162) was used since the synaptic responses are not normally distributed Kolmogorov-Smirnov and Shapiro-Wilk tests for normality (L2/3 PO inputs (PV+/Pyr) K-S(12)=0.312, p=2.02E-3, S-W(12)=0.7, p=8.35E-4; L5A vS1 inputs (PV+/Pyr), K-S(12)=0.34, p=4.15E-4, S-W(12)=0.718, p=1.26E-3. Means are shown by circles and the medians by squares. Vibrissals represent 95% confidence intervals. Figure 7 (retested everything, corrected the p-values, removed significant difference in PO inputs between PV+ L2/3 and PV+ L5A, and between L2/3 PV+ and Pyr, since no longer significant) and its legend corrected, added η2 , effect size: Old 14 Figure 7 | Long-range inputs excite more PV+ interneurons eliciting stronger feedforward inhibition in layer-specific manner. A. Example traces showing a pair of PV+ and Pyr neurons recorded at -70 mV and at 0 mV (teal) holding potential. B. Scatter plot of EPSCs and IPSCs for vS1 inputs (upper panel), and PO (lower panel) for PV+ (left panel) and Pyr (right panel). Cortical layers 2/3 data for PV+ (red) and L5A (pink), for Pyr the L2/3 data is in black and L5A is in brown. C. vS1 and PO EPSCs (e’s) at -70 mV holding potential multiplied by -1 for the convinience of presentation, and IPSCs (I’s) at 0 mV holding potential; vS1 I’s wre significantly larger in L2/3 than L5A Pyr (independent samples Mann-Whitney (M-W) U=39, p=3.08E-2). vS1 inputs were significantly larger than PO inputs to L2/3 PV+ (M-W U=2308, p=1.97E-2). D. Averaged layer-specific Excitation to Inhibition ratio where the amplitude of EPSCs at -70 mV is divided by the amplitude of IPSCs at 0 mV for each cell. E to I ratio for vS1 and PO inputs was significantly larger in PV+ than in Pyr in L2/3 (M-W U=52 and 200.5 (PO), p=2.2E-5 and 4.66E-2 (PO)); vS1 inputs E to I ratio was significantly larger in L5A than L2/3 Pyr (M-W U=182, p=4.89E-3); PO inputs E to I ratio was significantly larger in L5A than in L2/3 PV+ (M-W U=62.5, p=2.09E-3); vS1 and PO inputs E to I ratio was significantly larger in L5A PV+ than Pyr (M-W U=49 and 41.5 (PO), p=1.83E-2 and 7.6E-5 (PO)). Correlation is estimated by the Spearman ρ correlation coefficient.

Current Figure 7 | Long-range inputs excite more PV+ interneurons eliciting stronger feedforward inhibition in layer-specific manner. A. Example traces showing a pair of PV+ and Pyr neurons recorded at -70 mV and at 0 mV (teal) holding potential. B. Scatter plot of EPSCs and IPSCs for vS1 inputs (left panel), and PO (right panel) for PV+ (upper panel) and Pyr (lower panel). Cortical layers 2/3 data for PV+ (red, 10,000 Bootsrtap Confidence Interval for Spearman’s ρ for VS1 inputs [95.0%CI -0.143, 0.770]; for PO inputs [95.0%CI 0.155, 0.824]) and L5A (pink, for VS1 inputs [95.0%CI 0.176, 0.918]; for PO inputs [95.0%CI -0.134, 0.839]) for Pyr the L2/3 data is in black (for VS1 inputs [95.0%CI 0.359, 0.913]; for PO inputs [95.0%CI 0.109, 0.847]) and L5A is in brown (for VS1 inputs [95.0%CI 0.556, 1]; for PO inputs [95.0%CI 0.166, 0.832]). C. vS1 and PO EPSCs (e’s) at -70 mV holding potential multiplied by -1 for the convinience of presentation, and IPSCs (I’s) at 0 mV holding potential; EPSCs in L2/3 PV+ neurons were significantly larger from vS1 than PO (Wilcoxon signed rank test =2131, p= 5E-6, effect size η2 = Z2 /(n-1), η2 = 0.296, Fig. 3D). vS1 I’s wre significantly larger in L2/3 than L5A Pyr (independent samples Mann-Whitney (M-W) U=39, p=3.08E-2, η2 = 0.180). D. Averaged layer-specific Excitation to Inhibition ratio where the amplitude of EPSCs at -70 mV is divided by the amplitude of IPSCs at 0 mV for each cell. E to I ratio for vS1 inputs was significantly larger in PV+ than in Pyr in L2/3 but not for PO inputs (M-W U=52 and 186.5 (PO), p=2.2E-5 and 9.37E-2 (PO), η2 = 0.409 and η2 = 0.062 (PO)); vS1 inputs E to I ratio was significantly larger in L5A than L2/3 Pyr (M-W U=182, p=4.89E-3, η2 = 0.254); vS1 and PO inputs E to I ratio was significantly larger in L5A PV+ than Pyr (M-W U=49 and 41.5 (PO), p=1.83E-2 and 4.0E-4 (PO), η2 = 0.197 and η2 = 0.344 (PO)).

Correlation is estimated by the Spearman ρ correlation coefficient. Old 15 Current 16 Figure 6 Old Figure 6 | Increased correlation of the long-range inputs to connected pairs. A. Example of voltage-clamp traces from PV+ (red) and Pyr pair with 590 nm stimulation of vS1 axons and 470 nm stimulation of PO (blue) axons middle traces are 590 nm stimulation alone and the right traces show the result of subtraction of middle traces from the first traces. Connected pairs are B.C.D.E. B. Scatter plot of vS1 in connected pairs showing a higher correlation compared to non-connected pairs in F. The data in B. and F. was resampled using 1000 samples bootstrap and produced Spearman ρ correlation coeficients, which were squared and compared with nonparametric independent samples Mann-Whitney test, U=208790.0, p<1E-10. C. Scatter plot of PO EPSCs in connected pairs showing a higher correlation compared to non-connected pairs in G. The data in C. and G. was resampled using 1000 17 samples bootstrap and produced Spearman ρ correlation coeficients, which were squared and compared with nonparametric independent samples Mann-Whitney test, U=279207.0, p<1E-10. D. Scatter plot of PO and vS1 EPSCs in the PV+ interneurons from the connected pairs. E. Scatter plot of PO and vS1 EPSCs in the Pyr neurons from connected pairs. Non-connected pairs are F.,G.,H.,I. F. Scatter plot of vS1 EPSCs from non-connected pairs (yellow). G. Scatter plot of PO (teal) EPSCs in non-connected pairs. H. Scatter plot of PO and vS1 EPSCs in the PV+ (magenta) interneurons from the non-connected pairs. I. Scatter plot of PO and vS1 EPSCs in the Pyr neurons from the non-connected pairs. Correlations are estimated by the Spearman ρ correlation coefficient.

Current Figure 6 | Increased correlation of the long-range inputs to connected pairs. A. Example of voltage-clamp traces from PV+ (red) and Pyr pair with 590 nm stimulation of vS1 axons and 470 nm stimulation of PO (blue) axons middle traces are 590 nm stimulation alone and the right traces show the result of subtraction of middle traces from the first traces. Connected pairs are B.C.D.E. B. Scatter plot of vS1 in connected pairs showing a higher correlation compared to non-connected pairs in F, also see Fig. 6-2 and Fig. 6-3 for Layer specific information. The data in B. and F. was resampled using 1000 samples bootstrap and produced Spearman ρ correlation coeficients, which were squared and compared with nonparametric independent samples Mann-Whitney test, U=208790.0, p<1E-10, Fig. 6-1. The estimation of confidence interval for the Spearmans’ ρ correlation coefficient was also done with 10,000 Bootstrap resampling, [95.0%CI 0.324, 0.760]. C. Scatter plot of PO EPSCs in connected pairs showing a higher correlation compared to non-connected pairs in G. The data in C. and G. was resampled using 1000 samples bootstrap and produced Spearman ρ correlation coeficients, which were squared and compared with nonparametric independent samples Mann-Whitney test, U=279207.0, p<1E-10, Fig. 6-1. The estimation of confidence interval for the Spearmans’ ρ correlation coefficient was also done with 10,000 Bootstrap resampling, [95.0%CI 0.163, 0.759]. D. Scatter plot of PO and vS1 EPSCs in the PV+ interneurons from the connected pairs. E. Scatter plot of PO and vS1 EPSCs in the Pyr neurons from connected pairs. Non-connected pairs are F.,G.,H.,I. F. Scatter plot of vS1 EPSCs from non-connected pairs (yellow), 10,000 Bootstrap resampling, [95.0%CI 0.097, 0.623]. G. Scatter plot of PO (teal) EPSCs in non-connected pairs, 10,000 Bootstrap resampling, [95.0%CI 0.165, 0.658]. H. Scatter plot of PO and vS1 EPSCs in the PV+ (magenta) interneurons from the non-connected pairs. I. Scatter plot of PO and vS1 EPSCs in the Pyr neurons from the non-connected pairs. Correlations are estimated by the Spearman ρ correlation coefficient. Figure 8 Old Figure 8 | Correlation of long-range excitatory and feedforward inhibitory inputs is weaker in non-connected Pyr cells. A. Scatter plots of vS1 (orange), and B. PO (blue) inputs EPSCs (x axis) and IPSCs (y axis) for connected (left panels) PV+ (red) and Pyr (black) and non-connected (right panels, magenta for PV+ and gray for Pyr). 18

Current Figure 8 | Correlation of long-range excitatory and feedforward inhibitory inputs is weaker in non-connected Pyr cells. A. Scatter plots of vS1 (orange), and B. PO (blue) inputs EPSCs (x axis) and IPSCs (y axis) for connected (left panels) PV+ (red) and Pyr (black) and non-connected (right panels, magenta for PV+ and gray for Pyr). The estimation of confidence interval for the Spearmans’ ρ correlation coefficient was also done with 10,000 Bootstrap resampling, for VS1 inputs to PV+ connected (A. upper left), [95.0%CI 0.033, 0.888]; for VS1 inputs to PV+ not connected (A. upper right), [95.0%CI 0.090, 0.962]; for VS1 inputs to Pyr connected (A. lower left), [95.0%CI 0.394, 0.978]; for VS1 inputs to Pyr not connected (A. lower right), [95.0%CI -0.377, 0.942]; for PO inputs to PV+ connected (B. upper left), [95.0%CI -0.062, 0.904]; for PO inputs to PV+ not connected (B. upper right), [95.0%CI 0.465, 0.917]; for PO inputs to Pyr connected (B. lower left), [95.0%CI 0.093, 0.947]; for PO inputs to Pyr not connected (B. lower right), [95.0%CI - 0.405, 0.640]. Figure 6-1 (Previously Supplementary Figure 5.) Old Figure 6-1 | Statistical comparison of bootstrapped vS1 and PO inputs correlation coefficients. A. Violin plots of the resampled vS1 data Spearman ρ2 correlation coefficients compared between connected and not connected pairs. B. Violin plots of the resampled PO data Spearman ρ2 correlation coefficients compared between connected and not connected pairs.

Current Figure 6-1 | Statistical comparison of bootstrapped vS1 and PO inputs correlation coefficients. A. Violin plots of the resampled vS1 data Spearman ρ2 correlation coefficients compared between connected and not connected pairs. The estimation of confidence interval for the bootstrapped Spearmans’ ρ correlation coefficients was done with 10,000 Bootstrap resampling, for VS1 inputs to connected pairs [95.0%CI 0.114, 0.711]; for VS1 inputs to not connected pairs [95.0%CI 0.072, 0.668]; the effect size = 0.242 (η2 ), is calculated based on Mann-Whitney U test comparison of connected vs. not connected pairs. B. Violin plots of the resampled PO data Spearman ρ2 correlation coefficients compared between connected and not connected pairs. The estimation of confidence interval for the bootstrapped Spearmans’ ρ correlation coefficients was done with 10,000 Bootstrap resampling, for PO inputs to connected pairs [95.0%CI 0.135, 0.664]; for PO inputs to not connected pairs [95.0%CI 0.070, 0.658]; the effect size = 0.136 (η2 ), is 19 calculated based on Mann-Whitney U test comparison of connected vs. not connected pairs. Figure 6-2 (former Supplementary Figure 2) Old Figure 6-2 | Long-range inputs have a layer-specific differential correlation for connected pairs.

A. Scatterplot of vS1 EPSCs in connected pairs in layer 2/3. B. Scatter plot of PO EPSCs in connected pairs in layer 2/3. C. Scatter plot of vS1 EPSCs in connected pairs between layer 2/3 and 5A. D. Scatter plot of PO EPSCs in connected pairs between layer 2/3 and 5A. Current Figure 6-2 | Long-range inputs have a layer-specific differential correlation for connected pairs. A. Scatterplot of vS1 EPSCs in connected pairs in layer 2/3. The estimation of confidence interval for the Spearmans’ ρ correlation coefficients was done with 10,000 Bootstrap resampling, [95.0%CI 0.291, 0.777]. B. Scatter plot of PO EPSCs in connected pairs in layer 2/3, [95.0%CI 0.064, 0.792]. C. Scatter plot of vS1 EPSCs in connected pairs between layer 2/3 and 5A, [95.0%CI -1, 1]. D. Scatter plot of PO EPSCs in connected pairs between layer 2/3 and 5A, [95.0%CI -1, 1]. Figure 6-3 (former Supplementary Figure 3) Old Figure 6-3 | Long-range inputs have a layer-specific differential correlation for non-connected pairs. A. Scatter plot of vS1 EPSCs in non-connected pairs in layer 2/3. B. Scatterplot of PO EPSCs in non-connected pairs in layer 5A.C. Scatterplot of vS1 EPSCs in non-connected pairs in layer 5A. D. Scatterplot of PO EPSCs in non-connected pairs in layer 5A,.

Current Figure 6-3 | Long-range inputs have a layer-specific differential correlation for non-connected pairs. A. Scatter plot of vS1 EPSCs in non-connected pairs in layer 2/3. The estimation of confidence interval for the Spearmans’ ρ correlation coefficients was done with 10,000 Bootstrap resampling, [95.0%CI 0.048, 0.720]. B. Scatterplot of PO EPSCs in non-connected pairs in layer 5A, [95.0%CI 0.139, 0.757]. 20 C. Scatterplot of vS1 EPSCs in non-connected pairs in layer 5A, [95.0%CI -0.406, 0.759]. D. Scatterplot of PO EPSCs in non-connected pairs in layer 5A, [95.0%CI -0.244, 0.800]

## References

[B1] Ali AB, Bannister AP, Thomson AM (1999) IPSPs elicited in CA1 pyramidal cells by putative basket cells in slices of adult rat hippocampus. Eur J Neurosci 11:1741–1753. 10.1046/j.1460-9568.1999.00592.x 10215927

[B2] Alonso JM, Martinez LM (1998) Functional connectivity between simple cells and complex cells in cat striate cortex. Nat Neurosci 1:395–403. 10.1038/1609 10196530

[B3] Alonso JM, Usrey WM, Reid RC (2001) Rules of connectivity between geniculate cells and simple cells in cat primary visual cortex. J Neurosci 21:4002–4015. 10.1523/JNEUROSCI.21-11-04002.2001 11356887 PMC6762695

[B4] Aruljothi K, Marrero K, Zhang Z, Zareian B, Zagha E (2020) Functional localization of an attenuating filter within cortex for a selective detection task in mice. J Neurosci 40:5443–5454. 10.1523/JNEUROSCI.2993-19.2020 32487695 PMC7343319

[B5] Audette NJ, Urban-Ciecko J, Matsushita M, Barth AL (2018) POm thalamocortical input drives layer-specific microcircuits in somatosensory cortex. Cereb Cortex 28:1312–1328. 10.1093/cercor/bhx044 28334225 PMC6093433

[B6] Avermann M, Tomm C, Mateo C, Gerstner W, Petersen CCH (2012) Microcircuits of excitatory and inhibitory neurons in layer 2/3 of mouse barrel cortex. J Neurophysiol 107:3116–3134. 10.1152/jn.00917.2011 22402650

[B7] Barth AL, Poulet JF (2012) Experimental evidence for sparse firing in the neocortex. Trends Neurosci 35:345–355. 10.1016/j.tins.2012.03.008 22579264

[B8] Beierlein M, Connors BW (2002) Short-term dynamics of thalamocortical and intracortical synapses onto layer 6 neurons in neocortex. J Neurophysiol 88:1924–1932. 10.1152/jn.2002.88.4.1924 12364518

[B9] Beierlein M, Gibson JR, Connors BW (2003) Two dynamically distinct inhibitory networks in layer 4 of the neocortex. J Neurophysiol 90:2987–3000. 10.1152/jn.00283.2003 12815025

[B10] Bock DD, Lee WA, Kerlin AM, Andermann ML, Hood G, Wetzel AW, Yurgenson S, Soucy ER, Kim HS, Reid RC (2011) Network anatomy and in vivo physiology of visual cortical neurons. Nature 471:177–182. 10.1038/nature09802 21390124 PMC3095821

[B11] Brown SP, Hestrin S (2009) Intracortical circuits of pyramidal neurons reflect their long-range axonal targets. Nature 457:1133–1136. 10.1038/nature07658 19151698 PMC2727746

[B12] Buxhoeveden DP, Casanova MF (2002) The minicolumn hypothesis in neuroscience. Brain 125:935–951. 10.1093/brain/awf110 11960884

[B13] Campagnola L, et al. (2022) Local connectivity and synaptic dynamics in mouse and human neocortex. Science 375:eabj5861. 10.1126/science.abj5861 35271334 PMC9970277

[B14] Cardin JA, Palmer LA, Contreras D (2007) Stimulus feature selectivity in excitatory and inhibitory neurons in primary visual cortex. J Neurosci 27:10333–10344. 10.1523/JNEUROSCI.1692-07.2007 17898205 PMC3025280

[B15] Casas-Torremocha D, Porrero C, Rodriguez-Moreno J, García-Amado M, Lübke JHR, Núñez Á, Clascá F (2019) Posterior thalamic nucleus axon terminals have different structure and functional impact in the motor and somatosensory vibrissal cortices. Brain Struct Funct 224:1627–1645. 10.1007/s00429-019-01862-4 30919051 PMC6509070

[B16] Castro-Alamancos MA, Connors BW (1997) Thalamocortical synapses. Prog Neurobiol 51:581–606. 10.1016/s0301-0082(97)00002-6 9175158

[B17] Cossell L, Iacaruso MF, Muir DR, Houlton R, Sader EN, Ko H, Hofer SB, Mrsic-Flogel TD (2015) Functional organization of excitatory synaptic strength in primary visual cortex. Nature 518:399–403. 10.1038/nature14182 25652823 PMC4843963

[B18] Cruikshank SJ, Lewis TJ, Connors BW (2007) Synaptic basis for intense thalamocortical activation of feedforward inhibitory cells in neocortex. Nat Neurosci 10:462–468. 10.1038/nn1861 17334362

[B19] Cruikshank SJ, Urabe H, Nurmikko AV, Connors BW (2010) Pathway-specific feedforward circuits between thalamus and neocortex revealed by selective optical stimulation of axons. Neuron 65:230–245. 10.1016/j.neuron.2009.12.025 20152129 PMC2826223

[B20] Dantzker JL, Callaway EM (2000) Laminar sources of synaptic input to cortical inhibitory interneurons and pyramidal neurons. Nat Neurosci 3:701–707. 10.1038/76656 10862703

[B21] Espinoza C, Guzman SJ, Zhang X, Jonas P (2018) Parvalbumin+ interneurons obey unique connectivity rules and establish a powerful lateral-inhibition microcircuit in dentate gyrus. Nat Commun 9:4605. 10.1038/s41467-018-06899-3 30389916 PMC6214995

[B22] Faber SP, Timme NM, Beggs JM, Newman EL (2019) Computation is concentrated in rich clubs of local cortical networks. Netw Neurosci 3:384–404. 10.1162/netn_a_00069 30793088 PMC6370472

[B23] Fino E, Yuste R (2011) Dense inhibitory connectivity in neocortex. Neuron 69:1188–1203. 10.1016/j.neuron.2011.02.025 21435562 PMC3086675

[B24] Fino E, Packer AM, Yuste R (2013) The logic of inhibitory connectivity in the neocortex. Neuroscientist 19:228–237. 10.1177/1073858412456743 22922685 PMC4133777

[B25] Frandolig JE, Matney CJ, Lee K, Kim J, Chevée M, Kim S, Bickert AA, Brown SP (2019) The synaptic organization of layer 6 circuits reveals inhibition as a major output of a neocortical sublamina. Cell Rep 28:3131–3143.e5. 10.1016/j.celrep.2019.08.048 31533036 PMC6941480

[B26] Gabernet L, Jadhav SP, Feldman DE, Carandini M, Scanziani M (2005) Somatosensory integration controlled by dynamic thalamocortical feed-forward inhibition. Neuron 48:315–327. 10.1016/j.neuron.2005.09.022 16242411

[B27] Gainey MA, Aman JW, Feldman DE (2018) Rapid disinhibition by adjustment of PV+ intrinsic excitability during whisker map plasticity in mouse S1. J Neurosci 38:4749–4761. 10.1523/JNEUROSCI.3628-17.2018 29678876 PMC5956988

[B28] Gibson JR, Beierlein M, Connors BW (1999) Two networks of electrically coupled inhibitory neurons in neocortex. Nature 402:75–79. 10.1038/47035 10573419

[B29] Gibson DG, Benders GA, Andrews-Pfannkoch C, Denisova EA, Baden-Tillson H, Zaveri J, Stockwell TB, Brownley A, Thomas DW, Algire MA, Merryman C, Young L, Noskov VN, Glass JI, Venter JC, Hutchison CA, Smith HO (2008) Complete chemical synthesis, assembly, and cloning of a mycoplasma genitalium genome. Science 319:1215–1220. 10.1126/science.1151721 18218864

[B30] Glickfeld LL, Andermann ML, Bonin V, Reid RC (2013) Cortico-cortical projections in mouse visual cortex are functionally target specific. Nat Neurosci 16:219–226. 10.1038/nn.3300 23292681 PMC3808876

[B31] Gonchar Y, Burkhalter A (2003) Distinct GABAergic targets of feedforward and feedback connections between lower and higher areas of rat visual cortex. J Neurosci 23:10904–10912. 10.1523/JNEUROSCI.23-34-10904.2003 14645486 PMC6740993

[B32] Gouwens NW, et al. (2020) Integrated morphoelectric and transcriptomic classification of cortical GABAergic cells. Cell 183:935–953.e19. 10.1016/j.cell.2020.09.057 33186530 PMC7781065

[B33] Guan W, Cao J, Liu L, Zhao Z, Fu Y, Yu Y (2017) Eye opening differentially modulates inhibitory synaptic transmission in the developing visual cortex. Elife 6:e32337. 10.7554/eLife.32337 29227249 PMC5746341

[B34] Gupta A, Wang Y, Markram H (2000) Organizing principles for a diversity of GABAergic interneurons and synapses in the neocortex. Science 287:273–278. 10.1126/science.287.5451.273 10634775

[B35] Hage TA, Bosma-Moody A, Baker CA, Kratz MB, Campagnola L, Jarsky T, Zeng H, Murphy GJ (2022) Synaptic connectivity to L2/3 of primary visual cortex measured by two-photon optogenetic stimulation. Elife 11:e71103. 10.7554/eLife.71103 35060903 PMC8824465

[B36] Harris JA, et al. (2019) Hierarchical organization of cortical and thalamic connectivity. Nature 575:195–202. 10.1038/s41586-019-1716-z 31666704 PMC8433044

[B37] Harris KD, Shepherd GMG (2015) The neocortical circuit: themes and variations. Nat Neurosci 18:170–181. 10.1038/nn.3917 25622573 PMC4889215

[B38] Hayashi A, Yoshida T, Ohki K (2018) Cell type specific representation of vibro-tactile stimuli in the mouse primary somatosensory cortex. Front Neural Circuits 12:109. 10.3389/fncir.2018.00109 30618647 PMC6307530

[B39] Hippenmeyer S, Vrieseling E, Sigrist M, Portmann T, Laengle C, Ladle DR, Arber S (2005) A developmental switch in the response of DRG neurons to ETS transcription factor signaling. PLoS Biol 3:e159. 10.1371/journal.pbio.0030159 15836427 PMC1084331

[B40] Hira R, Ohkubo F, Ozawa K, Isomura Y, Kitamura K, Kano M, Kasai H, Matsuzaki M (2013) Spatiotemporal dynamics of functional clusters of neurons in the mouse motor cortex during a voluntary movement. J Neurosci 33:1377–1390. 10.1523/JNEUROSCI.2550-12.2013 23345214 PMC6618743

[B41] Hirsch JA, Martinez LM, Pillai C, Alonso J, Wang Q, Sommer FT (2003) Functionally distinct inhibitory neurons at the first stage of visual cortical processing. Nat Neurosci 6:1300–1308. 10.1038/nn1152 14625553

[B42] Ho J, Tumkaya T, Aryal S, Choi H, Claridge-Chang A (2019) Moving beyond p values: data analysis with estimation graphics. Nat Methods 16:565–566. 10.1038/s41592-019-0470-3 31217592

[B43] Hofer SB, Ko H, Pichler B, Vogelstein J, Ros H, Zeng H, Lein E, Lesica NA, Mrsic-Flogel TD (2011) Differential connectivity and response dynamics of excitatory and inhibitory neurons in visual cortex. Nat Neurosci 14:1045–1052. 10.1038/nn.2876 21765421 PMC6370002

[B44] Holmgren C, Harkany T, Svennenfors B, Zilberter Y (2003) Pyramidal cell communication within local networks in layer 2/3 of rat neocortex. J Physiol 551:139–153. 10.1113/jphysiol.2003.044784 12813147 PMC2343144

[B45] Hooks BM, Hires SA, Zhang Y, Huber D, Petreanu L, Svoboda K, Shepherd GMG (2011) Laminar analysis of excitatory local circuits in vibrissal motor and sensory cortical areas. PLoS Biol 9:e1000572. 10.1371/journal.pbio.1000572 21245906 PMC3014926

[B46] Hooks BM, Mao T, Gutnisky DA, Yamawaki N, Svoboda K, Shepherd GMG (2013) Organization of cortical and thalamic input to pyramidal neurons in mouse motor cortex. J Neurosci 33:748–760. 10.1523/JNEUROSCI.4338-12.2013 23303952 PMC3710148

[B47] Hooks BM, Lin JY, Guo C, Svoboda K (2015) Dual-channel circuit mapping reveals sensorimotor convergence in the primary motor cortex. J Neurosci 35:4418–4426. 10.1523/JNEUROSCI.3741-14.2015 25762684 PMC4355205

[B48] House DRC, Elstrott J, Koh E, Chung J, Feldman DE (2011) Parallel regulation of feedforward inhibition and excitation during whisker map plasticity. Neuron 72:819–831. 10.1016/j.neuron.2011.09.008 22153377 PMC3240806

[B49] Hu H, Agmon A (2016) Differential excitation of distally versus proximally targeting cortical interneurons by unitary thalamocortical bursts. J Neurosci 36:6906–6916. 10.1523/JNEUROSCI.0739-16.2016 27358449 PMC4926238

[B50] Hubel DH, Wiesel TN (1962) Receptive fields, binocular interaction and functional architecture in the cat’s visual cortex. J Physiol 160:106–154. 10.1113/jphysiol.1962.sp006837 14449617 PMC1359523

[B51] Hubel DH, Wiesel TN (1963) Shape and arrangement of columns in cat’s striate cortex. J Physiol 165:559–568. 10.1113/jphysiol.1963.sp007079 13955384 PMC1359325

[B52] Izraeli R, Porter LL (1995) Vibrissal motor cortex in the rat: connections with the barrel field. Exp Brain Res 104:41–54. 10.1007/BF00229854 7621940

[B53] Ji X, Zingg B, Mesik L, Xiao Z, Zhang LI, Tao HW (2016) Thalamocortical innervation pattern in mouse auditory and visual cortex: laminar and cell-type specificity. Cereb Cortex 26:2612–2625. 10.1093/cercor/bhv099 25979090 PMC4869808

[B54] Jiang X, Shen S, Cadwell CR, Berens P, Sinz F, Ecker AS, Patel S, Tolias AS (2015) Principles of connectivity among morphologically defined cell types in adult neocortex. Science 350:aac9462. 10.1126/science.aac9462 26612957 PMC4809866

[B55] Jouhanneau J, Kremkow J, Poulet JFA (2018) Single synaptic inputs drive high-precision action potentials in parvalbumin expressing GABA-ergic cortical neurons in vivo. Nat Commun 9:1540. 10.1038/s41467-018-03995-2 29670095 PMC5906477

[B56] Kampa BM, Letzkus JJ, Stuart GJ (2006) Cortical feed-forward networks for binding different streams of sensory information. Nat Neurosci 9:1472–1473. 10.1038/nn1798 17099707

[B57] Kaneko T, Caria MA, Asanuma H (1994) Information processing within the motor cortex. II. Intracortical connections between neurons receiving somatosensory cortical input and motor output neurons of the cortex. J Comp Neurol 345:172–184. 10.1002/cne.903450203 7929898

[B58] Kapfer C, Glickfeld LL, Atallah BV, Scanziani M (2007) Supralinear increase of recurrent inhibition during sparse activity in the somatosensory cortex. Nat Neurosci 10:743–753. 10.1038/nn1909 17515899 PMC3518866

[B59] Katz LC, Shatz CJ (1996) Synaptic activity and the construction of cortical circuits. Science 274:1133–1138. 10.1126/science.274.5290.1133 8895456

[B60] Kätzel D, Zemelman BV, Buetfering C, Wölfel M, Miesenböck G (2011) The columnar and laminar organization of inhibitory connections to neocortical excitatory cells. Nat Neurosci 14:100–107. 10.1038/nn.2687 21076426 PMC3011044

[B61] Kawaguchi Y, Kubota Y (1997) GABAergic cell subtypes and their synaptic connections in rat frontal cortex. Cereb Cortex 7:476–486. 10.1093/cercor/7.6.476 9276173

[B62] Kawaguchi Y, Kondo S (2002) Parvalbumin, somatostatin and cholecystokinin as chemical markers for specific GABAergic interneuron types in the rat frontal cortex. J Neurocytol 31:277–287. 10.1023/a:1024126110356 12815247

[B63] Kells PA, Gautam SH, Fakhraei L, Li J, Shew WL (2019) Strong neuron-to-body coupling implies weak neuron-to-neuron coupling in motor cortex. Nat Commun 10:1575. 10.1038/s41467-019-09478-2 30952848 PMC6450901

[B64] Kerlin AM, Andermann ML, Berezovskii VK, Reid RC (2010) Broadly tuned response properties of diverse inhibitory neuron subtypes in mouse visual cortex. Neuron 67:858–871. 10.1016/j.neuron.2010.08.002 20826316 PMC3327881

[B65] Kim T, Oh WC, Choi JH, Kwon H (2016) Emergence of functional subnetworks in layer 2/3 cortex induced by sequential spikes in vivo. Proc Natl Acad Sci U S A 113:1372. 10.1073/pnas.1513410113 PMC4791016 26903616

[B66] Kiritani T, Wickersham IR, Seung HS, Shepherd GMG (2012) Hierarchical connectivity and connection-specific dynamics in the corticospinal-corticostriatal microcircuit in mouse motor cortex. J Neurosci 32:4992–5001. 10.1523/JNEUROSCI.4759-11.2012 22492054 PMC3329752

[B67] Ko H, Hofer SB, Pichler B, Buchanan KA, Sjöström PJ, Mrsic-Flogel TD (2011) Functional specificity of local synaptic connections in neocortical networks. Nature 473:87–91. 10.1038/nature09880 21478872 PMC3089591

[B68] Ko H, Cossell L, Baragli C, Antolik J, Clopath C, Hofer SB, Mrsic-Flogel TD (2013) The emergence of functional microcircuits in visual cortex. Nature 496:96–100. 10.1038/nature12015 23552948 PMC4843961

[B69] Ko H, Mrsic-Flogel TD, Hofer SB (2014) Emergence of feature-specific connectivity in cortical microcircuits in the absence of visual experience. J Neurosci 34:9812–9816. 10.1523/JNEUROSCI.0875-14.2014 25031418 PMC4099553

[B70] Komiyama T, Sato TR, O’Connor DH, Zhang Y, Huber D, Hooks BM, Gabitto M, Svoboda K (2010) Learning-related fine-scale specificity imaged in motor cortex circuits of behaving mice. Nature 464:1182–1186. 10.1038/nature08897 20376005

[B71] Lee AT, Gee SM, Vogt D, Patel T, Rubenstein JL, Sohal VS (2014) Pyramidal neurons in prefrontal cortex receive subtype-specific forms of excitation and inhibition. Neuron 81:61–68. 10.1016/j.neuron.2013.10.031 24361076 PMC3947199

[B72] Lee S, Hjerling-Leffler J, Zagha E, Fishell G, Rudy B (2010) The largest group of superficial neocortical GABAergic interneurons expresses ionotropic serotonin receptors. J Neurosci 30:16796–16808. 10.1523/JNEUROSCI.1869-10.2010 21159951 PMC3025500

[B73] Lee WA, Bonin V, Reed M, Graham BJ, Hood G, Glattfelder K, Reid RC (2016) Anatomy and function of an excitatory network in the visual cortex. Nature 532:370–374. 10.1038/nature17192 27018655 PMC4844839

[B74] Levy RB, Reyes AD (2012) Spatial profile of excitatory and inhibitory synaptic connectivity in mouse primary auditory cortex. J Neurosci 32:5609–5619. 10.1523/JNEUROSCI.5158-11.2012 22514322 PMC3359703

[B75] Li L, Ji X, Liang F, Li Y, Xiao Z, Tao HW, Zhang LI (2014) A feedforward inhibitory circuit mediates lateral refinement of sensory representation in upper layer 2/3 of mouse primary auditory cortex. J Neurosci 34:13670–13683. 10.1523/JNEUROSCI.1516-14.2014 25297094 PMC4188965

[B76] Lin JY, Knutsen PM, Muller A, Kleinfeld D, Tsien RY (2013) ReaChR: a red-shifted variant of channelrhodopsin enables deep transcranial optogenetic excitation. Nat Neurosci 16:1499–1508. 10.1038/nn.3502 23995068 PMC3793847

[B77] Ma W, Liu B, Li Y, Josh Huang Z, Zhang LI, Tao HW (2010) Visual representations by cortical somatostatin inhibitory neurons—selective but with weak and delayed responses. J Neurosci 30:14371–14379. 10.1523/JNEUROSCI.3248-10.2010 20980594 PMC3001391

[B78] Madisen L, Zwingman TA, Sunkin SM, Oh SW, Zariwala HA, Gu H, Ng LL, Palmiter RD, Hawrylycz MJ, Jones AR, Lein ES, Zeng H (2010) A robust and high-throughput Cre reporting and characterization system for the whole mouse brain. Nat Neurosci 13:133–140. 10.1038/nn.2467 20023653 PMC2840225

[B79] Mao T, Kusefoglu D, Hooks BM, Huber D, Petreanu L, Svoboda K (2011) Long-range neuronal circuits underlying the interaction between sensory and motor cortex. Neuron 72:111–123. 10.1016/j.neuron.2011.07.029 21982373 PMC5047281

[B80] Matho KS, et al. (2021) Genetic dissection of the glutamatergic neuron system in cerebral cortex. Nature 598:182–187. 10.1038/s41586-021-03955-9 34616069 PMC8494647

[B81] Moore AK, Wehr M (2013) Parvalbumin-expressing inhibitory interneurons in auditory cortex are well-tuned for frequency. J Neurosci 33:13713–13723. 10.1523/JNEUROSCI.0663-13.2013 23966693 PMC3755717

[B82] Morgenstern NA, Bourg J, Petreanu L (2016) Multilaminar networks of cortical neurons integrate common inputs from sensory thalamus. Nat Neurosci 19:1034–1040. 10.1038/nn.4339 27376765

[B83] Morishima M, Kawaguchi Y (2006) Recurrent connection patterns of corticostriatal pyramidal cells in frontal cortex. J Neurosci 26:4394–4405. 10.1523/JNEUROSCI.0252-06.2006 16624959 PMC6674016

[B84] Morishima M, Morita K, Kubota Y, Kawaguchi Y (2011) Highly differentiated projection-specific cortical subnetworks. J Neurosci 31:10380–10391. 10.1523/JNEUROSCI.0772-11.2011 21753015 PMC6623049

[B85] Morishima M, Kobayashi K, Kato S, Kobayashi K, Kawaguchi Y (2017) Segregated excitatory-inhibitory recurrent subnetworks in layer 5 of the rat frontal cortex. Cereb Cortex 27:5846–5857. 10.1093/cercor/bhx276 29045559 PMC5905586

[B86] Mountcastle VB (1997) The columnar organization of the neocortex. Brain 120:701–722. 10.1093/brain/120.4.701 9153131

[B87] Mountcastle VB (2003) Introduction. computation in cortical columns. Cereb Cortex 13:2–4. 10.1093/cercor/13.1.2 12466209

[B88] Naka A, Veit J, Shababo B, Chance RK, Risso D, Stafford D, Snyder B, Egladyous A, Chu D, Sridharan S, Mossing DP, Paninski L, Ngai J, Adesnik H (2019) Complementary networks of cortical somatostatin interneurons enforce layer specific control. Elife 8:e43696. 10.7554/eLife.43696 30883329 PMC6422636

[B89] Niell CM, Stryker MP (2008) Highly selective receptive fields in mouse visual cortex. J Neurosci 28:7520–7536. 10.1523/JNEUROSCI.0623-08.2008 18650330 PMC3040721

[B90] Nigam S, Shimono M, Ito S, Yeh F, Timme N, Myroshnychenko M, Lapish CC, Tosi Z, Hottowy P, Smith WC, Masmanidis SC, Litke AM, Sporns O, Beggs JM (2016) Rich-club organization in effective connectivity among cortical neurons. J Neurosci 36:670–684. 10.1523/JNEUROSCI.2177-15.2016 26791200 PMC4719009

[B91] Ohki K, Chung S, Ch'ng YH, Kara P, Reid RC (2005) Functional imaging with cellular resolution reveals precise micro-architecture in visual cortex. Nature 433:597–603. 10.1038/nature03274 15660108

[B92] Ohki K, Chung S, Kara P, Hübener M, Bonhoeffer T, Reid RC (2006) Highly ordered arrangement of single neurons in orientation pinwheels. Nature 442:925–928. 10.1038/nature05019 16906137

[B93] Okoro SU, Goz RU, Njeri BW, Harish M, Ruff CF, Ross SE, Gerfen CR, Hooks BM (2022) Organization of cortical and thalamic input to inhibitory neurons in mouse motor cortex. J Neurosci 42:8095–8112. 36104281 10.1523/JNEUROSCI.0950-22.2022 PMC9637002

[B94] Otsuka T, Kawaguchi Y (2008) Firing-pattern-dependent specificity of cortical excitatory feed-forward subnetworks. J Neurosci 28:11186–11195. 10.1523/JNEUROSCI.1921-08.2008 18971461 PMC6671518

[B95] Otsuka T, Kawaguchi Y (2009) Cortical inhibitory cell types differentially form intralaminar and interlaminar subnetworks with excitatory neurons. J Neurosci 29:10533–10540. 10.1523/JNEUROSCI.2219-09.2009 19710306 PMC6665698

[B96] Packer A, Yuste R (2011) Dense, unspecific connectivity of neocortical parvalbumin-positive interneurons: a canonical microcircuit for inhibition? J Neurosci 31:13260–13271. 10.1523/JNEUROSCI.3131-11.2011 21917809 PMC3178964

[B97] Pala A, Petersen CCH (2015) In vivo measurement of cell-type-specific synaptic connectivity and synaptic transmission in layer 2/3 mouse barrel cortex. Neuron 85:68–75. 10.1016/j.neuron.2014.11.025 25543458 PMC4305188

[B98] Palagina G, Meyer JF, Smirnakis SM (2019) Inhibitory units: an organizing nidus for feature-selective SubNetworks in area V1. J Neurosci 39:4931–4944. 10.1523/JNEUROSCI.2275-18.2019 30979814 PMC6670246

[B135] Paxinos G, Franklin HBJ (2004) The mouse brain in stereotaxic coordinates: compact second edition. Amsterdam: Elsevier.

[B99] Perin R, Berger TK, Markram H (2011) A synaptic organizing principle for cortical neuronal groups. Proc Natl Acad Sci U S A 108:5419–5424. 10.1073/pnas.1016051108 21383177 PMC3069183

[B100] Perin R, Telefont M, Markram H (2013) Computing the size and number of neuronal clusters in local circuits. Front Neuroanat 7:1. 10.3389/fnana.2013.00001 23423949 PMC3575568

[B101] Peron S, Pancholi R, Voelcker B, Wittenbach JD, Ólafsdóttir HF, Freeman J, Svoboda K (2020) Recurrent interactions in local cortical circuits. Nature 579:256–259. 10.1038/s41586-020-2062-x 32132709 PMC8092186

[B102] Petreanu L, Huber D, Sobczyk A, Svoboda K (2007) Channelrhodopsin-2–assisted circuit mapping of long-range callosal projections. Nat Neurosci 10:663–668. 10.1038/nn1891 17435752

[B103] Petreanu L, Mao T, Sternson SM, Svoboda K (2009) The subcellular organization of neocortical excitatory connections. Nature 457:1142–1145. 10.1038/nature07709 19151697 PMC2745650

[B104] Pfeffer CK, Xue M, He M, Huang ZJ, Scanziani M (2013) Inhibition of inhibition in visual cortex: the logic of connections between molecularly distinct interneurons. Nat Neurosci 16:1068–1076. 10.1038/nn.3446 23817549 PMC3729586

[B105] Ringach DL, Mineault PJ, Tring E, Olivas ND, Garcia-Junco-Clemente P, Trachtenberg JT (2016) Spatial clustering of tuning in mouse primary visual cortex. Nat Commun 7:12270. 10.1038/ncomms12270 27481398 PMC4974656

[B106] Rost T, Deger M, Nawrot MP (2018) Winnerless competition in clustered balanced networks: inhibitory assemblies do the trick. Biol Cybern 112:81–98. 10.1007/s00422-017-0737-7 29075845 PMC5908874

[B107] Rudy B, Fishell G, Lee S, Hjerling-Leffler J (2011) Three groups of interneurons account for nearly 100% of neocortical GABAergic neurons. Dev Neurobiol 71:45–61. 10.1002/dneu.20853 21154909 PMC3556905

[B108] Runyan CA, Schummers J, Van Wart A, Kuhlman SJ, Wilson NR, Huang ZJ, Sur M (2010) Response features of parvalbumin-expressing interneurons suggest precise roles for subtypes of inhibition in visual cortex. Neuron 67:847–857. 10.1016/j.neuron.2010.08.006 20826315 PMC2948796

[B109] Scala F, Kobak D, Bernabucci M, Bernaerts Y, Cadwell CR, Castro JR, Hartmanis L, Jiang X, Laturnus S, Miranda E, Mulherkar S, Tan ZH, Yao Z, Zeng H, Sandberg R, Berens P, Tolias AS (2021) Phenotypic variation of transcriptomic cell types in mouse motor cortex. Nature 598:144–150. 10.1038/s41586-020-2907-3 33184512 PMC8113357

[B110] Scholl B, Pattadkal JJ, Dilly GA, Priebe NJ, Zemelman BV (2015) Local integration accounts for weak selectivity of mouse neocortical parvalbumin interneurons. Neuron 87:424–436. 10.1016/j.neuron.2015.06.030 26182423 PMC4562012

[B111] Shepherd GMG, Svoboda K (2005) Laminar and columnar organization of ascending excitatory projections to layer 2/3 pyramidal neurons in rat barrel cortex. J Neurosci 25:5670–5679. 10.1523/JNEUROSCI.1173-05.2005 15958733 PMC6724876

[B112] Shepherd GMG, Pologruto TA, Svoboda K (2003) Circuit analysis of experience-dependent plasticity in the developing rat barrel cortex. Neuron 38:277–289. 10.1016/s0896-6273(03)00152-1 12718861

[B113] Shepherd GMG, Stepanyants A, Bureau I, Chklovskii D, Svoboda K (2005) Geometric and functional organization of cortical circuits. Nat Neurosci 8:782–790. 10.1038/nn1447 15880111

[B114] Sohya K, Kameyama K, Yanagawa Y, Obata K, Tsumoto T (2007) GABAergic neurons are less selective to stimulus orientation than excitatory neurons in layer II/III of visual cortex, as revealed by in vivo functional Ca2+ imaging in transgenic mice. J Neurosci 27:2145–2149. 10.1523/JNEUROSCI.4641-06.2007 17314309 PMC6673543

[B115] Song S, Sjöström PJ, Reigl M, Nelson S, Chklovskii DB (2005) Highly nonrandom features of synaptic connectivity in local cortical circuits. PLoS Biol 3:e68. 10.1371/journal.pbio.0030068 15737062 PMC1054880

[B116] Suter BA, O’Connor T, Iyer V, Petreanu LT, Hooks BM, Kiritani T, Svoboda K, Shepherd GMG (2010) Ephus: multipurpose data acquisition software for neuroscience experiments. Front Neural Circuits 4:100. 10.3389/fncir.2010.00100 21960959 PMC3176413

[B117] Taniguchi H, He M, Wu P, Kim S, Paik R, Sugino K, Kvitsiani D, Kvitsiani D, Fu Y, Lu J, Lin Y, Miyoshi G, Shima Y, Fishell G, Nelson SB, Huang ZJ (2011) A resource of Cre driver lines for genetic targeting of GABAergic neurons in cerebral cortex. Neuron 71:995–1013. 10.1016/j.neuron.2011.07.026 21943598 PMC3779648

[B118] Tezuka Y, Hagihara KM, Ohki K, Hirano T, Tagawa Y (2022) Developmental stage-specific spontaneous activity contributes to callosal axon projections. Elife 11:e72435. 10.7554/eLife.72435 36001081 PMC9402231

[B119] Thomson AM, Lamy C (2007) Functional maps of neocortical local circuitry. Front Neurosci 1:19–42. 10.3389/neuro.01.1.1.002.2007 18982117 PMC2518047

[B120] Thomson AM, Morris OT (2002) Selectivity in the inter-laminar connections made by neocortical neurones. J Neurocytol 31:239–246. 10.1023/a:1024117908539 12815243

[B121] Tremblay R, Lee S, Rudy B (2016) GABAergic interneurons in the neocortex: from cellular properties to circuits. Neuron 91:260–292. 10.1016/j.neuron.2016.06.033 27477017 PMC4980915

[B122] Vegué M, Perin R, Roxin A (2017) On the structure of cortical microcircuits inferred from small sample sizes. J Neurosci 37:8498–8510. 10.1523/JNEUROSCI.0984-17.2017 28760860 PMC6596870

[B123] Wang Y, Markram H, Goodman PH, Berger TK, Ma J, Goldman-Rakic PS (2006) Heterogeneity in the pyramidal network of the medial prefrontal cortex. Nat Neurosci 9:534–542. 10.1038/nn1670 16547512

[B124] Weiler N, Wood L, Yu J, Solla SA, Shepherd GMG (2008) Top-down laminar organization of the excitatory network in motor cortex. Nat Neurosci 11:360–366. 10.1038/nn2049 18246064 PMC2748826

[B125] Wertz A, Trenholm S, Yonehara K, Hillier D, Raics Z, Leinweber M, Szalay G, Ghanem A, Keller G, Rózsa B, Conzelmann K, Roska B (2015) Presynaptic networks. Single-cell-initiated monosynaptic tracing reveals layer-specific cortical network modules. Science 349:70–74. 10.1126/science.aab1687 26138975

[B126] Williams LE, Holtmaat A (2019) Higher-order thalamocortical inputs gate synaptic long-term potentiation via disinhibition. Neuron 101:91–102.e4. 10.1016/j.neuron.2018.10.049 30472077

[B127] Wilson DE, Smith GB, Jacob AL, Walker T, Dimidschstein J, Fishell G, Fitzpatrick D (2017) GABAergic neurons in ferret visual cortex participate in functionally specific networks. Neuron 93:1058–1065.e4. 10.1016/j.neuron.2017.02.035 28279352 PMC5477844

[B128] Xu X, Roby KD, Callaway EM (2010) Immunochemical characterization of inhibitory mouse cortical neurons: three chemically distinct classes of inhibitory cells. J Comp Neurol 518:389–404. 10.1002/cne.22229 19950390 PMC2804902

[B129] Yoshimura Y, Callaway EM (2005) Fine-scale specificity of cortical networks depends on inhibitory cell type and connectivity. Nat Neurosci 8:1552–1559. 10.1038/nn1565 16222228

[B130] Yoshimura Y, Dantzker JLM, Callaway EM (2005) Excitatory cortical neurons form fine-scale functional networks. Nature 433:868–873. 10.1038/nature03252 15729343

[B131] Yu J, Anderson CT, Kiritani T, Sheets PL, Wokosin DL, Wood L, Shepherd GMG (2008) Local-circuit phenotypes of layer 5 neurons in motor-frontal cortex of YFP-H mice. Front Neural Circuits 2:6. 10.3389/neuro.04.006.2008 19129938 PMC2614859

[B132] Zhang M, Eichhorn SW, Zingg B, Yao Z, Cotter K, Zeng H, Dong H, Zhuang X (2021) Spatially resolved cell atlas of the mouse primary motor cortex by MERFISH. Nature 598:137–143. 10.1038/s41586-021-03705-x 34616063 PMC8494645

[B133] Zingg B, Hintiryan H, Gou L, Song MY, Bay M, Bienkowski MS, Foster NN, Yamashita S, Bowman I, Toga AW, Dong H (2014) Neural networks of the mouse neocortex. Cell 156:1096–1111. 10.1016/j.cell.2014.02.023 24581503 PMC4169118

[B134] Znamenskiy P, Kim M, Muir DR, Iacaruso MF, Hofer SB, Mrsic-Flogel TD (2018) Functional selectivity and specific connectivity of inhibitory neurons in primary visual cortex. bioRxiv 294835.

