# Supplementary figures and images for: Correlated Somatosensory Input in Parvalbumin/Pyramidal Cells in Mouse Motor Cortex
Source: eNeuro. 2023 May 5;10(5):ENEURO.0488-22.2023. doi: 10.1523/ENEURO.0488-22.2023 (PMC10167893; doi:10.1523/ENEURO.0488-22.2023)

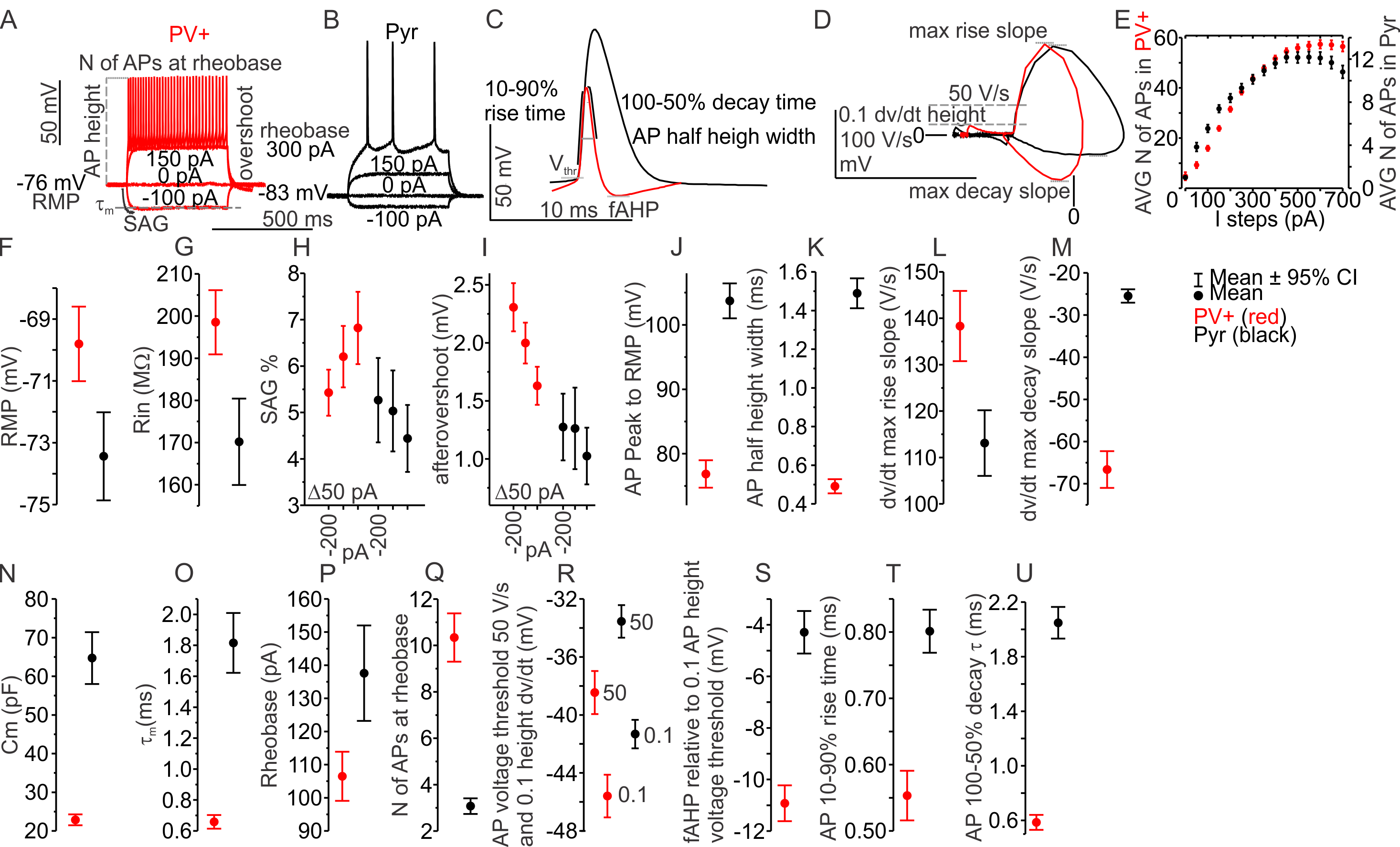

Supplement: Extended Data Figure 1-1 — Passive and active membrane properties in PV+ and Pyr neurons. A, Example current-clamp traces from PV+ (red) with current steps shown in between, resting membrane potential (RMP) to the left measured before current step application, SAG is the negative deflection below resting state to the negative current steps in percent; action potential (AP) height is measured from RMP; the rheobase is the minimal voltage required to make the neuron fire AP (300 pA); number of APs at rheobase is also recorded; overshoot is measured at the end of 500 ms negative current steps. B, Example current-clamp traces from Pyr (rheobase 300 pA). C, Example APs from PV+ and Pyr showing 10–90% rise time, voltage threshold (Vthr), fast afterhyperpolarization (fAHP); 100–50% decay time; and AP half-height width. D, The APs from C are converted to phase-space plot to show the 50 V/s voltage threshold, 0.1 dv/dt height voltage threshold; max rise slope; max decay slope. E, Input-output curve showing number of APs (average ± SEM) fired by the cells in response to the 500-ms current steps. F–U, Membrane properties. Download Figure 1-1, TIF file. [file enu-eN-NWR-0488-22-s02.tif]

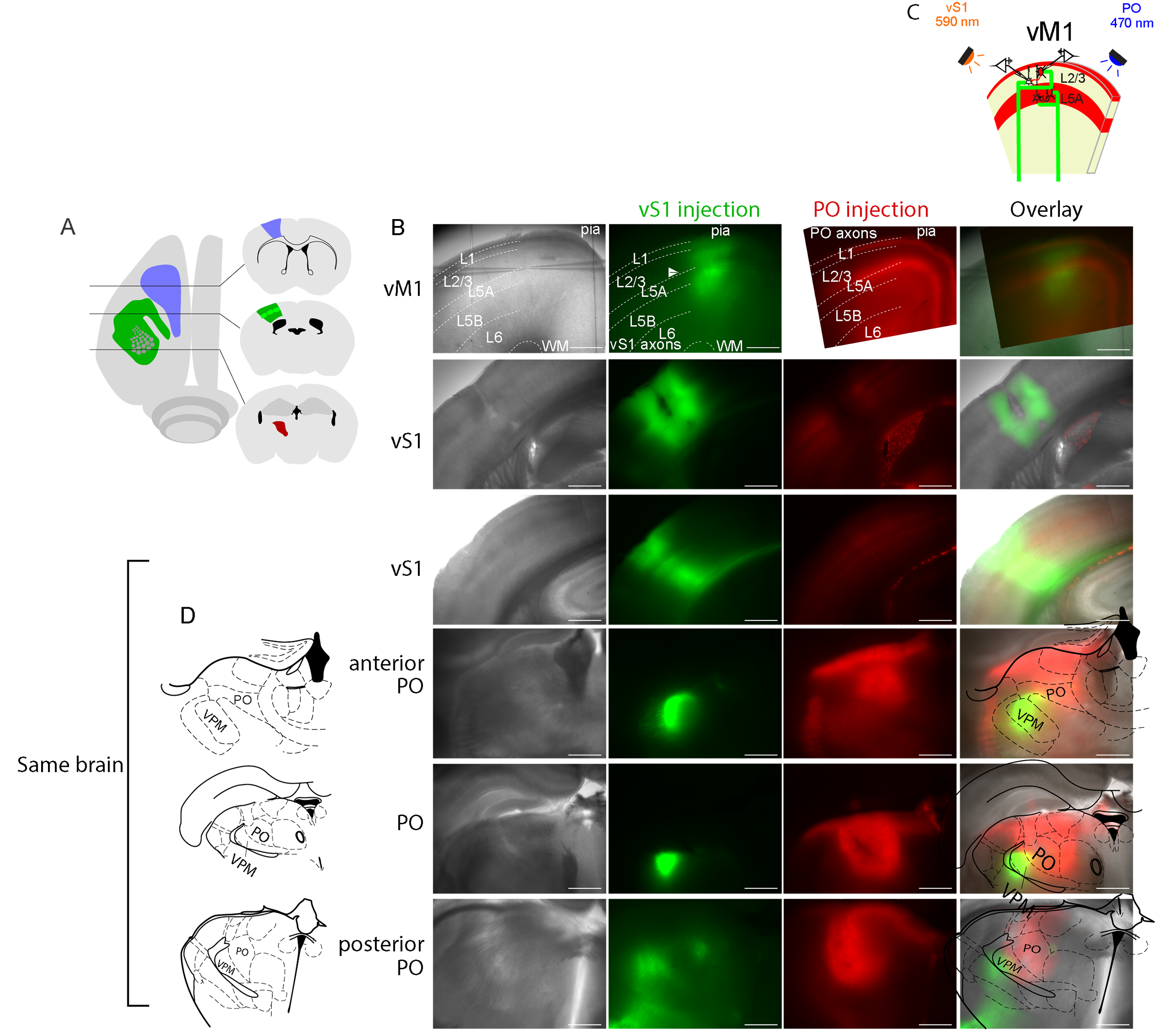

Supplement: Extended Data Figure 1-2 — Injections sites and axonal projections. A, Targeted regions (left panel). B, Example of an off-coronal 300-μm-thick brain slice of vM1 with two cells in patch-clamp. The location of the cells relative to pia and white matter. Approximate layer boundaries indicated. Second column vS1 injection site and axonal projection fluorescence in green (ReaChR-mcitrine). Third column PO injection site (ChR2-mCherry) and axonal projections fluorescence in red in L1 and L2/3 and L5A border (top, vM1 PO projections image is taken from another slice and stretched over the current slice, expressing ReaChR-mcitrine pseudo-colored in red). Fourth column is an overlay of all the previous columns and the shape of thalamic nuclei from mouse brain atlas (Paxinos), also shown in D. C, Illustration of vM1 slice with PO thalamic (red) and vS1 (green) axonal projections. D, Illustration of the thalamic nuclei shapes taken from Paxinos mouse brain atlas coordinates bregma −1.34 mm, −2.3 mm, −2.46 mm. Scale bars are 500 μm. Download Figure 1-2, TIF file. [file enu-eN-NWR-0488-22-s03.tif]

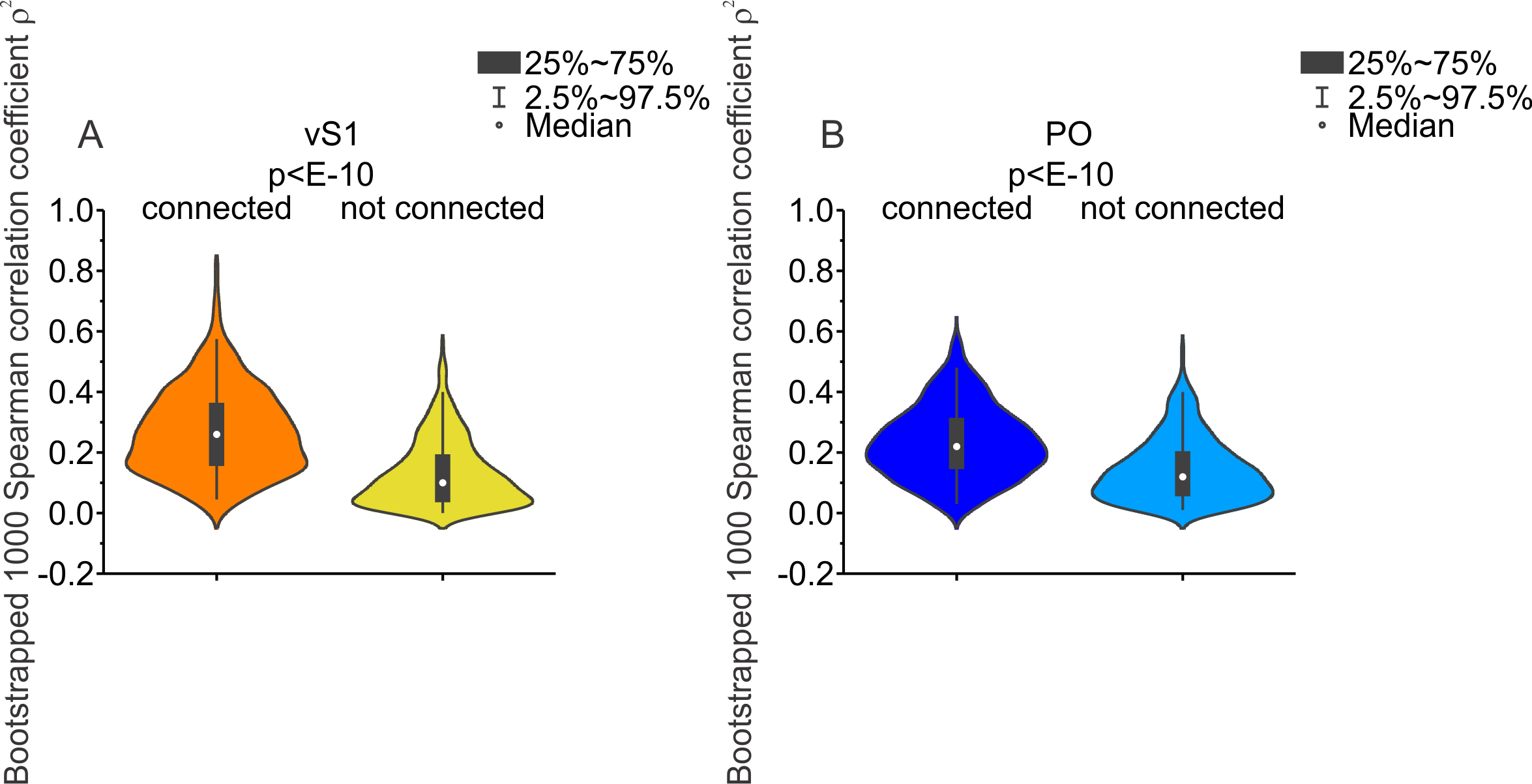

Supplement: Extended Data Figure 6-1 — Statistical comparison of bootstrapped vS1 and PO inputs correlation coefficients. A, Violin plots of the resampled vS1 data Spearman’s ρ2 correlation coefficients compared between connected and not connected pairs. The estimation of confidence interval for the bootstrapped Spearman’s ρ correlation coefficients was done with 10,000 bootstrap resampling, for VS1 inputs to connected pairs [95.0%CI 0.114, 0.711]; for VS1 inputs to not connected pairs [95.0%CI 0.072, 0.668]; the effect size = 0.242 (η2), is calculated based on Mann–Whitney U test comparison of connected versus not connected pairs. B, Violin plots of the resampled PO data Spearman’s ρ2 correlation coefficients compared between connected and not connected pairs. The estimation of confidence interval for the bootstrapped Spearman’s ρ correlation coefficients was done with 10,000 bootstrap resampling, for PO inputs to connected pairs [95.0%CI 0.135, 0.664]; for PO inputs to not connected pairs [95.0%CI 0.070, 0.658]; the effect size = 0.136 (η2), is calculated based on Mann–Whitney U test comparison of connected versus not connected pairs. Download Figure 6-1, TIF file. [file enu-eN-NWR-0488-22-s04.tif]

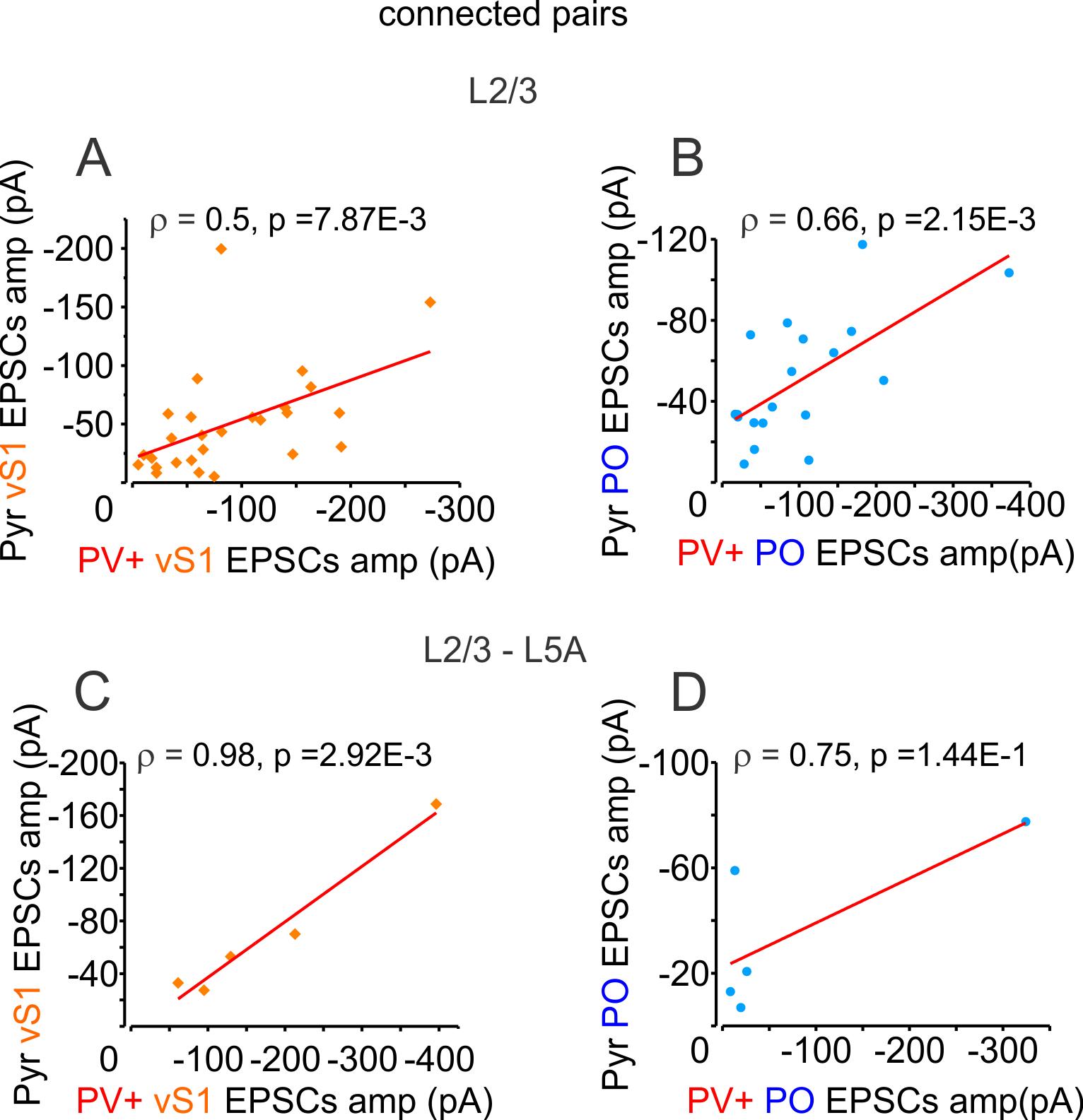

Supplement: Extended Data Figure 6-2 — Long-range inputs have a layer-specific differential correlation for connected pairs. A, Scatterplot of vS1 EPSCs in connected pairs in layer 2/3. The estimation of confidence interval for the Spearman’s ρ correlation coefficients was done with 10,000 bootstrap resampling, [95.0%CI 0.291, 0.777]. B, Scatter plot of PO EPSCs in connected pairs in layer 2/3, [95.0%CI 0.064, 0.792]. C, Scatter plot of vS1 EPSCs in connected pairs between layer 2/3 and 5A, [95.0%CI −1, 1]. D, Scatter plot of PO EPSCs in connected pairs between layer 2/3 and 5A, [95.0%CI −1, 1]. Download Figure 6-2, TIF file. [file enu-eN-NWR-0488-22-s05.tif]

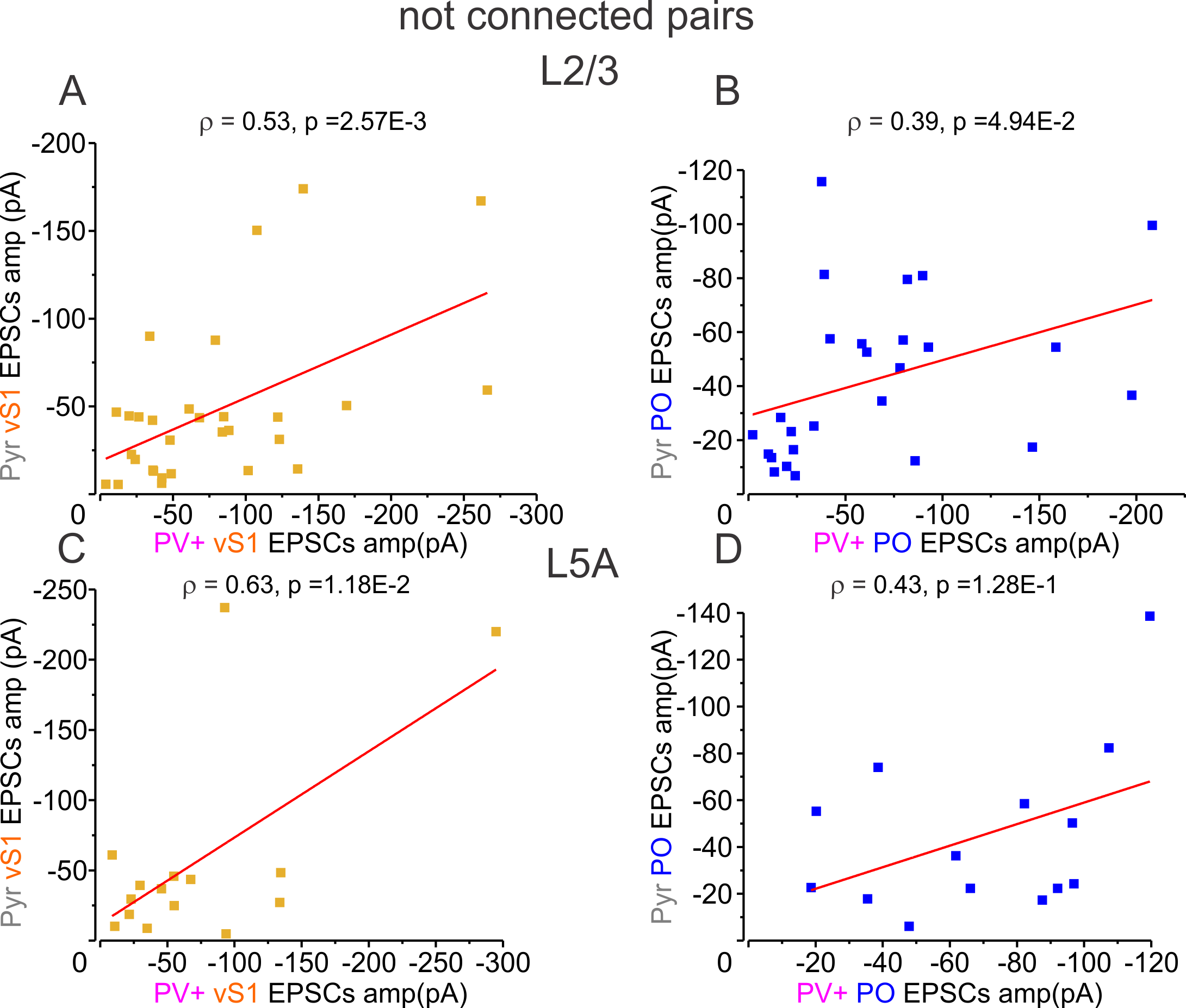

Supplement: Extended Data Figure 6-3 — Long-range inputs have a layer-specific differential correlation for nonconnected pairs. A, Scatter plot of vS1 EPSCs in nonconnected pairs in layer 2/3. The estimation of confidence interval for the Spearman’s ρ correlation coefficients was done with 10,000 bootstrap resampling, [95.0%CI 0.048, 0.720]. B, Scatterplot of PO EPSCs in nonconnected pairs in layer 5A, [95.0%CI 0.139, 0.757]. C, Scatterplot of vS1 EPSCs in nonconnected pairs in layer 5A, [95.0%CI −0.406, 0.759]. D, Scatterplot of PO EPSCs in nonconnected pairs in layer 5A, [95.0%CI −0.244, 0.800]. Download Figure 6-3, TIF file. [file enu-eN-NWR-0488-22-s06.tif]
