# Supplementary material for: Correlated Somatosensory Input in Parvalbumin/Pyramidal Cells in Mouse Motor Cortex
Source: eNeuro. 2023 May 5;10(5):ENEURO.0488-22.2023. doi: 10.1523/ENEURO.0488-22.2023 (PMC10167893; doi:10.1523/ENEURO.0488-22.2023)
Supplement: Extended Data Table 1-1 — Intrinsic Cell Properties of PV+ and Pyr Neurons in M1. Download Table 1-1, DOCX file. [file enu-eN-NWR-0488-22-s01.docx]

**Table 1-1. Intrinsic Cell Properties of PV+ and Pyr Neurons in M1**

|  |  | PV+ |  |  | Pyr |  |  |
| --- | --- | --- | --- | --- | --- | --- | --- |
|  | Units | AVG | STDEV | N | AVG | STDEV | N |
| RMP | mV | -69.80 | 7.49 | 143 | -73.44 | 8.85 | 140 |
| Rin | MΩ | 209.97 | 90.56 | 147 | 172.66 | 140.10 | 145 |
| Cm | pF | 23.01 | 8.91 | 147 | 65.04 | 42.10 | 145 |
| taum | ms | 0.66 | 0.28 | 147 | 1.83 | 1.21 | 145 |
| Rin from V-I | MΩ | 197.54 | 47.30 | 140 | 169.65 | 64.26 | 136 |
| SAG -200 pA | % | 5.44 | 3.09 | 140 | 5.30 | 5.73 | 135 |
| SAG -150 pA | % | 6.21 | 4.12 | 139 | 5.06 | 5.49 | 136 |
| SAG -100 pA | % | 6.87 | 4.91 | 140 | 4.48 | 4.54 | 136 |
| After-Overshoot Peak at 1.5 s -200 pA | mV | 2.30 | 1.27 | 135 | 1.29 | 1.81 | 131 |
| After-Overshoot Peak at 1.5 s -150 pA | mV | 1.98 | 1.07 | 134 | 1.28 | 2.21 | 132 |
| After-Overshoot Peak at 1.5 s -100 pA | mV | 1.61 | 0.97 | 136 | 1.04 | 1.54 | 134 |
| Rheobase | pA | 106.52 | 46.34 | 138 | 138.52 | 90.38 | 135 |
| N of AP at rheobase | N | 10.29 | 6.48 | 140 | 3.10 | 2.13 | 136 |
| AP at rheobase voltage thr 50 V/s | mV | -38.50 | 9.29 | 137 | -33.51 | 7.10 | 131 |
| AP at rheobase voltage thr 0.1 height dV/dt V/s | mV | -45.64 | 9.22 | 138 | -41.29 | 6.17 | 136 |
| AP at rheobase Peak to RMP | mV | 76.89 | 13.42 | 138 | 103.63 | 17.00 | 136 |
| AP at rheobase trough relative to 0.1 height dV/dt V/s thr | mV | -10.98 | 4.37 | 79 | -4.32 | 5.20 | 104 |
| dV/dt Max rise slope | V/s | 138.55 | 47.46 | 138 | 113.03 | 44.56 | 136 |
| dV/dt Max decay slope | V/s | -66.82 | 27.29 | 138 | -25.39 | 9.90 | 136 |
| AP half height width | ms | 0.49 | 0.23 | 138 | 1.49 | 0.49 | 136 |
| AP 10-90% Rise Time | ms | 0.55 | 0.23 | 138 | 0.80 | 0.20 | 134 |
| AP 100-50% Decay Time | ms | 0.58 | 0.35 | 138 | 2.05 | 0.73 | 133 |

**Table 4-1**. **Comparison of PV+ and Pyr neuron pair recording data across the literature.** Data for all pairs. The data are dominated by intralaminar connections, since PV+ to Pyr, or Pyr to PV+ interlaminar connections are more scarce. Paired or multi-paired: refers to whole-cell patch clamp of 2 or more neurons. Sharp: refers to sharp electrode intracellular recording. 2P: refers to two-photon stimulation and recording. ChR: refers to optogenetic expression of light sensitive actuator molecules (Channelrhodopsin variants), through viral vector or genetic crossing. Rubi-glut: refers to Rubi-glutamate uncaging. Intersomatic distance is reported as a measure of Euclidian distance. Reported a horizontal offset if Euclidian distance was not calculated. In area column, S – somatosensory, V – visual, M – motor. Where no distinction between the interneuron types is made, both types are included in the percentage calculation. GC – granule cells. CThN – corticothalamic neurons. CCN – corticocortical neurons. Upper layer 6a – is the top 40% of the layer height, lower layer 6a - is the bottom 40% of the layer height. FS – fast-spiking interneurons, are considered to be a part of PV+ cells, LTS- low-threshold spiking interneurons, are considered to be a part of SOM+ cells. RS – regular spiking neurons, mostly correspond to Pyr cells. Cg1/2 – prefrontal cingulate cortex area 1/2 (dACC – dorsal anterior cingulate cortex). Depressing synapses are presumed to be from PV+ interneurons.

| Report + recording method | Total (n) | Connected  (n) | % | Species | Area, Layers | Age | Intersomatic distance |
| --- | --- | --- | --- | --- | --- | --- | --- |
| Current work (paired) |  |  |  | mouse | vM1, L2/3-5b | P28-123, AVG 46 | <120 µm |
| Pyr → PV+ | 197 | 6 | 3.05 |  |  |  |  |
| PV+→Pyr | 197 | 53 | 26.9 |  |  |  |  |
| PV+ ↔Pyr | 197 | 18 | 9.14 |  |  |  |  |
| Hage et al. 2022  (paired + 2P ChR) |  |  |  | mouse | V1, L2/3-6 | P40-126, AVG 58 | <100 µm |
| Pyr → PV+ | 28 | 13 | 46 |  |  |  |  |
| PV+ → Pyr | 156,31 | 35,17 | 22.4(2P)-55 |  |  |  | <200(2P) µm |
| PV+ ↔Pyr |  |  | missing |  |  |  |  |
| Jiang et al. 2015  (multi-paired) |  |  |  | mouse | V1, L2/3-5 | ≥~P60 | <50 µm |
| Pyr → PV+ | 20(L5)-82(L2/3) | 3(L5)-13(L2/3) | 15(L5)-15.9(L2/3) |  |  |  |  |
| PV+ → Pyr | 20(L5)-82(L2/3) | 5(L5)-27(L2/3) | 25(L5)-32.9(L2/3) |  |  |  |  |
| PV+ ↔Pyr | 20(L5)-82(L2/3) | estimate based on connected Pyr (3-13) | ≤15 |  |  |  |  |
| Yoshimura & Callaway 2005 (paired) |  |  |  | rat | V1, L2/3 | P21-26 | <100 µm |
| Pyr → PV+ | 43 | 1 | 2.33 |  |  |  |  |
| PV+ → Pyr | 43 | 13 | 30.23 |  |  |  |  |
| PV+ ↔Pyr | 43 | 7 | 16.28 |  |  |  |  |
| Thomson et al. 2002  (multi-paired sharp) |  |  |  | cat | V1, L2/3-5 | not reported  (young adult) | missing |
| Pyr → PV+ & SOM+ | 25 | 2 | 8(L2/3) |  |  |  |  |
| PV+ & SOM+ → Pyr | 25 | 4 | 16(L2/3) |  |  |  |  |
| PV+ & SOM+ ↔Pyr | 25 | 3 | 12(L2/3) |  |  |  |  |
|  |  |  |  | rat | S, M, V,  L2/3-5 | not reported  (young adult) |  |
| Pyr → PV+ & SOM+ | 107 | 19 | 17.76(L2/3) |  |  |  |  |
| PV+ & SOM+ → Pyr | 107 | 14 | 13.08(L2/3) |  |  |  |  |
| PV+ & SOM+ ↔Pyr | 107 | 3 | 2.8(L2/3) |  |  |  |  |
| Beierlein et al. 2003 (paired) |  |  |  | rat | S, L4 | P14-21 | <50 µm |
| Pyr → PV+ | 172 | 74 | 43 |  |  |  |  |
| PV+ → Pyr | 190 | 83 | 44 |  |  |  |  |
| PV+ ↔Pyr | 190 | 34 | 17.89 |  |  |  |  |
| Packer and Yuste 2011 (paired &2P + Rubi-glut) |  |  |  | mouse | S, L2/3, L5, frontal L2/3 | P12-45 | <200 µm |
| Pyr → PV+ | not tested | not tested | not tested |  |  |  |  |
| PV+ → Pyr | 27  7  5  Fig. 4,5  Fig. 4,5  Fig. 4,5 | 24  6  4  Fig. 4,5  Fig. 4,5  Fig. 4,5 | 88.89  85.71  80  71(2P)  92(2P)  80(2P) |  | S,L2/3  S,L5  F, L2/3  S,L2/3  S,L5  F, L2/3 | P13-16 | <100 µm |
| PV+ ↔Pyr | not tested | not tested | not tested |  |  |  |  |
| Holmgren et al. 2003  (paired) |  |  |  | rat | V1, S, L2/3 | P14-16 | <100 µm  horizontal offset |
| Pyr → PV+ | 243 | 121-182 | 50-75 |  |  |  |  |
| PV+ → Pyr | 243 | 121-170 | 50-70 |  |  |  |  |
| PV+ ↔Pyr | 243 | 60-126 | 25-52 |  |  |  |  |
| Kapfer et al. 2007  (multi-paired) |  |  |  | rat | S, L2/3 | P23±5 (AVG±STDEV) | <50 µm |
| Pyr → PV+ | 40 | 19 | 47.5 |  |  |  |  |
| PV+ → Pyr | 39 | 26 | 66.7 |  |  |  |  |
| PV+ ↔Pyr |  |  | Not reported |  |  |  |  |
| Campagnola et al. 2021  (multi-paired) |  |  |  | mouse | V1,L2/3-L6 | P46±4.6 (AVG±STDEV) | <100 µm |
| Pyr → PV+ | 50 | 21 | 42(L2/3) |  |  |  |  |
| PV+ → Pyr | 17 | 49 | 34.69(L2/3) |  |  |  |  |
| PV+ ↔Pyr | Pooled inhibitory and normalized |  |  |  |  |  |  |
| Espinoza et al. 2018  (multi-paired) |  |  |  | mouse | Dentate Gyrus, GC cells | P20-44 | <100 µm |
| GC → PV+ | not specified | not specified | 11 |  |  |  |  |
| PV+ → GC | 1301 | 296 | 22.75 |  |  |  |  |
| PV+ ↔GC | 1301 | 32 | 2.46 |  |  |  |  |
| Frandolig et al. 2019  (paired) |  |  |  | mouse | S1, L6a | P13-47 | <100 µm |
| (CThN)Pyr → PV+ | 86 | 32 | 37 |  | Upper L6a |  |  |
| PV+ → Pyr (CThN) | 86 | 46 | 54 |  | Upper L6a |  |  |
| PV+ ↔Pyr (CThN) | 86 | 23 | 27 |  | Upper L6a |  |  |
| (CCN)Pyr → PV+ | 78 | 34 | 44 |  | Upper L6a |  |  |
| PV+ → Pyr(CCN) | 77 | 31 | 40 |  | Upper L6a |  |  |
| PV+ ↔Pyr(CCN) | 77 | 21 | 27 |  | Upper L6a |  |  |
| (CThN)Pyr → PV+ | 46 | 9 | 20 |  | Lower L6a |  |  |
| PV+ → Pyr (CThN) | 46 | 17 | 37 |  | Lower L6a |  |  |
| PV+ ↔Pyr (CThN) | 46 | 6 | 13 |  | Lower L6a |  |  |
| (CCN)Pyr → PV+ | 33 | 14 | 42 |  | Lower L6a |  |  |
| PV+ → Pyr(CCN) | 33 | 14 | 42 |  | Lower L6a |  |  |
| PV+ ↔Pyr(CCN) | 33 | 7 | 21 |  | Lower L6a |  |  |
| Gabernet et al. 2005  (paired) |  |  |  | mouse | S1,L4 | P14-25 | Not reported |
| Pyr → PV+ | not reported | not reported | not reported |  |  |  |  |
| PV+ → Pyr | not reported | 6 | ~50% |  |  |  |  |
| PV+ ↔Pyr | not reported | not reported | not reported |  |  |  |  |
| Gainey et al. 2018  (paired) |  |  |  | mouse | S1, L2/3 | P18-21 | <60 µm |
| Pyr → PV+ | not tested | not tested | not tested |  |  |  |  |
| PV+ → Pyr | 12 | 11 | 91.67 |  |  |  |  |
| PV+ ↔Pyr | not tested | not tested | not tested |  |  |  |  |
| Gibbson et al 1999  (paired) |  |  |  | rat | S1, L4, L6 | P14-21 | <50 µm |
| RS → FS & LTS | 54 | 22 | 40.74 |  |  |  |  |
| FS & LTS → RS | 71 | 37 | 52.11 |  |  |  |  |
| FS & LTS ↔RS | 54 | 5 | 9.26 |  |  |  |  |
| Guan et al. 2017  (multi-paired) |  |  |  | mouse | V1, L2/3 | P12-18 | <100 µm |
| Pyr → PV+ | not tested | not tested | not tested |  |  |  |  |
| PV+ → Pyr | 82  74 | 66  56 | 80.49  75.68 |  | (V1)  (Cg1/2) |  |  |
| PV+ ↔Pyr | not tested | not tested | not tested |  |  |  |  |
| Gupta & Markram 2005  (multi-paired) |  |  |  | rat | S1, L2-4 | P13-16 | Not reported |
| Pyr → PV+ | not tested | not tested | not tested |  |  |  |  |
| FS → Pyr (depressing) | 131 | 100 | 76.3 |  |  |  |  |
| PV+ ↔Pyr | not tested | not tested | not tested |  |  |  |  |
| Beierlein et al. 2002  (paired) |  |  |  | rat | S,L6 | P14-21 | <50 µm |
| Pyr → FS | 41 | 4 | 9.76 |  |  |  |  |
| PV+ → Pyr | not reported | not reported | not reported |  |  |  |  |
| PV+ ↔Pyr | not reported | not reported | not reported |  |  |  |  |
| Avermann et al. 2012  (multi-paired) |  |  |  | mouse | S1, L2/3 | P17-22 | <160 µm |
| Pyr → PV+(FS) | Fig. 7 | Fig. 7 | 58 |  |  |  |  |
| (FS)PV+ → Pyr | Fig. 7 | Fig. 7 | 60 |  |  |  |  |
| (FS)PV+ ↔Pyr | 36 | 12 | 33.3 |  |  |  |  |
| Hofer et al. 2011  (multi-paired) |  |  |  | mouse | V1, L2/3 | adult, not specified | < 50 µm |
| Pyr → PV+ | 41 | 36 | 87.81 |  |  |  |  |
| PV+ → Pyr | not tested | not tested | not tested |  |  |  |  |
| PV+ ↔Pyr | not tested | not tested | not tested |  |  |  |  |
| House et al. 2011  (paired) |  |  |  | rat | S1,L2/3 | P18-24 | < 150 µm |
| Pyr → FS | not tested | not tested | not tested |  |  |  |  |
| FS → Pyr | 45 | 21 | 46.7 |  |  |  |  |
| FS ↔Pyr | not tested | not tested | not tested |  |  |  |  |
| Ali et al. 1999  (paired, sharp) |  |  |  | rat | CA1 | Adults, not specified | < 200 µm |
| Pyr → FS (basket) | 263 | not reported | not reported |  |  |  |  |
| FS (basket) → Pyr | 263 | 55 | 20.91 |  |  |  |  |
| FS (basket) ↔Pyr | 263 | 2 | 0.76 |  |  |  |  |
| Jouhannneau et al. 2018  (*in-vivo* multi-paired) |  |  |  | mouse | S1, L2/3 | P21-30 | < 160 µm |
| Pyr → PV+ | 39 | 17 | 43.6 |  |  |  |  |
| PV+ →Pyr | 33 | 20 | 60.6 |  |  |  |  |
| PV+ ↔Pyr | not reported | not reported | not reported |  |  |  |  |
| Pala et al. 2015  (*in-vivo* 2P guided optogenetics in presynaptic Pyr to post PV+ & SOM) |  |  |  | mouse | S1, L2/3 | P28-56 | <125 µm |
| Pyr→ PV+ | 45 | 23 | 51 |  |  |  |  |
| PV+ →Pyr | Not tested |  |  |  |  |  |  |
| PV+ ↔Pyr | Not tested |  |  |  |  |  |  |
