# Supplementary material for: Correlated Somatosensory Input in Parvalbumin/Pyramidal Cells in Mouse Motor Cortex
Source: eNeuro. 2023 May 5;10(5):ENEURO.0488-22.2023. doi: 10.1523/ENEURO.0488-22.2023 (PMC10167893; doi:10.1523/ENEURO.0488-22.2023)
Supplement: Extended Data Table 1-2 — Estimation statistics. The website that was used to calculate Hedges’ g and confidence interval with 5000 bootstrap samples, the confidence interval is bias corrected. https://www.estimationstats.com/#/; Ho et al., 2019). η2 Calculated for nonparametric tests, η2 = Z2/(n−1) and captures % of variance of one variable reflected by the test results comparing it to another variable (0.296 -> 29.6%). While Hedges’ g estimates by how many standard deviations the two variables differ (1, means 1 SD). For Figure 3D,G, paired test was used on the website. For connected versus not connected Fisher’s exact test, the probability of not connected was subtracted from the probability of connected to calculate the effect size. Download Table 1-2, DOCX file. [file enu-eN-NWR-0488-22-s07.docx]

**Table 1-2 Estimation Statistics**

The website that was used to calculate Hedges’ g and Confidence Interval with 5000 Bootstrap samples, the confidence interval is bias corrected.

<https://www.estimationstats.com/#/>

Joses Ho, Tayfun Tumkaya, Sameer Aryal, Hyungwon Choi, Adam Claridge-Chang Moving beyond P values: data analysis with estimation graphics. Nature Methods 16, 565–566 (2019).

Etta squared calculated for non-parametric tests, η^2^ = Z^2^/(n-1) and captures % of variance of one variable reflected by the test results comparing it to another variable (0.296 -> 29.6%). While Hedges’ g estimates by how many standard deviations the two variables differ (1, means 1 Standard Deviation).

For the Fig. 3D and 3G Paired test was used on the website.

For connected vs not connected Fisher Exact the probability of not connected was subtracted from the probability of connected to calculate the effect size.

| Data Structure | Type of test | Power (effect size) |
| --- | --- | --- |
| Fig. 3D. VS1 vs PO L2/3 PV+  Not normal, Related | Wilcoxon Signed Rank | 0.296 (η^2^)  Hedges’ **g = 0.62, p=0.0004**  [95.0%CI 0.351, 0.85]. |
| Fig. 3D. VS1 vs PO L2/3 Pyr  Not normal, Related | Wilcoxon Signed Rank | 0.203 (η^2^)  Hedges’ g = 0.367, p=0.0994  [95.0%CI -0.0344, 0.728]. |
| Fig. 3D. VS1 vs PO L5A PV  Not normal, Related | Wilcoxon Signed Rank | 0.78 (η^2^)  Hedges’ g = 0.177, p=0.59  [95.0%CI -0.482, 0.888]. |
| Fig. 3D. VS1 vs PO L5A Pyr  Not normal, Related | Wilcoxon Signed Rank | 0.56 (η^2^)  Hedges’ g = 0.389, p=0.228  [95.0%CI -0.149, 0.823]. |
| Fig. 3E. VS1 PV+ vs Pyr L2/3  Not normal, Independent | Mann-Whitney U | 0.143 (η^2^)  Hedges’ **g = 0.59, p=0.0000**  [95.0%CI 0.323, 0.824]. |
| Fig. 3E. PO PV+ vs Pyr L2/3  Not normal, Independent | Mann-Whitney U | 0.032 (η^2^)  Hedges’ **g = 0.452, p=0.0076**  [95.0%CI 0.15, 0.702]. |
| Fig. 3E. VS1 PV+ vs Pyr L5A  Not normal, Independent | Mann-Whitney U | 0.115 (η^2^)  Hedges’ g = 0.31, p=0.22  [95.0%CI -0.338, 0.767]. |
| Fig. 3E. PO PV+ vs Pyr L5A  Not normal, Independent | Mann-Whitney U | 0.159 (η^2^)  Hedges’ **g = 0.804, p=0.0016**  [95.0%CI 0.37, 1.17]. |
| Fig. 3F. VS1 PV+ L2/3 vs L5A Not normal, Independent | Mann-Whitney U | 0.006 (η^2^)  Hedges’ g = 0.156, p=0.456  [95.0%CI -0.316, 0.469]. |
| Fig. 3F VS1 Pyr L2/3 vs L5A  Not normal, Independent | Mann-Whitney U | 0.024 (η^2^)  Hedges’ g = -0.058, p=0.792  [95.0%CI -0.747, 0.449]. |
| Fig. 3F. PO PV+ L2/3 vs L5A Not normal, Independent | Mann-Whitney U | 0.032 (η^2^)  Hedges’ g = -0.361, p=0.0864  [95.0%CI -0.87, 0.0723]. |
| Fig. 3F. PO Pyr L2/3 vs L5A  Not normal, Independent | Mann-Whitney U | 0.0005 (η^2^)  Hedges’ g = 0.0701, p=0.767  [95.0%CI -0.442, 0.46]. |
| Fig. 3G. PV+ to Pyr EPSCs ratio L2/3 of VS1 vs PO  Not normal, Related | Wilcoxon Signed Rank | 0.212 (η^2^)  Hedges’ **g = -0.461, p=0.0036**  [95.0%CI -0.848, -0.0911]. |
| Fig. 3G. PV+ to Pyr EPSCs ratio L5A of VS1 vs PO  Not normal, Related | Wilcoxon Signed Rank | 0.162 (η^2^)  Hedges’ g = -0.473, p=0.137  [95.0%CI -0.896, 0.0264]. |
| Fig. 4C. Normal, Independent | Fisher exact | (p_connected_ – p_not connected_)  The percent of connected Pyr when PV is connected 47 % lower than Pyr not connected when PV connected.  The percent of connected Pyr when PV not connected is 95 percent lower than Pyr not connected when PV not connected.  The percent of PV connected when Pyr connected is 45% higher than PV not connected when Pyr connected.  The percent of PV connected when Pyr not connected is 45% lower than the percent of PV not connected when Pyr not connected.  Odds Ratio = 7.059  Two-sided tail p =4.0753E-05  [95.0%CI 2.65,18.81] |
| Fig. 6B. Not normal, Independent | Spearman correlation coefficient, ρ | 10,000 Bootstrap  [95.0%CI 0.324, 0.760]. |
| Fig. 6C. Not normal, Independent | Spearman correlation coefficient, ρ | 10,000 Bootstrap  [95.0%CI 0.163, 0.759]. |
| Fig. 6F. Not normal, Independent | Spearman correlation coefficient, ρ | 10,000 Bootstrap  [95.0%CI 0.097, 0.623]. |
| Fig. 6G. Not normal, Independent | Spearman correlation coefficient, ρ | 10,000 Bootstrap  [95.0%CI 0.165, 0.658]. |
| Fig. 7B. upper left Not normal, Related | Spearman correlation coefficient, ρ | L2/3 10,000 Bootstrap  [95.0%CI -0.143, 0.770].  L5A 10,000 Bootstrap  [95.0%CI 0.176, 0.918]. |
| Fig. 7B. upper right Not normal, Related | Spearman correlation coefficient, ρ | L2/3 10,000 Bootstrap  [95.0%CI 0.155, 0.824].  L5A 10,000 Bootstrap  [95.0%CI -0.134, 0.839]. |
| Fig. 7B. lower left Not normal, Related | Spearman correlation coefficient, ρ | L2/3 10,000 Bootstrap  [95.0%CI 0.359, 0.913].  L5A 10,000 Bootstrap  [95.0%CI 0.556, 1]. |
| Fig. 7B. lower right Not normal, Related | Spearman correlation coefficient, ρ | L2/3 10,000 Bootstrap  [95.0%CI 0.109, 0.847].  L5A 10,000 Bootstrap  [95.0%CI 0.166, 0.832]. |
| Fig. 7C. Not normal, Related & VS1 I’s Pyr L2/3 vs. L5A, Independent | Wilcoxon Signed Rank & Mann-Whitney U | For VS1 vs. PO EPSCs in L2/3 PV+ please check Fig 3D. above.  VS1 I’s Pyr L2/3 vs. L5A,  0.180 (η^2^)  Hedges’ **g = -0.971, p=0.0212**  [95.0%CI -1.88, 0.124]. |
| Fig. 7D. VS1 L2/3 PV+ vs Pyr E to I ratio, Not normal, Independent | Mann-Whitney U | 0.409 (η^2^)  Hedges’ **g = -1.58, p=0.0000**  [95.0%CI -2.24, -0.906]. |
| Fig. 7D. PO L2/3 PV+ vs Pyr E to I ratio, Not normal, Independent | Mann-Whitney U | 0.062 (η^2^)  Hedges’ g = -0.451, p=0.116  [95.0%CI -1.03, 0.197]. |
| Fig. 7D. PO L2/3 PV+ vs L5A PV+ E to I ratio, Not normal, Independent | Mann-Whitney U | 0.083 (η^2^)  Hedges’ g = 0.367, p=0.233  [95.0%CI -0.22, 0.834]. |
| Fig. 7D. VS1 L2/3 Pyr vs L5A Pyr E to I ratio, Not normal, Independent | Mann-Whitney U | 0.254 (η^2^)  Hedges’ **g = 1.17, p=0.001**  [95.0%CI 0.447, 1.81]. |
| Fig. 7D. VS1 L5A PV+ vs Pyr E to I ratio, Not normal, Independent | Mann-Whitney U | 0.197 (η^2^)  Hedges’ **g = -0.943, p=0.015**  [95.0%CI -1.55, -0.188]. |
| Fig. 7D. PO L5A PV+ vs Pyr E to I ratio, Not normal, Independent | Mann-Whitney U | 0.344 (η^2^)  Hedges’ **g = -0.789, p=0.0046**  [95.0%CI -1.25, -0.376]. |
| Fig. 8A. VS1 PV+ connected e’s to I’s, Not normal, Related | Spearman correlation coefficient, ρ | 10,000 Bootstrap  [95.0%CI 0.033, 0.888]. |
| Fig. 8A. VS1 PV+ not connected e’s to I’s, Not normal, Related | Spearman correlation coefficient, ρ | 10,000 Bootstrap  [95.0%CI 0.090, 0.962]. |
| Fig. 8A. VS1 Pyr connected e’s to I’s, Not normal, Related | Spearman correlation coefficient, ρ | 10,000 Bootstrap  [95.0%CI 0.394, 0.978]. |
| Fig. 8A. VS1 Pyr not connected e’s to I’s, Not normal, Related | Spearman correlation coefficient, ρ | 10,000 Bootstrap  [95.0%CI -0.377, 0.942]. |
| Fig. 8A. PO PV+ connected e’s to I’s, Not normal, Related | Spearman correlation coefficient, ρ | 10,000 Bootstrap  [95.0%CI -0.062, 0.904]. |
| Fig. 8A. PO PV+ not connected e’s to I’s, Not normal, Related | Spearman correlation coefficient, ρ | 10,000 Bootstrap  [95.0%CI 0.465, 0.917]. |
| Fig. 8A. PO Pyr connected e’s to I’s, Not normal, Related | Spearman correlation coefficient, ρ | 10,000 Bootstrap  [95.0%CI 0.093, 0.947]. |
| Fig. 8A. PO Pyr not connected e’s to I’s, Not normal, Related | Spearman correlation coefficient, ρ | 10,000 Bootstrap  [95.0%CI -0.405, 0.640]. |
| Fig. 6-1A. Normal, Independent | Mann-Whitney U for comparison of ρ Bootstrapped Spearman correlation coefficient, ρ | 0.242 (η^2^)  10,000 Bootstrap  VS1 connected pairs  [95.0%CI 0.114, 0.711].  VS1 not connected pairs  [95.0%CI 0.072, 0.668]. |
| Fig. 6-1B. Normal, Independent | Mann-Whitney U for comparison of ρ  Bootstrapped Spearman correlation coefficient, ρ | 0.136 (η^2^)  10,000 Bootstrap  PO connected pairs  [95.0%CI 0.135, 0.664].  PO not connected pairs  [95.0%CI 0.070, 0.658]. |
| Fig. 6-2A. Not normal, Independent | Spearman correlation coefficient, ρ | 10,000 Bootstrap  [95.0%CI 0.291, 0.777]. |
| Fig. 6-2B. Not normal, Independent | Spearman correlation coefficient, ρ | 10,000 Bootstrap  [95.0%CI 0.064, 0.792]. |
| Fig. 6-2C. Not normal, Independent | Spearman correlation coefficient, ρ | 10,000 Bootstrap  [95.0%CI -1, 1]. |
| Fig. 6-2D. Not normal, Independent | Spearman correlation coefficient, ρ | 10,000 Bootstrap  [95.0%CI -1, 1]. |
| Fig. 6-3A. Not normal, Independent | Spearman correlation coefficient, ρ | 10,000 Bootstrap  [95.0%CI 0.048, 0.720]. |
| Fig. 6-3B. Not normal, Independent | Spearman correlation coefficient, ρ | 10,000 Bootstrap  [95.0%CI 0.139, 0.757]. |
| Fig. 6-3C. Not normal, Independent | Spearman correlation coefficient, ρ | 10,000 Bootstrap  [95.0%CI -0.406, 0.759]. |
| Fig. 6-3D. Not normal, Independent | Spearman correlation coefficient, ρ | 10,000 Bootstrap  [95.0%CI -0.244, 0.800]. |
|  |  |  |
